# Variation in relaxation of non‐photochemical quenching between the founder genotypes of the soybean (*Glycine max*) nested association mapping population

**DOI:** 10.1111/tpj.17219

**Published:** 2025-01-27

**Authors:** Dhananjay Gotarkar, Anthony Digrado, Yu Wang, Lynn Doran, Ignacio Sparrow‐Muñoz, Sarah Chung, Nicholas Lisa, Farwah Wasiq, Gerardo Amaro, Bethany Blakely, Brian W. Diers, Daniel J. Eck, Steven J. Burgess

**Affiliations:** ^1^ University of Minnesota Minneapolis Minnesota USA; ^2^ Global Change and Photosynthesis Research Unit USDA ARS Urbana Illinois USA; ^3^ Carl R. Woese Institute for Genomic Biology University of Illinois Urbana‐Champaign Urbana Illinois USA; ^4^ Department of Plant Biology University of Illinois Urbana‐Champaign Urbana Illinois USA; ^5^ Center for Climatic Research University of Wisconsin‐Madison Madison Wisconsin USA; ^6^ Department of Crop Sciences University of Illinois Urbana‐Champaign Urbana Illinois USA; ^7^ Department of Statistics University of Illinois Urbana‐Champaign Urbana Illinois USA

**Keywords:** non‐photochemical quenching, photosynthesis, *Glycine max*

## Abstract

Improving the efficiency of crop photosynthesis has the potential to increase yields. Genetic manipulation showed photosynthesis can be improved by speeding up the relaxation of photoprotective mechanisms during sun‐to‐shade transitions. However, it is unclear if natural variation in the relaxation of non‐photochemical quenching (NPQ) can be exploited in crop breeding programs. To address this issue, we measured six NPQ parameters in the 40 founder lines and common parent of a Soybean Nested Association Mapping (SoyNAM) panel over two field seasons in Illinois. Leaf disks were sampled from plants grown in the field, and induction and relaxation of NPQ were measured under controlled conditions. NPQ parameters did not show consistently variable trends throughout development, and variation between sampling days suggests environmental impacts on NPQ dynamics. Seventeen genotypes were found to show small but consistent differences in NPQ relaxation kinetics relative to a reference line, providing a basis for future mapping studies. Finally, a soybean canopy model predicted available phenotypic variation could result in a 1.6% difference in carbon assimilation when comparing the fastest and slowest relaxing NPQ values. No correlation could be found between yield and rates of NPQ relaxation, but a full test will require an analysis of isogenic lines.

## Significance Statement

Previous evidence suggests increasing the rate of relaxation of photoprotection can lead to improved biomass and yield. By comparing photoprotection in 41 diverse soybean genotypes grown in the field, we identify lines with faster rates of relaxation and predict a potential 1.6% difference in daily carbon assimilation which could contribute to improving soybean performance.

## INTRODUCTION

Leaves within a canopy are exposed to sunflecks and shadeflecks, caused by intermittent cloud cover, wind‐induced leaf movements, and the changing angle of the sun (Kaiser et al., 2018). Balancing variable energy supply with demand for reducing equivalents is essential for efficient photosynthesis (Kramer & Evans, 2011) and a delay in adjustment of biochemical or gas diffusional processes can lead to reduced carbon assimilation (Sakoda et al., 2022).

In response to excess light, plants activate photoprotective mechanisms (Demmig‐Adams et al., 2012; Jahns & Holzwarth, 2012) which deal with the production of reactive oxygen species and limit photoinhibition (Pinnola & Bassi, 2018). Excess absorbed light energy can be dissipated by non‐photochemical quenching (NPQ) of chlorophyll excited states, reducing the likelihood that damaging reactive oxygen species are formed (Müller et al., 2001; Ruban & Wilson, 2021). Transgenic approaches have shown increasing NPQ in rice can lead to increased biomass production in glasshouse conditions by alleviating photoinhibition (Hubbart et al., 2018). However, excess NPQ, or delays in relaxing NPQ during shadeflecks, are predicted to cause unnecessary dissipation of energy, reducing the efficiency of photosynthesis (Burgess et al., 2019; Zhu et al., 2004). As a result, speeding up NPQ activation and relaxation can improve photosynthetic efficiency (De Souza et al., 2022; Garcia‐Molina & Leister, 2020; Kromdijk et al., 2016; Lehretz et al., 2022). Support for a link between fast relaxation of NPQ and increased biomass accumulation comes from African rice genotypes grown under controlled conditions (Cowling et al., 2022). Further, analysis of transgenic plants in small‐scale field experiments suggested an increase in biomass in tobacco (Kromdijk et al., 2016) and seed production in soybean (De Souza et al., 2022) could be achieved if NPQ relaxation is accelerated. However, there have been contrasting results in Arabidopsis (Garcia‐Molina & Leister, 2020) and potato (Lehretz et al., 2022), while the manipulating NPQ on soybean seed production differed between years (De Souza et al., 2022).

Initial transgenic manipulations were based on an understanding that NPQ can be influenced by the action of three genes, including photosystem II subunit S (PsbS), zeaxanthin epoxidase (ZEP), and violaxanthin de‐epoxidase (VDE) (De Souza et al., 2022; Garcia‐Molina & Leister, 2020; Kromdijk et al., 2016; Lehretz et al., 2022). However, the relative importance of these genes was unclear, and the precise mechanism by which they contribute to NPQ formation was debated. Studies have begun to tackle these questions, suggesting that varying ZEP concentration is more important than VDE for speeding up relaxation (Küster et al., 2023), while PsbS is proposed to function by facilitating membrane thinning, leading to the clustering of light‐harvesting complexes and quenching of excitation (Wilson et al., 2024). However, NPQ kinetics remain challenging to precisely control, with both too much and too little photoprotection potentially detrimental to growth, while the intersection with other factors, such as ABA signaling, may also be important (Barrero et al., 2005; Grieco et al., 2020). Taken together, these data point to the need to further understand the relation of photoprotection to whole plant physiology if the potential benefits of altering NPQ are to be translated to commercial crop varieties (Kaiser et al., 2019; Leister, 2023).

Most experiments investigating NPQ have been performed under controlled conditions or at a single time point during development. However, NPQ is highly dynamic and sensitive to any perturbation that impacts carbon assimilation or cellular redox state. This means in a field environment, NPQ is likely to vary in response to weather conditions (Porcar‐Castell, 2011; Sun et al., 2020; Zhu et al., 2009). Two studies with field‐grown soybeans have assessed photosynthetic parameters with analysis of chlorophyll fluorescence, using canopy reflectance to calculate photochemical reflectance index (PRI) as a proxy for NPQ (Herritt et al., 2016), and OJIP transients to look at variation in fluorescence kinetics (Herritt et al., 2018). However, the individual components of NPQ relaxation were not assessed. Therefore, the extent to which individual components of NPQ co‐vary in natural populations and whether they can be selected in breeding programs remains unclear.

A nested association mapping panel has been developed for soybean with the aim of identifying beneficial alleles possessed by elite and exotic germplasm (SoyNAM) (Diers et al., 2018; Song et al., 2017). This population has proved useful for studying agronomic traits and how they impact yield (Diers et al., 2018; Lopez et al., 2019; Montes et al., 2022; Song et al., 2017), and is composed of a common parent IA3023, and 40 founder lines, including 17 high yielding elite varieties, 15 exotic lines selected for yield and diversity, and 7 plant introduction lines which yield well under drought (Song et al., 2017). We therefore sought to use this resource for analysis of NPQ parameters. Leaf disks were repeatedly sampled from field‐grown plants over the course of a field season in 2021 and 2022, NPQ kinetics were measured under controlled conditions, and a mixed‐effects linear model was used to identify lines with significantly different values for NPQ relaxation. Six parameters were calculated by fitting a double exponential function to the decay in NPQ on transition to low light (Dall'Osto et al., 2014), including fast (qE) (Krause et al., 1982) and intermediate (qM) relaxing NPQ and their respective rate constants (*τ*_qE_ and *τ*_qM_), in addition to long term NPQ (qI) and maximum NPQ reached during high light treatment (Table 1). The intermediate phase, qM, may represent a combination of several biological processes, including “qZ” caused by interconversion of xanthophyll cycle pigments (Nilkens et al., 2010), “qM” triggered by chloroplast movement (Cazzaniga et al., 2013; Dall'Osto et al., 2014), and qT, involving a shift in the balance of excitation energy between photosystems due to the movement of light‐harvesting antennae complexes from photosystem II to photosystem I, referred to as a state‐transition (Allen et al., 1981). The relevance of qM is debated (Wilson & Ruban, 2020) and qT is thought to be more important in algae than in plants (Nilkens et al., 2010) but we did not seek to differentiate between processes. Long‐term quenching (qI) is typically assumed to be the result of photoinhibition (Krause, 1988), but additional slow relaxing mechanisms such as qH have been described (Malnoë, 2018) and this process is not completely understood.

**Table 1:** List of abbreviations

Symbol	Description
*F*_v_ */F*_m_	Maximum potential quantum efficiency of Photosystem II
qE	Fast phase of NPQ relaxation, energy‐dependent quenching (<2 min)
qM	Intermediate phase of NPQ relaxation (2–30 min)
qI	Slow phase of NPQ relaxation (>30 min)
*A*_qE_	Amplitude of qE
*A*_qM_	Amplitude of qM
*A*_qI_	Amplitude of qI
*τ*_qE_	Time constant of qE relaxation
*τ*_qM_	Time constant of qM relaxation
Max. NPQ	Maximum recorded value of NPQ during the experiment
*F*_sd_	Incoming shortwave radiation
*T*_a_	Air temperature
VPD	Vapor pressure deficit
AIC	Akaike's Information Criterion

Finally, a canopy photosynthesis model (Wang et al., 2020) was used to estimate the potential impact of genetic improvement in NPQ relaxation on soybean photosynthesis including measurements of rubisco activation from (Soleh et al., 2017) in addition to canopy architecture and NPQ relaxation rates. Taken together, these data were used to (i) assess if NPQ kinetics vary in response to developmental and field environmental conditions, (ii) identify soybean genotypes with fast relaxation kinetics that could serve as the basis for genetic mapping, and (iii) test the impact of altering NPQ relaxation on carbon assimilation given existing diversity.

## RESULTS

**Figure 1:**
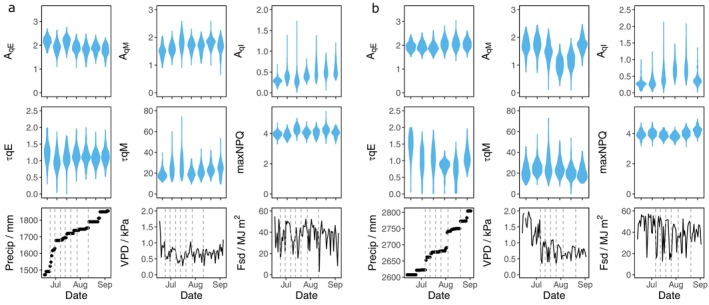
Comparison of NPQ relaxation kinetics and weather conditions over the course of two field seasons. (a) 2021 and (b) 2022. Violin plots are combined plot‐level averages for all SoyNAM genotypes on a given sampling day. Sampling dates are indicated by grey dotted lines. Precipitation is represented as cumulative values from the beginning of the year, whereas VPD and Fsd are mean daily values.

**Figure 2:**
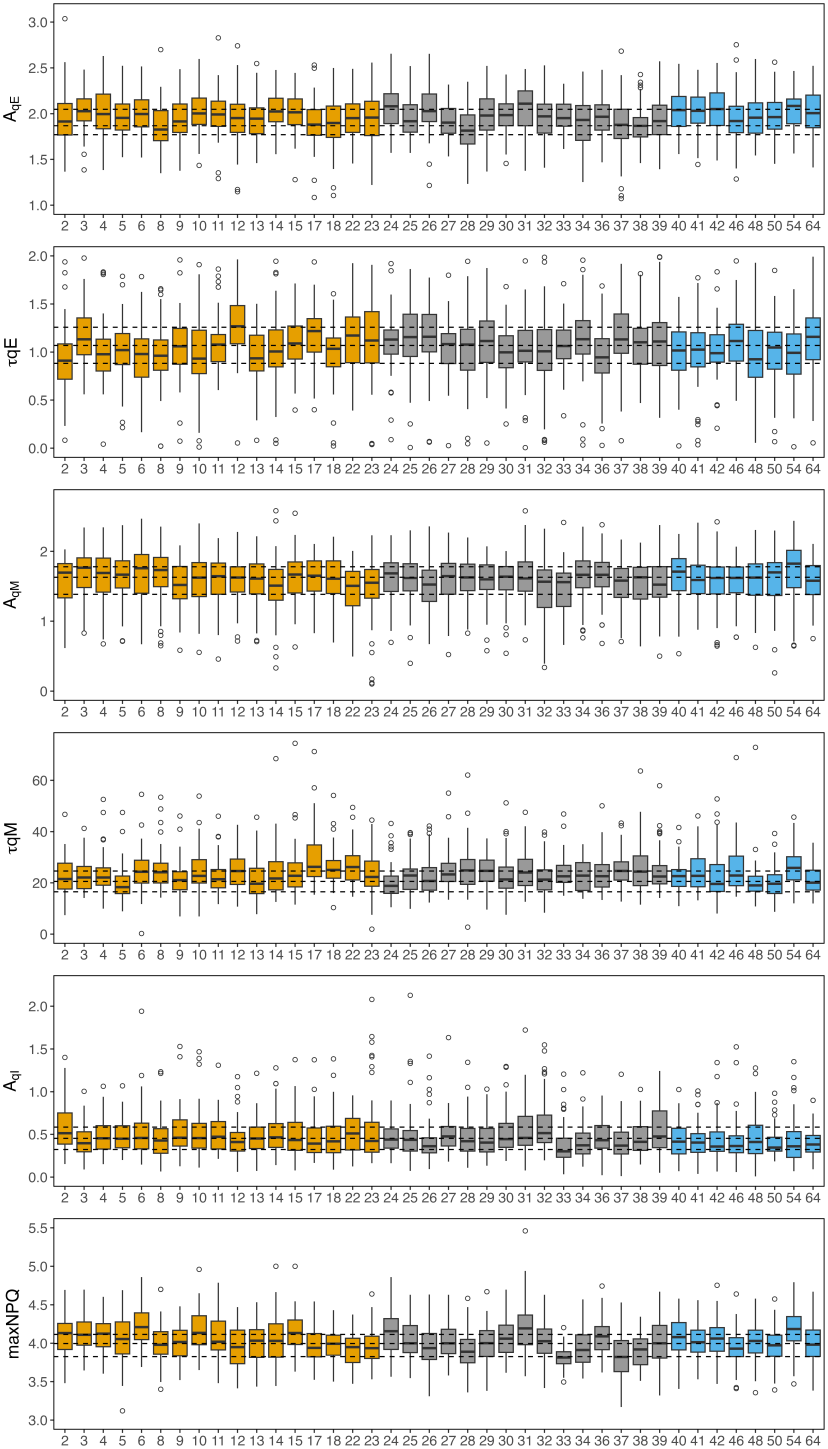
Comparison of non‐photochemical quenching (NPQ) relaxation kinetics in 41 founder genotypes of the SoyNAM population and common parent. Boxplots of six calculated NPQ relaxation parameters (*A*_qE_, *A*_qM_, *A*_qI_, *τ*_qE_, *τ*_qM_, and maximum NPQ), plots are colored based on genotype group: elite (yellow), diverse (gray), and PI (blue). Dotted black lines represent the median, upper, and lower bounds of the interquartile range of reference line RC.

Measurements of NPQ relaxation from field‐grown plants were taken between the V1 and R6 maturity stages in 2021 and 2022 (Figure 1; Tables S1). Although NPQ parameters remained largely consistent over the course of the growing season, some days exhibited differences in mean values and variance. For instance, declines in *τ*_qE_ and *A*_qM_ were observed on the fourth and fifth sampling days in 2022 (July 26th and August 4th) (Figure 1a,b). Across the duration of the experiment, the largest range in values between genotypes was seen for mean *τ*_qM_, which varied approximately 1.5‐fold between the founder genotypes of NAM5 (19.94) and NAM17 (29.3) (Figure 2; Table 2; Table S4). A narrow range of values was observed for all other parameters (Table 2; Figure S1). The mean maximum inducible NPQ using the leaf disk assay ranged from 3.81 to 4.22 per genotype, which was similar to a direct measurement of NPQt taken under high‐light in the field (Figures S2–S5).

**Table 2:** Summary of 41 NAM founder genotype NPQ relaxation parameters

Parameter	Range	Median
*A*_qE_	01.83–02.09	01.96
*τ*_qE_	00.93–01.29	01.05
*A*_qM_	01.50–01.71	01.58
*τ*_qM_	19.95–29.23	23.51
*A*_qI_	00.38–00.60	00.47
Max. NPQ	03.81–04.22	04.02

Data represent the comparison of mean values per genotype across all samples.

### Variation in NPQ relaxation parameters

**Figure 3:**
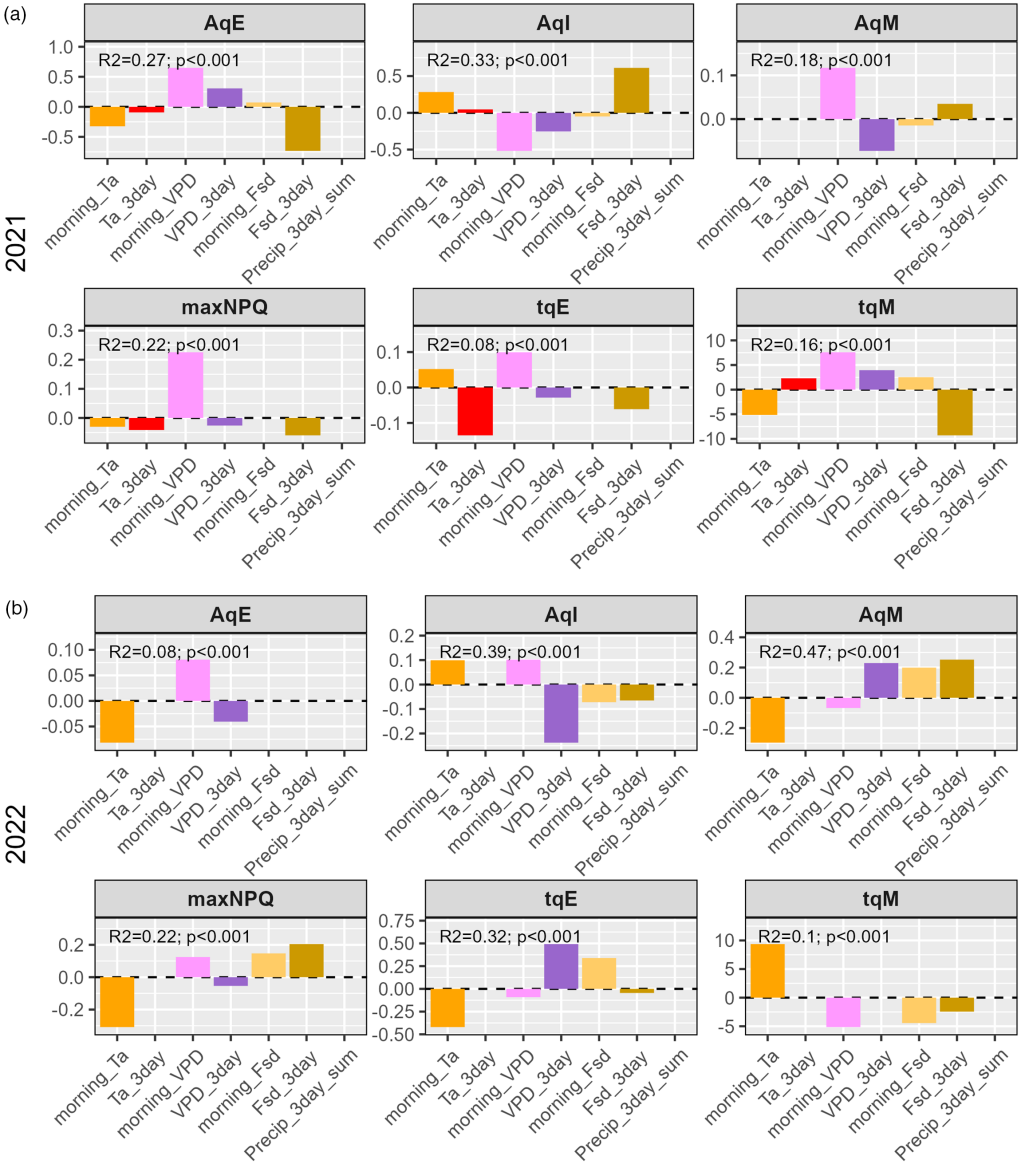
Coefficients for the best minimum adequate model (lowest AIC) for each NPQ relaxation parameter. Data from (a) 2021 and (b) 2022 are analyzed and presented separately. The R2 and *P*‐value are shown for each model.

A simple linear effects model applied to the dataset indicated significant differences between genotypes (G) and days (E) for all parameters measured (Table 3) suggesting both factors influenced NPQ. However, there was no significant G × E interaction, suggesting genotypes responded similarly to the environment (Table 3). In this model, E represents differences in both developmental stage and environmental conditions. Therefore, to further explore the source of variation, we applied a stepwise Akaike Information Criteria (AIC)‐algorithm to estimate the impact of environmental conditions on parameters. In this approach, a model is applied to the data and the impact of either removing or adding one parameter at a time is tested to see whether the fit is improved, deteriorates, or remains unchanged. Those parameters that contribute to the fit are retained and the remaining ones are removed. Following this method, we estimate that the environment explained 8–47% of the observed variance in NPQ relaxation depending on the parameter and year (Figure 3). Slow relaxing NPQ (*A*_qI_) was the parameter most consistently explained by the environment, with an *R* ^2^ > 0.3 in both years, whereas the time constant for relaxation of intermediate NPQ (*τ*_qM_) was the parameter for which the environment had the least predictive power, with a *R* ^2^ < 0.16 for both years. The *R* ^2^ for the other parameters varied between years with, for instance, the time constant for fast relaxing NPQ (*τ*_qE_) showing a *R* ^2^ of 0.08 in 2021 and 0.32 in 2022.

**Table 3:** Table of *F*‐test *P*‐values obtained by fitting an anova model for each NPQ relaxation parameter with the genotypes (G) and days (or environment, E) set as fixed factors (i.e., variable ~ Day + Genotype + Day * Genotype)

Variable	G	E	G × E
*A*_qI_	<0.001	<0.001	0.8325
*A*_qE_	<0.001	<0.001	0.9754
*τ*_qE_	<0.001	<0.001	0.1193
*A*_qM_	<0.001	<0.001	0.9613
*τ*_qM_	<0.001	<0.001	0.9463
Max. NPQ	<0.001	<0.001	0.8849

Both NPQ parameters (Figure S6) and weather variables (Figure S7) are related. Maximum NPQ is a composite of *A*_qE_, *A*_qM_, and *A*_qI_, and inversely correlated with the time constant of fast relaxing NPQ (*τ*_qE_), meaning the qE relaxed faster the greater the maximum value of NPQ (Figure S6). Vapor pressure deficit (VPD), which is the difference between the amount of moisture in the air and how much it can hold when saturated, is linked to air temperature, precipitation, and solar irradiation, with for instance sunny, hot, and dry days associated with higher VPD (Figure S7). As a result, there are similarities between how variables respond to the environment. In 2021 the large coefficients for vapor pressure deficit (VPD_3day and morning_VPD), and three‐day average daily solar irradiation (Fsd_3day), indicate their strong impact on parameters (Figure 3). While in 2022, 3‐day average vapor pressure deficit (VPD_3day) and morning air temperature (*T*_a_) were the most important. When years were compared separately, 3‐day cumulative precipitation (Precip_3day_sum) and 3‐day mean daily temperatures (Ta_3day) appeared to be the least influential. Three‐day average daily temperatures (Ta_3day) was negatively associated with *τ*_qE_ in 2021, but environment only explained a small amount of variation in this parameter *R* ^2^ = 0.08. When the 2 years were combined in a single model, precipitation (Precip_3day_sum), and 3‐day average temperatures (Ta_3day) were more important than when considered for each year independently (Figure S8). The lack of impact of precipitation or temperature on the 2021 and 2022 model suggests that these variables mostly contributed to the year‐to‐year variation between measurements, rather than within season sampling days.

Across years maximum NPQ and *A*_qE_ were consistently associated with high VPD (morning_VPD), and *A*_qI_ was negatively associated with 3‐day average VPD (VPD_3day) (Figure 3). *A*_qM_, *τ*_qE_, and *τ*_qM_ were affected differently by the environment each year, showing opposing associations with morning temperature (morning_Ta) and VPD (morning_VPD and VPD_3day) in 2021 and 2022. However, these data should be interpreted in the context that environmental variables had a limited influence over these parameters in 2021 (*R* ^2^ < 0.18). Those 2 years also showed different meteorological patterns, which could explain why some variables had more influence in 1 year but not another. In sum, variation in NPQ parameters was correlated with changes in weather variables, and VPD, along with parameters that co‐vary, had the largest impact.

**Figure 4:**
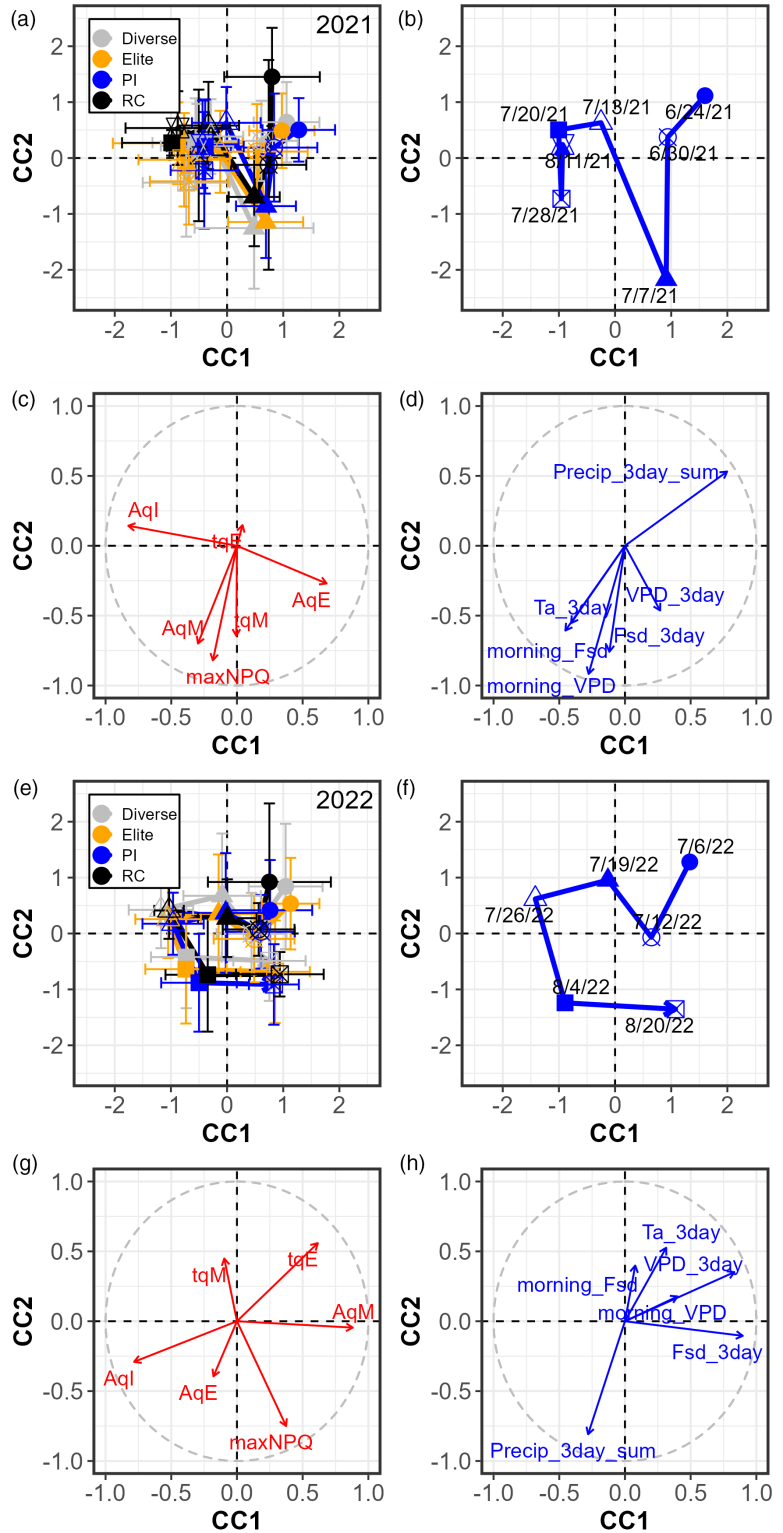
Canonical correlation analysis (CCA) displaying the relationships between the NPQ relaxation parameters and the environmental variables. (a–d) 2021 (e–h) 2022. (a, b, e, f) CCA showing the spatial distribution of the different observations on the canonical axis (CC). The average (±standard deviation) for the different NAM groups (with diverse, elite, PI, and RC groups in grey, orange, blue and black, respectively) at different days is represented by different shapes. For each group, a solid line connects those points to represent their evolution throughout the season. (c, d, g, h) The correlation circle showing the relationships between variables. Variables related to the NPQ relaxation parameters, and the environments are represented in red and blue, respectively. The canonical correlation and associated statistics are shown in Tables S5 and S6.

To further explore the relationship between NPQ parameters and meteorological conditions, as well as the interdependence within those respective sets of variables, we performed a canonical correlation analysis (CCA) (Figure 4). Morning temperature (morning_Ta) was excluded from the analysis because it was highly correlated with 3‐day average temperature (Ta_3day) and solar irradiation (morning_Fsd). For both years, CCA revealed seasonal patterns in NPQ relaxation parameters (Figure 4). In 2021, the CCA showed a change in environmental conditions throughout the season, characterized by lower precipitation (Precip_3day_sum) between June 24th and July 20th (Figure 4), and a general increase in temperature (*T*_a_) and irradiation (morning_F_sd_). This is evidenced by a movement of the dataset from right to left quadrants (Figure 4b) in the opposite direction from Precip_3day_sum (Figure 4d). The axes for the two datasets (i.e., NPQ and environment) were strongly correlated with each other (Table S5), allowing us to associate changes in the environment with changes in NPQ relaxation. Increases in *A*_qI_ were accompanied by a decline in *A*_qE_ (Figure 4c), while the rate of relaxation of *A*_qE_ remained constant (*τ*_qE_) (Figure 4d). This is in accordance with the AIC analysis which showed *τ*_qE_ had a low predictive power over NPQ parameters in 2021 (Figure 3). July 7th distinguished itself from the other days due to high 3‐day averages for irradiation and VPD (Fsd_3days and VPD_3day) as would occur during a particularly dry spell. This was associated with an increase in *A*_qM_, maximum NPQ, and *τ*_qM_. Past July 20th, the environmental conditions showed less variation, and this was associated with more constant NPQ parameters (Figure 4).

The CCA for 2022 showed a similar pattern, with an increase in *A*_qI_ at the beginning of the growing season, as evidenced by the dataset moving from the bottom right quadrant to the bottom left (in the same direction as *A*_qI_) (from July 6th to July 26th), but this was instead accompanied by a decline in *A*_qM_ rather than *A*_qE_ (Figure 4f–h). As with 2021, the axes for the two datasets (i.e., NPQ and environment) were strongly associated with each other (Table S6), allowing us to correlate changes in the environment with changes in NPQ relaxation. The transition from July to August was marked by increasing precipitation and lower temperatures (Ta_3day) which was associated with increasing maximum NPQ and a decline in *τ*_qM_. Between August 4th and August 20th, the opposite behavior of environmental variables was observed, accompanied by an increase in *A*_qM_ and a decline in *A*_qI_.

### Variation in NPQ parameters between NAM founder groups

To test for variation between the NAM founders within a day, a principal component analysis (PCA) was run for each day (Figures S8–S20). While a CCA builds its first component to maximize the correlation between the two datasets, a PCA builds its first component to maximize the variance. The results showed no distinction between the Diverse, Elite, PI, and RC groups, which tended to overlap (Figures S9–S22). To determine if the groups were significantly distinct from each other, a linear model followed by an ANOVA was used on the principal component coordinate with the “NAM groups” set as a fixed factor. This analysis showed some significant differences between groups on some dates (Table S7). For example, groups clusters separated on August 4, 2022 on PC2 (Figure S20), and PC3 on July 13, 2021 (Figure S12) and July 6, 2022 (Figure S16). This suggests that some groups distinguished themselves from the others on the parameters that were the most correlated with these axes. For instance, on August 4, 2022, groups separated on the second component, with PIs showing higher values in *A*_qE_ and maximum NPQ on average (Figure S20). Overall, results from the PCA analysis are in good agreement with findings from the CCA. Both revealed no striking differences between the different NAM groups as they showed the same seasonal behavior in both years, which suggests a stronger impact of the environment on NPQ kinetics than genotypic variation.

### Variation in NPQ parameters between NAM founders

**Figure 5:**
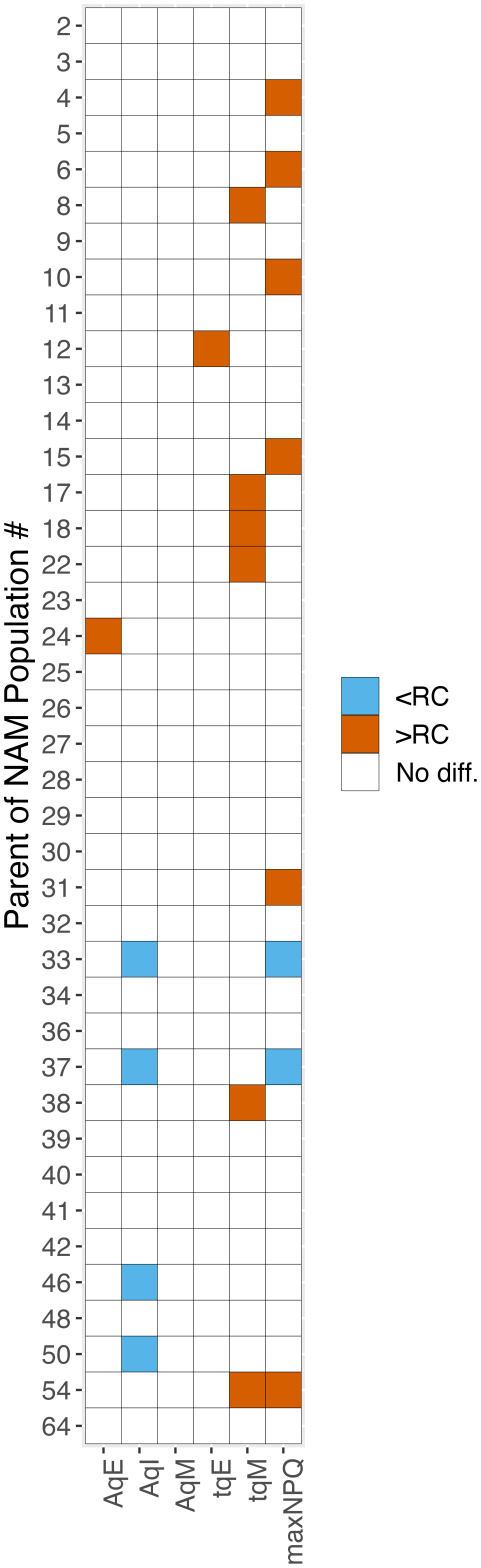
Summary representation of AIC trials comparing NAM founder genotypes with the common parent (RC) based on the estimated regression coefficients from the full model. Genotypes that showed a positive (orange) or negative (blue) difference relative to RC, and where the full model had a lower AIC than the null model across all six modeling scenarios are indicated. White cells represent instances when the null model, which assumes no difference from the RC baseline, had a lower AIC than its corresponding full model or there were disagreements of the sign difference relative to RC in one or more comparisons. Larger values for *τ*_qE_ and *τ*_qM_ represent slower relaxation.

To explore the relationship between genotype and NPQ kinetics further, a mixed effects linear model was employed to test whether genotypes showed consistent variation in NPQ parameters relative to the reference genotype RC while considering development stages and environmental conditions. Nested model comparisons via AIC indicated that 17 genotypes varied consistently in at least one parameter over six different models compared to RC (Figure 5). Three genotypes consistently varied in two parameters, with founders of NAM33 and 37 having lower *A*_qI_ and maxNPQ compared to RC, while the NAM54 founder had slower relaxation of *A*_qM_ which was associated with a larger *τ*_qM_, and a higher maximum NPQ (Figure 5).

### Modeling impact of NPQ variation on canopy photosynthesis

**Figure 6:**
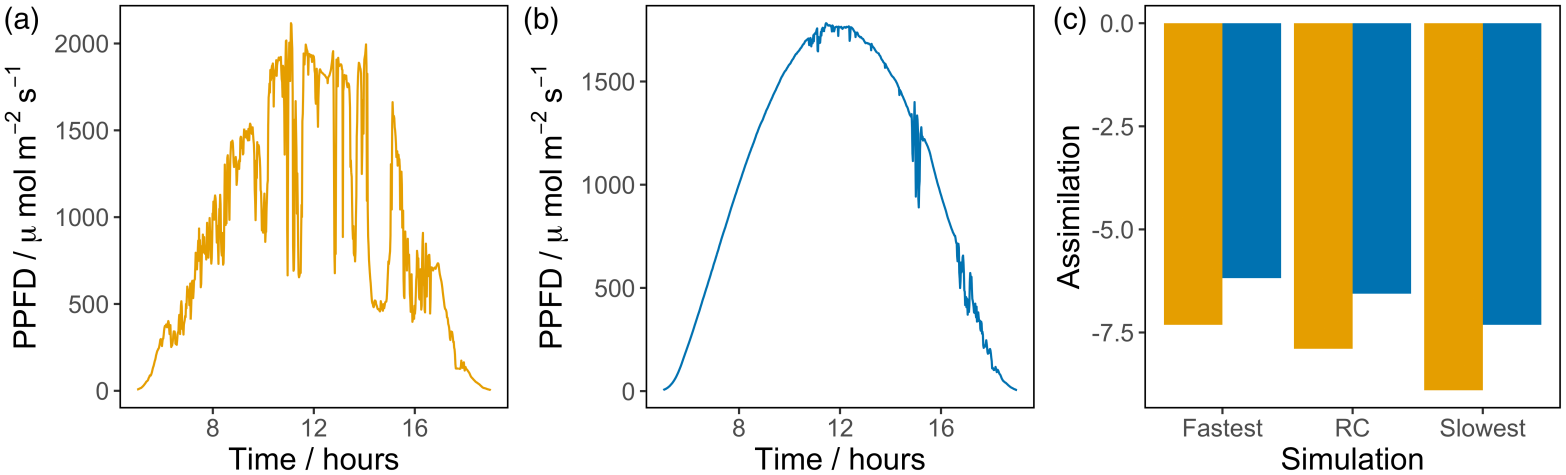
Canopy simulations comparing the impact of SoyNAM phenotypic variability in non‐photochemical quenching (NPQ) relaxation kinetics on carbon assimilation on an intermittently cloudy day and sunny day. Measured light intensity on an intermittently cloudy day 266 (a) and sunny day 227 (b) in Illinois in 2021. (c) Illustrated are losses in Ac resulting from the measured rates of *τ*_qE_ and *τ*_qM_ comparing the slowest, fastest, mean, and RC values from the SoyNAM population on a cloudy (orange bars) and sunny (blue bars) day.

A canopy model was used to calculate the impact of varying NPQ parameters on photosynthetic efficiency, using the phenotypic diversity present in the SoyNAM founders. The minimum and maximum recorded genotypic values for NPQ relaxation (*τ*_qE_ and *τ*_qM_) were used to estimate the potential decrease in CO_2_ assimilation caused by slow relaxation of NPQ on a cloudy (August 14th) and sunny (August 15th) day in Illinois in 2021 (Figure 6a,b). Simulations estimate the difference in CO_2_ assimilation between canopies with the fastest and slowest relaxing NPQ kinetics observed would equate to 1.6% on intermittently cloudy days, and 1.1% on a sunny day (Figure 6c).

To assess whether variation in NPQ relaxation could impact yield, a correlation analysis was performed comparing the phenotypic data collected here with published yield estimates for SoyNAM founders (Diers et al., 2018). When combining the 2 years, a significant positive correlation (*P* < 0.05) was observed between *A*_qI_ and yield, however, there was no association with other parameters including (*τ*_qE_ and *τ*_qM_) (Figure S1). When comparing years separately, the same trend is observed (Figure S1).

## DISCUSSION

We identified genotypic and environmental effects on the induction and relaxation of NPQ that persisted through overnight dark incubation (Figures 1, 3, and 4; Figure S8). The role of the environment was further supported by variation in maximum inducible NPQ measured on samples taken from the same plot over consecutive days (Figure S23). The mechanism behind environmental effects is unclear, but it is possible that measured NPQ kinetics were influenced by changes in gene expression or the status of xanthophyll cycle pigments in the leaf.

Long‐term (*A*_qI_) and intermediate (*A*_qM_) relaxing NPQ were the parameters best explained by the environment. There was a strong correlation between measured *A*_qI_ values for each genotype in 2021 and 2022 (*R* ^2^ = 0.6, *P* < 0.05) (Figure S1), but *A*_qM_ was the parameter for which there was the lowest correlation between years (*R* ^2^ = 0.099, *P* = 0.54) (Figure S1). The environment had a consistent impact on *A*_qI_ (Figure 3), while a much stronger influence over *A*_qM_ in 2022. The differential impact of the environment on variation in *A*_qM_ between years may in part explain the lack of correlation in these values measured for genotypes between 2021 and 2022 (Figure S1).

The vapor pressure deficit had the biggest impact on NPQ (Figure 3). High VPD typically causes plants to close stomata to reduce water loss (Grossiord et al., 2020), leading to lower internal carbon concentrations, and reduced NPQ. Accordingly, we saw a consistent positive correlation between VPD and maximum NPQ and *A*_qE_ (Figure 3). A portion of qE is known to be dependent on the presence of Zeaxanthin (Zx) (Holt et al., 2005). This pigment plays an important role in modulating NPQ and is created from the de‐epoxidation of violaxanthin (Vx) via the intermediate antheraxanthin (Ax) (Jahns & Holzwarth, 2012). High Zx levels decelerate the relaxation of qE (Niyogi et al., 1998), and the rate of conversion of Zx to Vx appears to be associated with slower relaxing phases of NPQ, referred to as qZ (described by the variable qM in this manuscript) (Kress & Jahns, 2017; Nilkens et al., 2010). The amount of Zx is dependent on the combined xanthophyll pool size (VAZ), which is regulated by carotene hydroxylase (Davison et al., 2002), and the steady state de‐epoxidation state of xanthophyll pigments, which is controlled by the activity of zeaxanthin epoxidase (ZEP) and violaxanthin de‐epoxidase (VDE). Overexpression of ZEP and VDE in Arabidopsis suggests that the abundance of ZEP is critical in controlling the rate of interconversion between Vx and Zx while altering the level of VDE has only a marginal impact (Küster et al., 2023). These findings suggest further characterization of ZEP activity in field‐grown soybean could provide insight into environmental effects on NPQ kinetics.

Regulation of xanthophyll cycle enzymes in response to environmental stress appears to vary between tissues, species, and cultivars (Bethmann et al., 2019; Grieco et al., 2020; Schwarz et al., 2015). For example, during drought stress, ZEP was degraded in leaves but accumulated in the roots of Arabidopsis (Schwarz et al., 2015). Whereas ZEP was shown to increase in abundance of leaves from an Iranian cultivar of wheat exposed to water stress, but not one from the United Kingdom, while VDE remained stable (Grieco et al., 2020). Interestingly, the wheat plants with increased ZEP were characterized by a higher qZ amplitude (Zx‐dependent quenching on a 10–30 min time scale) and a larger constant for relaxation (*τ*_qZ_) relative to control, which would have been expected if ZEP decreased. This led the authors to hypothesize that the slowdown in NPQ was caused by a change in enzyme activity rather than in stoichiometry (Grieco et al., 2020). While we do not have the necessary data to determine whether the soybean in our study experienced water stress, the positive association between A_qE_ and VPD (Figure 3), and the decline in *τ*_qM_ (which would be the parameter the most related to *τ*_qZ_) following a precipitation event in 2022 (Figure 4), would be consistent with increased ZEP activity. However, further investigation is required to determine if xanthophyll pigment content and enzyme abundance can account for the observed impact of other environmental variables on NPQ relaxation.

Between genotypes there was a small number of lines that varied compared to the reference with respect to parameters linked to ZEP (Figure 5). A single founder had lower *A*_qE_, or slower relaxation of qE (*τ*_qE_), while six had slower relaxation of qM (*τ*_qE_) and make candidates for possessing altered rates of conversion between Zx to Vx (Figure 5). However, we did not identify significant (*P* < 0.05) G × E associations (Table 2), indicating that all genotypes respond similarly to the environment. Fast conformational change in the thylakoid membrane is also known to play an important role in NPQ and its components (Ruban et al., 2012; Sacharz et al., 2017; Schaller et al., 2010). The protein PsbS has been proposed to be important for this phenomenon, by facilitating thylakoid membrane re‐organization through modulating the interaction between light‐harvesting complex II (LHCII) and PSII (Kiss et al., 2008). The formation of qE is associated with the dissociation of LHCII complexes from PSII and reversible aggregation in the thylakoid membrane (Johnson et al., 2011). PsbS drives LHCII clustering by causing a hydrophobic mismatch between protein and lipid domains in the thylakoid, leading to membrane thinning (Wilson et al., 2024) and conversion of LHCII from a light‐harvesting, to an energy‐quenching state (Ruan et al., 2023). A study conducted on a PsbS knock‐out rice mutant revealed that *τ*_qE_ depended on the level of phosphorylation of Lhcb1 and Lhcb2 (Pashayeva et al., 2021). In addition, high temperature has been shown to lead to an increase in the thylakoid membrane stiffness, causing a decline in the amplitude of the long‐lifetime components of fluorescence decay without affecting its lifetime (Pollastri et al., 2019). While it is difficult in our study to isolate the effect of air temperature on NPQ relaxation from the effect of other environmental variables, our results showed an important impact of temperature on NPQ relaxation and its components (Figure 3; Figure S8), though its effect varied depending on the year and the components. How multiple environmental stressors may interact and affect NPQ relaxation remains understudied.

Our study also revealed a seasonal pattern in NPQ relaxation (Figure 4) and the importance of aggregated environmental variables (Figure S8), suggesting a lasting effect on the environment. Long‐term quenching (qI) was the variable best explained by the environment (Figure 3). Photoinhibitory quenching has been shown to operate at a seasonal timescale (Demmig‐Adams & Adams, 2006) and could have a sustained impact on NPQ relaxation. The evergreen shrub *Jatropha curcas* induces long‐term NPQ during drought, which is associated with increased VAZ pool size and photoinhibition (Sapeta et al., 2023). The latter mechanism has been shown to be linked to the controlled turnover of PSII to slow electron transport and prevent damage to photosystem I (Tikkanen et al., 2014). Long‐term adaptation of NPQ has also been observed in *Taxus baccata* with needles exposed to high light in winter showing a slowed NPQ relaxation weeks after (Robakowski & Wyka, 2009). Elsewhere, studies with Arabidopsis identified long‐lasting NPQ termed “qH” which is active under cold or heat stress (Brooks et al., 2013; Malnoë et al., 2018). Still, more studies are needed to understand how NPQ relaxation is affected by its environment on a longer timescale in crops and the underlying mechanisms contributing to qI.

The SoyNAM population is known to possess genotypic diversity with respect to photosynthetic variables: there is a large variation in rates of rubisco activation, which was reported to cause a >5‐fold difference in carbon fixation during the first 5 min following the transition between dark and light conditions (Soleh et al., 2017), and loci have been identified influencing carbon assimilation and electron transport (Montes et al., 2022). Intriguingly the founder genotype of NAM12, which has previously been shown to possess the highest levels of steady‐state electron transport (*J*_max_) (Montes et al., 2022), and the slowest rate of rubisco activation (Soleh et al., 2017), was the only genotype that showed significantly slower rates of relaxation of qE relative to the reference (Figure 5). *J*_max_ was measured by Montes et al. under saturating light conditions when NPQ is maximal, so would be expected to be independent of NPQ relaxation rates (Montes et al., 2022), but further analysis of RIL population will be required to determine if these traits are linked.

A known variant is found at the *e2* locus, which encodes a homolog of the Arabidopsis circadian clock gene GIGANTEA (Watanabe et al., 2011). This gene has been shown to have pleiotropic effects which influence soybean photosynthesis (Montes et al., 2022), canopy coverage (Xavier et al., 2017), and yield traits (Diers et al., 2018). In soybean, the recessive e2 allele contains a premature stop‐codon and promotes early flowering (Watanabe et al., 2011), while the functional late maturity *E2* allele is associated with greater yield, taller plants and smaller seed mass (Diers et al., 2018), in addition to increased canopy coverage (Xavier et al., 2017) and higher rates of maximum electron transport (Montes et al., 2022). The *E2* allele is segregating in seven of the NAM founders, three of which (NAM33, 37, and 50) were identified as having significantly less *A*_qI_ compared to the reference line. This would be in accordance with predictions that reducing *A*_qI_ can improve photosynthesis and yield, with the rationale that lower *A*_qI_ will increase the efficiency of photosynthesis during shadeflecks leading to greater carbon assimilation (Zhu et al., 2004), but contrasts with the negative correlation between yield and *A*_qI_ observed here.

Although a small number of lines were found to vary in NPQ kinetics compared to RC (Figure 5), the overall diversity in NPQ relaxation parameters between SoyNAM founders was limited, for example, only a 1.5‐fold change between the smallest and highest *τ*_qM_ was observed (Table 2). This small amount of variation is not a general phenomenon related to NPQ, with several studies finding substantial variation in species ranging from Arabidopsis to Maize (Cowling et al., 2022; Jung & Niyogi, 2009; Rungrat et al., 2019; Sahay et al., 2023). For example, analysis of rice genotypes found a large variation in maximum NPQ (Kasajima et al., 2011), which was attributed to an insertion in the promoter region of photosystem II subunit S (PsbS) resulting in higher expression and higher NPQ in japonica rice (Wang et al., 2017). In the case of soybean, this lack of variation may reflect an unusual number of genetic bottlenecks in domestication and then introduction into the United States (Hyten et al., 2006), with only 28 ancestors contributing to 95% of the genes in cultivars released between 1947 and 1988 (Gizlice et al., 1994). The lines used in this study were chosen for their diversity, but it remains unclear if there is more variation in NPQ to be found in the wider soybean germplasm and whether more may be found in collections of the wild ancestor *Glycine soja* which is considered a largely untapped source of genetic variation in cultivated soybean (Kofsky et al., 2018).

Previous transgenic manipulations suggested that speeding up the relaxation of NPQ can increase photosynthetic efficiency leading to improved growth (Kromdijk et al., 2016) or seed production (De Souza et al., 2022), although it is not always the case (Garcia‐Molina & Leister, 2020; Lehretz et al., 2022). The reason for this is not clear, but the interplay between total photoprotection, the ability to use assimilate, and the rates of activation and relaxation are likely to be important. Wang et al. (2020) used a ray tracing algorithm and canopy model to estimate the predicted benefit of manipulating NPQ or photosynthetic induction rates in soybean given available natural variation. The data used were based on preliminary measurements made on the same SoyNAM panel as in this publication (Diers et al., 2018; Song et al., 2017). However, predictions of the impact of altering NPQ relaxation were based on light‐to‐dark measurements, which are unlikely to be realistic for a field environment and did not focus on identifying which soybean genotypes could be beneficial for genomic selection from the perspective of NPQ relaxation. Here, the differences in NPQ parameters between lines were smaller, approximately 28% difference in *τ*_qE_ between fastest and slowest relaxing lines as compared to 40% (Wang et al., 2020), which equates to 1.6% daily assimilation, likely due to the much deeper sampling and measurement conditions.

Crop yield is a quantitative trait influenced by a host of factors, from pathogen resistance to nutrient use efficiency (Burgess et al., 2023). The relationship between photosynthesis and yield is debated and complex, with both positive and negative correlations reported depending on the crop and parameter (as reviewed by Theeuwen et al., 2022). Analysis of the response of soybean to an artificial increase of photosynthesis in the field by elevation of [CO_2_], shows that while older cultivars appear sink limited, modern cultivars appear to be source limited and can fully use any increase in photosynthesis (Ainsworth & Long, 2021). While our data do not support a relationship between yield and the rates of NPQ relaxation (*τ*_qE_ and *τ*_qM_) (Figure S6), this does not necessarily preclude a relationship. Many traits which affect yield vary between SoyNAM founders, including characteristics such as canopy architecture and steady‐state assimilation rates (Montes et al., 2022), it is therefore conceivable that variation in parameters with a larger influence on yield could obscure a relationship with NPQ relaxation. An additional consideration is the interplay between NPQ and ABA signaling mediated via the xanthophyll cycle. ZEP catalyzes the first committed step of ABA synthesis, and plants with elevated ZEP expression have been reported to have higher ABA levels (Grieco et al., 2020; Park et al., 2008). ABA can influence a range of plant phenotypes affecting yield, from growth and development to carbon partitioning and grain number (Kavi Kishor et al., 2022). Identifying the mechanistic basis of the variation in NPQ parameters was beyond the scope of this study, but if it is subsequently found to be the result of changes in ZEP abundance, it could be difficult to identify the precise cause of changes in yield.

In summary, the SoyNAM founders display a narrow range of variation in NPQ kinetics. Environmental variables, such as VPD, impacted NPQ kinetics, and all genotypes responded similarly. Repeat sampling allowed the identification of genotypes with significantly smaller or larger values compared to the common parental line. However, given the limited diversity of NPQ relaxation rates in soybean germplasm, achieving increases in photosynthetic efficiency through manipulating NPQ is likely to be best achieved through transgenic approaches. Due to the potential complexity of relationships between components of NPQ and yield, further experiments are required to assess whether strategies to alter NPQ will be beneficial for crop productivity.

## EXPERIMENTAL PROCEDURES

### Plants and growth conditions

The 41 parents of the Soybean Nested Association Mapping (NAM) population (Diers et al., 2018; Song et al., 2017) were grown in the field at the Crop Sciences Research and Education Center at the University of Illinois at Urbana‐Champaign in 2021 (latitude 40.084604 and longitude −88.227952) and 2022 (latitude 40.064866 and longitude −88.193084). Seeds were planted in 1.2 m single‐row plots with a 0.75 m row spacing, with 40 seed m^−1^ in a North–South orientation on June 5, 2021 and June 13, 2022 (Table S8). The experiment was arranged in a randomized complete block design, with five replicate plots per genotype. The field was rainfed, and standard agronomic practices were employed.

### Meteorological data collection

Meteorological variables were measured every 30 min by a weather station at the University of Illinois Energy Farm, approximately 1 km from the Crop Sciences Research and Education Center (latitude 40.062832 and longitude −88.198417). Air temperature (*T*_a_, °C) and relative humidity (RH, %) were recorded by a HMP45C probe (Campbell Scientific, Logan, UT, USA), and incoming shortwave radiation (*F*_sd_, W m^−2^) was from a CNR1 radiometer (Kipp & Zonen, The Netherlands), both instruments were installed 4 m above the grounds. Precipitation data was obtained from the Illinois State Water Survey (Illinois State Water Survey, 2022). *T*_a_ and RH were used to calculate saturation vapor pressure (*e*_s_) and actual vapor pressure (*e*_a_) for each 30 min period, which were then used to calculate vapor pressure deficit (VPD, kPa) as per Equations (1), (2), (3):

(1) $$e_{s}=0.6106\times \exp^{\left(\frac{\left(17.27\times T_{a}\right)}{\left(237.3+T_{a}\right)}\right)}$$

(2) $$e_{a}=\frac{\mathrm{RH}\times e_{s}}{100}$$

(3) $$\mathrm{VPD}=e_{s}-e_{a}$$

Occasional gaps in meteorological data are inevitable when measuring at these time scales, so data gaps were filled where needed, whereby an artificial neural network was used to generate a complete time series with external data sourced from the University of Illinois Willard Airport weather station (station ID: 725315‐94870) and ERA‐interim data from the European Centre for Medium Range Forecasts. In total, less than 5% of data required gap filling. Daily summary values were then calculated for each variable, with *T*_a_, VPD, and *F*_sd_ representing the value at 10 a.m., and rainfall as a sum up until 10 a.m. The variables T_a__3day, VPD_3day, and Fsd_3day represent the average for the past 3 days for these values, whereas the variable Precip_3day represents the sum in precipitation for the past 3 days.

### Chlorophyll fluorescence analysis

For field experiments, plants were sampled between 8:00 and 10:00 at seven time points across development in 2021 and six time points in 2022 (Table S3) according to Gotarkar et al. (2022). Briefly, five 4.8 mm leaf disks were collected per plot, sampling from the upper‐most mature leaf using a cork borer (H9663; Humboldt Mfg. Co., Elign, IL, USA). Leaf disks were then transferred face down into wells of a flat‐bottomed 96 well plate (FB012929; Fisher Scientific, Waltham, MA, USA), humidity was maintained by placing a half‐wet nasal aspirator filter into each well (iHank‐Nose B07P6XCTGV; Amazon, Seattle, WA, USA), and plates were sealed and wrapped in aluminum foil followed by overnight dark adaptation. Measurements were taken with a modulated chlorophyll fluorescence imaging system (CF imager; Technologica, Colchester, UK). To induce low–high–low light fluctuations, samples were illuminated for 10 min at 50 μmol m^−2^ sec^−1^, followed by 15 min at 2000 μmol m^−2^ sec^−1^ and 50 min at 50 μmol m^−2^ sec^−1^. *F*_m_, was determined by applying saturating pulses (4000 μmol m^−2^ sec^−1^) at 2.5, 5, 7.5, and 10 min after the actinic light was turned on (50 μmol m^−2^ sec^−1^), 2.5, 5, 7.5, 10, 12.5, and 15 min after high light exposure (2000 μmol m^−2^ sec^−1^), and 2.5, 5, 10, 15, 20, 25, 30, 35, 40, 45, and 50 min following return to low light (50 μmol m^−2^ sec^−1^). The background was excluded manually and NPQ values at each pulse were calculated.

NPQ values were calculated for each time point using custom MatLab scripts according to Gotarkar et al. (2022). NPQ relaxation parameters *A*_qE_, *A*_qM_, *A*_qI_, *τ*_qE_, and τ_qM_ were then calculated by fitting the sum of a double exponential function to measured NPQ values following shut off of the actinic light, according to Equation (4) (Dall'Osto et al., 2014):

(4) $$\mathrm{NPQ}=A_{\mathrm{qI}}+A_{\mathrm{qE}}^{\left(-\frac{t}{\tau_{\mathrm{qE}}}\right)}+A_{\mathrm{qM}}^{\left(-\frac{t}{\tau_{\mathrm{qM}}}\right)}$$

using the fit function in MatLab R0218b, where *t* is the measured fluorescence at a given time point. Maximum NPQ values are defined as the maximum value reached during the 15 min illumination at high light.

### 3D soybean model and light distribution

The dynamics of lighting within a soybean canopy were predicted with a 3D architectural representation, using our previously presented soybean canopy model (Song et al., 2020; Wang et al., 2020). The model was parameterized on the measured architecture of the soybean hub parent IA3023 (RC) at the University of Illinois Energy Farms in August 2022 (measured canopy parameters are listed in Tables S9 and S11). Leaf area was measured when the soybean plants were on August 18th, 2021. The youngest mature leaf (approximately third trifoliate from the top) was selected for analysis, and the area of all three leaves in the trifoliate from three plots was measured with a leaf area meter (LI3100C; LI‐COR Environmental, Lincoln, NE, USA) (Table S9). Detailed parameters were measured for five genotypes on August 19th, 2021 and eight genotypes on August 30th, 2022. Plant height was measured from the base to the tip after the plants were cut from the base and stretched. Leaf width was measured at the widest point from each leaflet in a trifoliate and averaged. Leaf length was measured from base to tip for each trifoliate and averaged (Table S10). Internode length was measured for the sixth internode from the top, with one value recorded per plant, and branch angle was measured for the sixth branch from the top using a digital protractor, with one measurement per plant. The total number of trifoliate, number of primary and secondary branches, and number of pods per plant were counted manually for one plant per plot (Table S10). Leaf Area Index (LAI) was measured using the SunScan canopy analysis system (SS1; Delta‐T Devices Ltd, Cambridge, UK) with a 1 m probe according to the manufacturer's instructions. LAI was measured at the R5 developmental stage between 11:56 and 13:50 on August 20th, 2021 on both sides of each plot and averaged, with the probe positioned parallel to the rows (Table S11). To calculate the actual light environment of the soybean canopy, measured PAR data on DOY 226 and 227 of 2021 in Bondville, IL. (Earth System Research Laboratory, Global Monitoring Division, https://www.esrl.noaa.gov/gmd/grad/surfrad/dataplot.html) was incorporated (Table S12). A forward ray‐tracing algorithm (FastTracer; Song et al., 2013) was used to predict the light absorption of each leaf pixel (ca. 5 mm^2^) every 1 min from 05:00 to 19:00 in Champaign IL, US (40.11 N, 88.21 W).

### Simulation of dynamic photosynthesis

Dynamic photosynthetic rates were calculated for every 10 sec (Δ*t*) of the day using the absorbed light for each leaf pixel, considering rates of Rubisco activation and NPQ relaxation (Wang et al., 2020). Then the canopy net CO_2_ uptake (*A*_c_) was calculated as

$$A_{c}\left(t\right)=\frac{\sum \left(A_{i}\left(t\right)\cdot S_{i}\right)}{S_{\text{ground}}}$$

where *A*_*i*_(*t*) is the CO_2_ uptake rate of a leaf pixel; *S*_*i*_ is the surface area of each pixel, and *S*_ground_ represents the occupied ground area of the simulated canopy. All simulations were conducted in MATLAB 2021a (The Mathworks, Inc®).

Time constants of NPQ relaxation and Rubisco activation across the NAM population were measured and used as input for the dynamic photosynthetic model (model inputs were listed in Table S13). The time constant of Rubisco de‐activation was assumed to be double the time required for activation for each genotype (Taylor & Long, 2017).

### Statistical analysis

Technical replicates from the chlorophyll fluorescence analysis were averaged prior to statistical analyses. The impact of genotypes, the environment, and their interaction on NPQ relaxation was assessed by fitting an anova model in R v4.1.2 (R Core Team, 2016) and RStudio v2024.04.0 (RStudio Team, 2015) using the stats R package v4.1.2. The anova model was written as NPQ parameter ~ genotype (G) + day (or the environment, E) + genotype * day with G and E set as fixed factors. The impact of the environment was assessed for each NPQ relaxation parameter using a stepwise regression model to determine which environmental variables had the strongest impact on the parameters. The initial linear model included all the environmental variables. A both‐direction stepwise algorithm then tested the model by removing and re‐including environmental variables one by one. The AIC was used to select the best minimum adequate model (lowest AIC) using the stats R package v4.1.2. This test was performed with data collected in 2021, 2022, and for the 2 years together. Centered and scaled environmental variables were used as input in the model. The relationships between genotypes, the environment, and NPQ relaxation were explored using CCA. This multivariate statistical approach identifies linear combinations of variables for the two datasets (here, NPQ and environmental data) to construct a pair of canonical variates that are maximally correlated with each other on the first canonical axis (CC1). Then, a second pair of canonical variates is made with a maximized correlation on the second canonical axis (CC2) but uncorrelated with CC1. The process is repeated for each canonical variates' pairs. A significant correlation between pairs enables associations among the different variables. The CCA analysis was performed in R using the CCA R package v1.2.1 (González & Déjean, 2021). The statistical significance of canonical correlation coefficients was carried out using Wilks' Lambda with the CCP R package v1.2 (Menzel, 2022). All NPQ relaxation parameters were used as input for the first dataset. Environmental variables were used as input for the second dataset with the exception of morning_Ta to reduce co‐linearity due to its high correlation with Ta_3day.

For chlorophyll fluorescence analysis, leaf disks with *F*_v_/*F*_m_ value <0.75 were excluded to ensure measured values were from healthy disks representative of whole plant kinetics. Additional outliers were defined and removed if calculated parameter values were <0. The effect of genotypic differences from the RC for each NPQ relaxation parameter was estimated using linear mixed‐effects models (Bates et al., 2015). Linear fixed effects included days, air temperature (*T*_a_), VPD, precipitation (Precip), incoming shortwave radiation (*F*_sd_), a 3‐day rolling mean air temperature (Ta 3 day), and a 3‐day rolling mean vapor pressure deficit (VPD 3 day), with random effects for plot and leaf disks (Data S1). AIC was used to compare linear mixed‐effects models (Akaike, 1974; Faraway, 2016). Briefly, nested model comparisons via AIC determined which genotypes exhibit different measures from the baseline genotype RC, following a procedure to fit a smaller model with one genotype removed. This smaller model assumes that there is no difference between that genotype and the RC baseline. The computed AIC value for this smaller model was compared with the AIC value of the full model with all genotypes considered. If the smaller model has a lower AIC value, then the removed genotype does not exhibit an effect on NPQ relaxation parameters that is different from the RC baseline. This procedure was repeated for all genotypes and all linear mixed‐effects models involving NPQ relaxation parameters as a response (Data S1). In addition to this approach, a PCA was performed for each days separately to identify any patterns among NAM groups (i, e, diverse, elite, PI, and RC) and/or notable genotypes. The analysis was performed using the ade4 (v1.7‐22) R package (Dray & Dufour, 2007). Centered and scaled NPQ parameters were used as input variables. Differences between NAM groups in their principal component coordinates were assessed using a one‐way anova with groups as a fixed factor with the R stats package (v4.1.2).

## CONFLICT OF INTEREST

The authors report no conflicts of interest.

## Supporting information


**Data S1.** Description of statistical analysis comparing NPQ relaxation kinetics between genotypes.

**Data S2.** Supplementary Methods.

**Figure S1.** Comparison of genotypic means for NPQ relaxation parameters measured in 2021 and 2022 using the SoyNAM founders. Scatterplots comparing values of (a) maximum inducible NPQ, (b) *A*_qE_, (c) *A*_qM_, (d) *A*_qI_, (e) *τ*_qE_, and (f) *τ*_qM_. Values represent the mean of seasonal measurements. Pearson correlation coefficient (*R*) and *P*‐value are reported for each parameter.
**Figure S2.** Direct measurement of NPQ in NAM population founders grown in the field (July 23, 2024). (a) Comparison of rates of linear electron flow (LEF_amb_) against ambient PAR (PAR_amb_). (b) Rates of LEF (LEF_high_) following 10 sec illumination at high light, compared to ambient PAR. (c) The difference between rates of LEF under ambient and high light, compared to ambient PAR. (d) Comparison of phiPSII measured under ambient and high light (yellow symbols), versus ambient PAR (gray symbols).
**Figure S3.** Direct measurement of NPQ in NAM population founders grown in the field (July 30, 2024). (a) Comparison of rates of linear electron flow (LEF_amb_) against ambient PAR (PAR_amb_). (b) Rates of LEF (LEF_high_) following 10 sec illumination at high light, compared to ambient PAR. (c) The difference between rates of LEF under ambient and high light, compared to ambient PAR. (d) Comparison of phiPSII measured under ambient and high light (yellow symbols), versus ambient PAR (gray symbols).
**Figure S4.** Comparison of NPQt values for the SoyNAM founders on July 23, 2024. (a) Boxplot comparing NPQt values recorded for SoyNAM founders under high light. Values represent the mean of three technical (individual plant) replicates per plot (*n* = 5). (b) Comparison of NPQt measured under ambient light (PAR_amb_) and NPQt (NPQt_amb_), individual technical replicates are shown. (c) Comparison of NPQt measured under high light (NPQt_high_) and ambient PAR. Individual technical replicates are shown.
**Figure S5.** Comparison of NPQt values for the SoyNAM founders on July 30, 2024. (a) Boxplot comparing NPQt values recorded for SoyNAM founders under high light. Values represent the mean of three technical (individual plant) replicates per plot (*n* = 5). (b) Comparison of NPQt measured under ambient light (PAR_amb_) and NPQt (NPQt_amb_), individual technical replicates are shown. (c) Comparison of NPQt measured under high light (NPQt_high_) and ambient PAR. Individual technical replicates are shown.
**Figure S6.** Pearson correlation coefficients comparing the relationship between genotypic means of NPQ relaxation parameters and EBLUP values for yield calculated by (Diers et al., 2018). Showing correlations for (a) 2021, (b) 2022, and (c) combined 2021 and 2022 data. *R* ^2^ values for significant associations (*P* < 0.05) are displayed.
**Figure S7.** Pearson correlation coefficients comparing the relationship between measured environmental variables. Showing correlations for (a) 2021, (b) 2022, and (c) combined 2021 and 2022 data. *R* ^2^ values for significant associations (*P* < 0.1) are displayed.
**Figure S8.** Coefficients for the best minimum adequate model (lowest AIC) for each NPQ relaxation parameter in 2021 and 2022 combined. The *R* ^2^ and *P*‐value are shown for each model.
**Figure S9.** PCA of NPQ relaxation in the NAM population on June 24, 2021. Diverse, elite, and PI lines are shown in gray, orange, and blue, respectively. Coordinates for replicate measurements on the same line were averaged. RC line is shown in black. Error bars represent the standard deviation. Lines with a *z*‐score >2.5 for one of their components were labeled.
**Figure S10.** PCA of NPQ relaxation in the NAM population on 6/30/21. Diverse, elite, and PI lines are shown in gray, orange, and blue, respectively. Coordinates for replicate measurements on the same line were averaged. RC line is shown in black. Error bars represent the standard deviation. Lines with a *z*‐score >2.5 for one of their components were labeled.
**Figure S11.** PCA of NPQ relaxation in the NAM population on July 7, 2021. Diverse, elite, and PI lines are shown in gray, orange, and blue, respectively. Coordinates for replicate measurements on the same line were averaged. RC line is shown in black. Error bars represent the standard deviation. Lines with a *z*‐score >2.5 for one of their components were labeled.
**Figure S12.** PCA of NPQ relaxation in the NAM population on July 13, 2021. Diverse, elite, and PI lines are shown in gray, orange, and blue, respectively. Coordinates for replicate measurements on the same line were averaged. RC line is shown in black. Error bars represent the standard deviation. Lines with a *z*‐score >2.5 for one of their components were labeled.
**Figure S13.** PCA of NPQ relaxation in the NAM population on July 20, 2021. Diverse, elite, and PI lines are shown in gray, orange, and blue, respectively. Coordinates for replicate measurements on the same line were averaged. RC line is shown in black. Error bars represent the standard deviation.
**Figure S14.** PCA of NPQ relaxation in the NAM population on July 28, 2021. Diverse, elite, and PI lines are shown in gray, orange, and blue, respectively. Coordinates for replicate measurements on the same line were averaged. RC line is shown in black. Error bars represent the standard deviation.
**Figure S15.** PCA of NPQ relaxation in the NAM population on August 11, 2021. Diverse, elite, and PI lines are shown in gray, orange, and blue, respectively. Coordinates for replicate measurements on the same line were averaged. RC line is shown in black. Error bars represent the standard deviation.
**Figure S16.** PCA of NPQ relaxation in the NAM population on July 6, 2022. Diverse, elite, and PI lines are shown in gray, orange, and blue, respectively. Coordinates for replicate measurements on the same line were averaged. RC line is shown in black. Error bars represent the standard deviation.
**Figure S17.** PCA of NPQ relaxation in the NAM population on July 12, 2022. Diverse, elite, and PI lines are shown in gray, orange, and blue, respectively. Coordinates for replicate measurements on the same line were averaged. RC line is shown in black. Error bars represent the standard deviation.
**Figure S18.** PCA of NPQ relaxation in the NAM population on July 19, 2022. Diverse, elite, and PI lines are shown in gray, orange, and blue, respectively. Coordinates for replicate measurements on the same line were averaged. RC line is shown in black. Error bars represent the standard deviation.
**Figure S19.** PCA of NPQ relaxation in the NAM population on July 26, 2022. Diverse, elite, and PI lines are shown in gray, orange, and blue, respectively. Coordinates for replicate measurements on the same line were averaged. RC line is shown in black. Error bars represent the standard deviation.
**Figure S20.** PCA of NPQ relaxation in the NAM population on August 4, 2022. Diverse, elite, and PI lines are shown in gray, orange, and blue, respectively. Coordinates for replicate measurements on the same line were averaged. RC line is shown in black. Error bars represent the standard deviation.
**Figure S21.** PCA of NPQ relaxation in the NAM population on August 20, 2022. Diverse, elite, and PI lines are shown in gray, orange, and blue, respectively. Coordinates for replicate measurements on the same line were averaged. RC line is shown in black. Error bars represent the standard deviation.
**Figure S22.** PCA displays the relationships between the NPQ relaxation parameters and the environment in (a–d) 2021 (1296 observations) and (e–h) 2022 (1025 observations). (a, c, e, g) PCA shows the spatial distribution of the different observations on the principal components (PC). The circular shapes represent observations, with different colors representing different days. The triangular shapes show the average of different NAM groups on different days, with diverse, elite, PI, and RC groups shown in gray, orange, blue, and black, respectively. For each group, a solid line connects those points to represent their evolution throughout the season. (b, d, f, h) The correlation circle shows the relationships between variables. Variables related to the NPQ relaxation parameters, and the environments are represented in red and black, respectively. The percentage of total variance explained by the PC1, PC2, and PC3 is shown on the axis title.
**Figure S23.** Comparison of environmental impacts on maximum inducible NPQ values measured for *Glycine max* genotype LD11 by leaf disk assay in 2024. Showing three consecutive days, on two consecutive weeks from 16th and 18th (a) and 22nd to 24th (b) of July. One‐way anova followed by Tukey test was used to determine significant differences between values (*P* < 0.05).

**Table S1.** Raw NPQ time series values for all technical replicates prior to filtering and processing.

**Table S2.** Calculated NPQ relaxation parameters for all plots and genotypes.

**Table S3.** Genotype means for calculated NPQ relaxation parameters combining all data from 2021 and 2022.

**Table S4.** Sampling time points in 2021 and 2022 including days after sowing and development stage.

**Table S5.** CCA table for the year 2021. Loadings of the variables on the canonical dimensions were constructed with the NPQ dataset (columns 1, 2, and 3) and with the environmental dataset (columns 4, 5, and 6). These loadings are correlations between variables and the canonical variates. The last row shows the correlation coefficient between the canonical axis pairs (i.e., the correlation between the two CC1 for the two datasets).

**Table S6.** CA table for the year 2022. Loadings of the variables on the canonical dimensions were constructed with the NPQ dataset (columns 1, 2, and 3) and with the environmental dataset (columns 4, 5, and 6). These loadings are correlations between variables and the canonical variates. The last row shows the canonical correlation coefficient between the canonical axis pairs (i.e., the correlation between the two CC1 for the two datasets).

**Table S7.** *P*‐values obtained from a linear model followed by ANOVA on the principal component (PC) coordinates with the NAM population groups set as a fixed factor (i.e., PC ~ Group).

**Table S8.** Field design with plot numbers and orientation in 2021 and 2022.

**Table S9.** Leaf area measurements for 41 SoyNAM founders were recorded in 2022, data are provided for three biological replicates.

**Table S10.** Canopy measurements for seven SoyNAM founders and reference genotype RC were recorded on August 30th, 2022.

**Table S11.** Leaf area index measurements for 41 SoyNAM founders recorded in 2022, data are provided for two technical and five biological replicates.

**Table S12.** Measured PAR values on the day of the year (DOY) 226 and 227 of 2021 in Bondville, IL. Earth System Research Laboratory, Global Monitoring Division.

**Table S13.** Input values for 3D canopy model.

## Data Availability

All raw NPQ data values prior to filtering and processing are provided in csv format in Table S1. All raw chlorophyll fluorescence imager files and custom scripts can be accessed via FigShare 10.6084/m9.figshare.21574509 and 10.6084/m9.figshare.25939504.
