# Supplementary material for: Variation in relaxation of non‐photochemical quenching between the founder genotypes of the soybean (Glycine max) nested association mapping population
Source: Plant J. 2025 Jan 27;121(2):e17219. doi: 10.1111/tpj.17219 (PMC11771714; doi:10.1111/tpj.17219)
Supplement: Supplementary file 2 — Data S2. Supplementary Methods. [file TPJ-121-0-s002.docx]

**Supplemental Methods**

*Field measurements of NPQt*

The 40 founder genotypes and common parent were planted in single replicate, ~7.5 plant/ft 4 row plots at 30 inch spacing on May 13, 2024 at SoyFACE farm, University of Illinois Urbana-Champaign, Champaign, IL. A Multispeq v2.0 device was used to collect chlorophyll fluorescence parameters between 10:00-16:00 on July 23^rd^ 2024 and July 30^th^ 2024, using the program ‘Rapid PS responses with ECS fast ECS DIRK and NPQt DMK – SJB’ adapted from (Kanazawa *et al.*, 2021). NPQt is calculated by the following equation according to (Tietz *et al.*, 2017):

NPQ(_T_) = 4.88/((*F_m_’*/*F_o_’*) -1) -1

*Measurement of maximum inducible NPQ on consecutive days*

A single 18m row of Glycine max genotype LD11 was planted at the Energy farm, University of Illinois Urbana-Champaign, Urbana, IL on May 30, 2024. Leaf disks were sampled between 8:00-9:30 in the morning of the 17^th^, 18^th^ and 19^th^ and the 22^nd^, 23^rd^, 24^th^ of July, floating on water for transfer back to the laboratory. Leaf disks were then placed in 96 well plates and held in place with a damp sponge. Plates were sealed with parafilm and wrapped in aluminium foil and Chlorophyll fluorescence parameters were measured using Closed FluorCam FC 800-C following overnight dark incubation at room temperature (~24 ^o^C).The following program conditions: 50 µmol m^2^ s^-1^ for 10 min, 1640 µmol m^2^ s^-1^ for 15 min with measuring flashes every 2.5 min, followed by 50 µmol m^2^ s^-1^ light for 50 min measuring flashes 2.5 min x2, 5 min x8.

**References**

**Kanazawa, A., Chattopadhyay, A., Kuhlgert, S., Tuitupou, H., Maiti, T. and Kramer, D.M.** (2021) Light potentials of photosynthetic energy storage in the field: what limits the ability to use or dissipate rapidly increased light energy? *R. Soc. Open Sci.*, **8**, 211102. Available at: https://doi.org/10.1098/rsos.211102 [Accessed August 12, 2024].

**Tietz, S., Hall, C.C., Cruz, J.A. and Kramer, D.M.** (2017) NPQ(T): a chlorophyll fluorescence parameter for rapid estimation and imaging of non-photochemical quenching of excitons in photosystem-II-associated antenna complexes. *Plant Cell Environ.*, **40**, 1243–1255. Available at: https://doi.org/10.1111/pce.12924 [Accessed October 22, 2024].
