# Supplementary figures and images for: Variation in relaxation of non‐photochemical quenching between the founder genotypes of the soybean (Glycine max) nested association mapping population
Source: Plant J. 2025 Jan 27;121(2):e17219. doi: 10.1111/tpj.17219 (PMC11771714; doi:10.1111/tpj.17219)

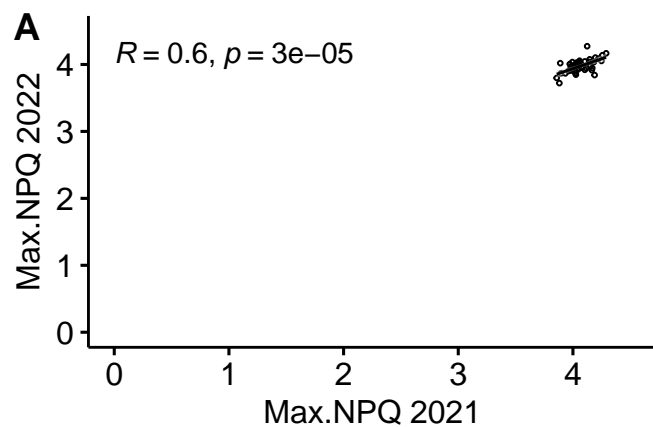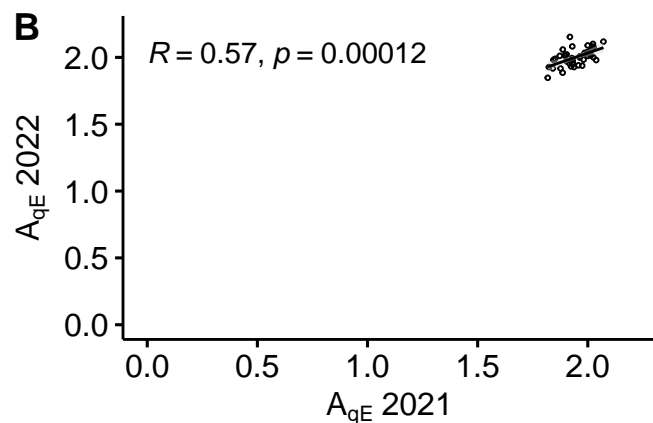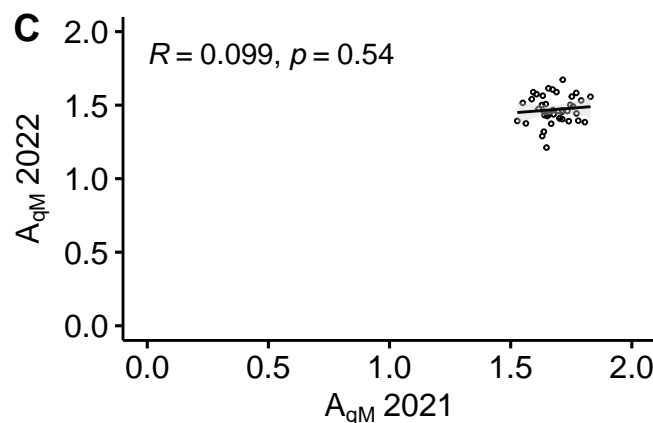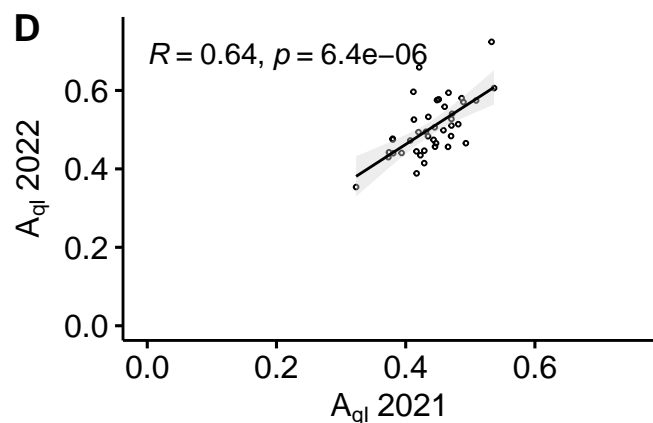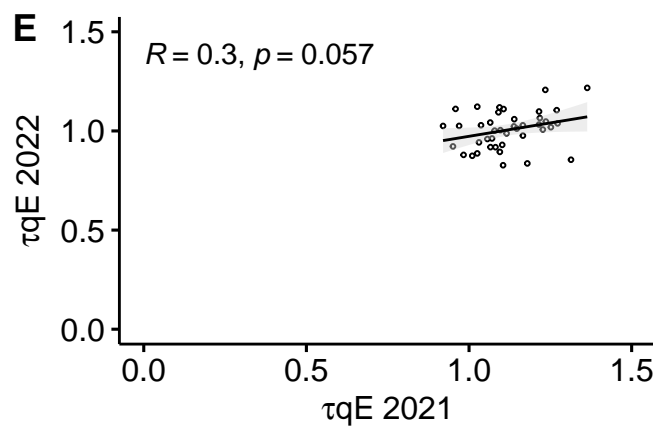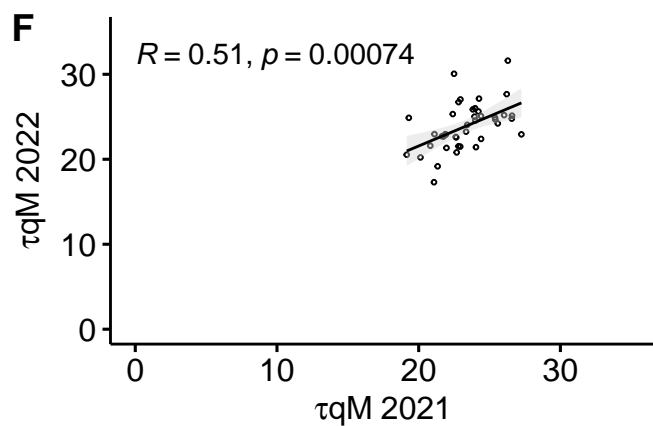

Supplement: Supplementary file 3 — Figure S1. Comparison of genotypic means for NPQ relaxation parameters measured in 2021 and 2022 using the SoyNAM founders. Scatterplots comparing values of (a) maximum inducible NPQ, (b) A qE, (c) A qM, (d) A qI, (e) τ qE, and (f) τ qM. Values represent the mean of seasonal measurements. Pearson correlation coefficient (R) and P‐value are reported for each parameter. Figure S2. Direct measurement of NPQ in NAM population founders grown in the field (July 23, 2024). (a) Comparison of rates of linear electron flow (LEFamb) against ambient PAR (PARamb). (b) Rates of LEF (LEFhigh) following 10 sec illumination at high light, compared to ambient PAR. (c) The difference between rates of LEF under ambient and high light, compared to ambient PAR. (d) Comparison of phiPSII measured under ambient and high light (yellow symbols), versus ambient PAR (gray symbols). Figure S3. Direct measurement of NPQ in NAM population founders grown in the field (July 30, 2024). (a) Comparison of rates of linear electron flow (LEFamb) against ambient PAR (PARamb). (b) Rates of LEF (LEFhigh) following 10 sec illumination at high light, compared to ambient PAR. (c) The difference between rates of LEF under ambient and high light, compared to ambient PAR. (d) Comparison of phiPSII measured under ambient and high light (yellow symbols), versus ambient PAR (gray symbols). Figure S4. Comparison of NPQt values for the SoyNAM founders on July 23, 2024. (a) Boxplot comparing NPQt values recorded for SoyNAM founders under high light. Values represent the mean of three technical (individual plant) replicates per plot (n = 5). (b) Comparison of NPQt measured under ambient light (PARamb) and NPQt (NPQtamb), individual technical replicates are shown. (c) Comparison of NPQt measured under high light (NPQthigh) and ambient PAR. Individual technical replicates are shown. Figure S5. Comparison of NPQt values for the SoyNAM founders on July 30, 2024. (a) Boxplot comparing NPQt values recorded for SoyNAM founder [file TPJ-121-0-s012.zip › Figure_S1_NAM_year_comparison.pdf]

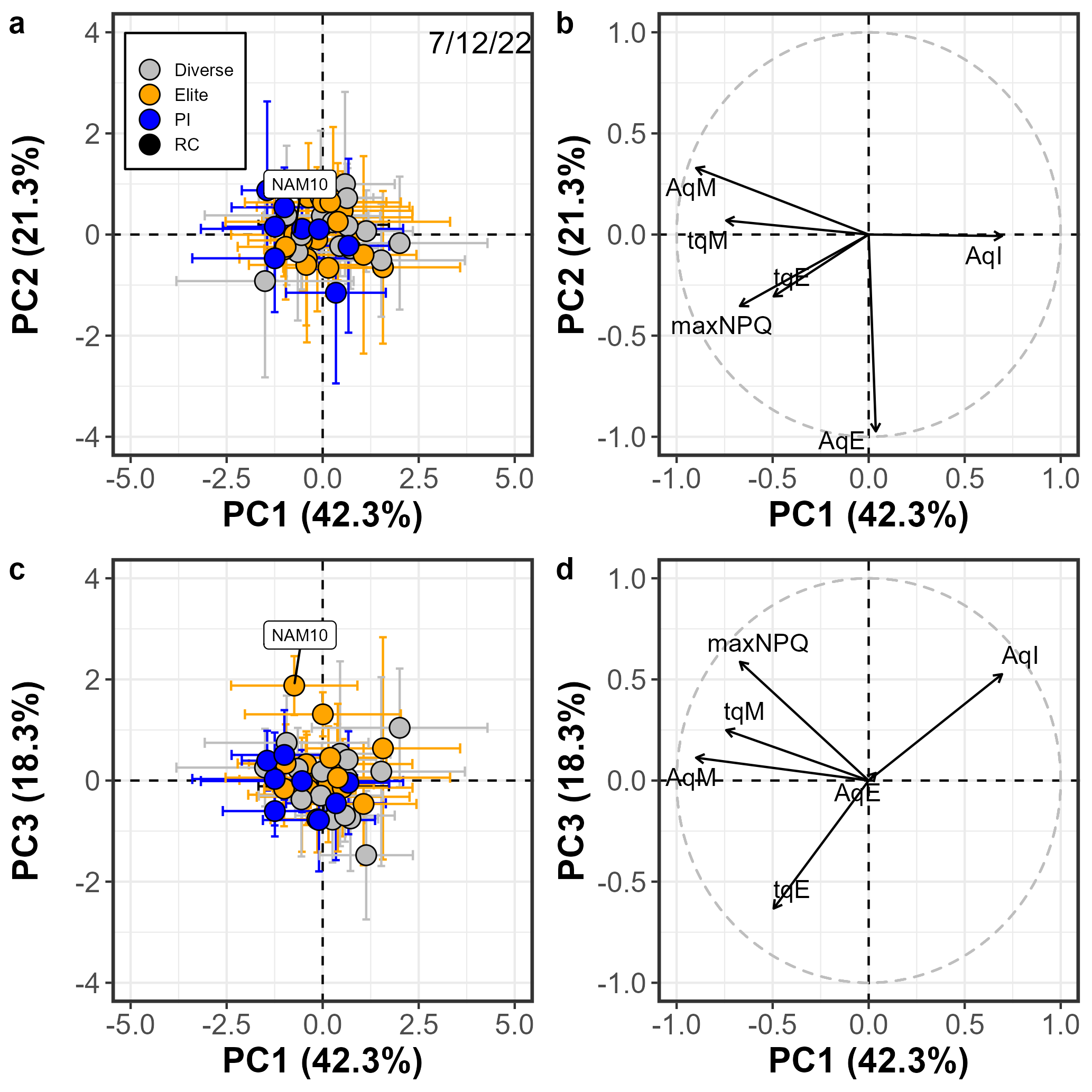

Supplement: Supplementary file 3 — Figure S1. Comparison of genotypic means for NPQ relaxation parameters measured in 2021 and 2022 using the SoyNAM founders. Scatterplots comparing values of (a) maximum inducible NPQ, (b) A qE, (c) A qM, (d) A qI, (e) τ qE, and (f) τ qM. Values represent the mean of seasonal measurements. Pearson correlation coefficient (R) and P‐value are reported for each parameter. Figure S2. Direct measurement of NPQ in NAM population founders grown in the field (July 23, 2024). (a) Comparison of rates of linear electron flow (LEFamb) against ambient PAR (PARamb). (b) Rates of LEF (LEFhigh) following 10 sec illumination at high light, compared to ambient PAR. (c) The difference between rates of LEF under ambient and high light, compared to ambient PAR. (d) Comparison of phiPSII measured under ambient and high light (yellow symbols), versus ambient PAR (gray symbols). Figure S3. Direct measurement of NPQ in NAM population founders grown in the field (July 30, 2024). (a) Comparison of rates of linear electron flow (LEFamb) against ambient PAR (PARamb). (b) Rates of LEF (LEFhigh) following 10 sec illumination at high light, compared to ambient PAR. (c) The difference between rates of LEF under ambient and high light, compared to ambient PAR. (d) Comparison of phiPSII measured under ambient and high light (yellow symbols), versus ambient PAR (gray symbols). Figure S4. Comparison of NPQt values for the SoyNAM founders on July 23, 2024. (a) Boxplot comparing NPQt values recorded for SoyNAM founders under high light. Values represent the mean of three technical (individual plant) replicates per plot (n = 5). (b) Comparison of NPQt measured under ambient light (PARamb) and NPQt (NPQtamb), individual technical replicates are shown. (c) Comparison of NPQt measured under high light (NPQthigh) and ambient PAR. Individual technical replicates are shown. Figure S5. Comparison of NPQt values for the SoyNAM founders on July 30, 2024. (a) Boxplot comparing NPQt values recorded for SoyNAM founder [file TPJ-121-0-s012.zip › Figure_S10_PCA_day71222.tiff]

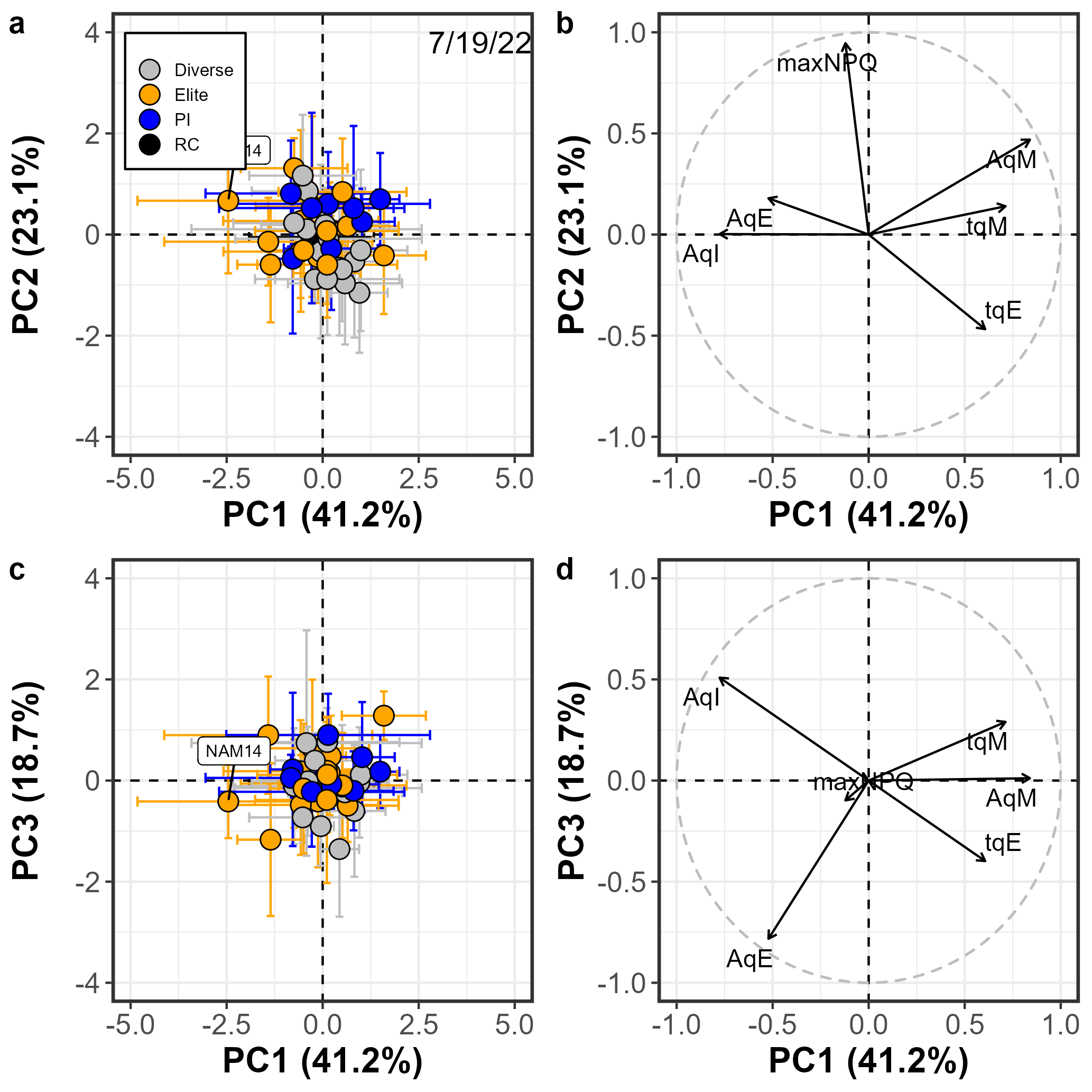

Supplement: Supplementary file 3 — Figure S1. Comparison of genotypic means for NPQ relaxation parameters measured in 2021 and 2022 using the SoyNAM founders. Scatterplots comparing values of (a) maximum inducible NPQ, (b) A qE, (c) A qM, (d) A qI, (e) τ qE, and (f) τ qM. Values represent the mean of seasonal measurements. Pearson correlation coefficient (R) and P‐value are reported for each parameter. Figure S2. Direct measurement of NPQ in NAM population founders grown in the field (July 23, 2024). (a) Comparison of rates of linear electron flow (LEFamb) against ambient PAR (PARamb). (b) Rates of LEF (LEFhigh) following 10 sec illumination at high light, compared to ambient PAR. (c) The difference between rates of LEF under ambient and high light, compared to ambient PAR. (d) Comparison of phiPSII measured under ambient and high light (yellow symbols), versus ambient PAR (gray symbols). Figure S3. Direct measurement of NPQ in NAM population founders grown in the field (July 30, 2024). (a) Comparison of rates of linear electron flow (LEFamb) against ambient PAR (PARamb). (b) Rates of LEF (LEFhigh) following 10 sec illumination at high light, compared to ambient PAR. (c) The difference between rates of LEF under ambient and high light, compared to ambient PAR. (d) Comparison of phiPSII measured under ambient and high light (yellow symbols), versus ambient PAR (gray symbols). Figure S4. Comparison of NPQt values for the SoyNAM founders on July 23, 2024. (a) Boxplot comparing NPQt values recorded for SoyNAM founders under high light. Values represent the mean of three technical (individual plant) replicates per plot (n = 5). (b) Comparison of NPQt measured under ambient light (PARamb) and NPQt (NPQtamb), individual technical replicates are shown. (c) Comparison of NPQt measured under high light (NPQthigh) and ambient PAR. Individual technical replicates are shown. Figure S5. Comparison of NPQt values for the SoyNAM founders on July 30, 2024. (a) Boxplot comparing NPQt values recorded for SoyNAM founder [file TPJ-121-0-s012.zip › Figure_S11_PCA_day71922.tiff]

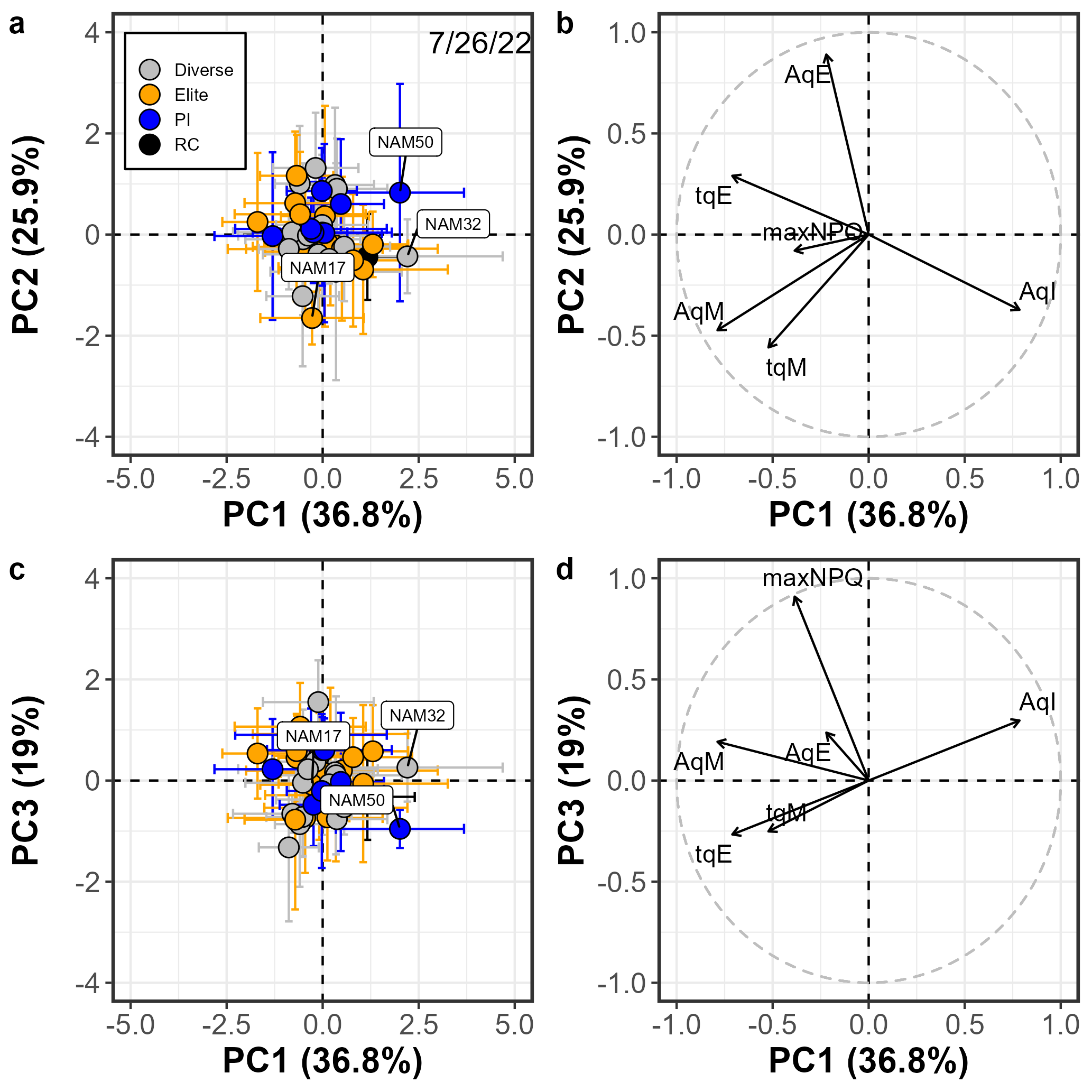

Supplement: Supplementary file 3 — Figure S1. Comparison of genotypic means for NPQ relaxation parameters measured in 2021 and 2022 using the SoyNAM founders. Scatterplots comparing values of (a) maximum inducible NPQ, (b) A qE, (c) A qM, (d) A qI, (e) τ qE, and (f) τ qM. Values represent the mean of seasonal measurements. Pearson correlation coefficient (R) and P‐value are reported for each parameter. Figure S2. Direct measurement of NPQ in NAM population founders grown in the field (July 23, 2024). (a) Comparison of rates of linear electron flow (LEFamb) against ambient PAR (PARamb). (b) Rates of LEF (LEFhigh) following 10 sec illumination at high light, compared to ambient PAR. (c) The difference between rates of LEF under ambient and high light, compared to ambient PAR. (d) Comparison of phiPSII measured under ambient and high light (yellow symbols), versus ambient PAR (gray symbols). Figure S3. Direct measurement of NPQ in NAM population founders grown in the field (July 30, 2024). (a) Comparison of rates of linear electron flow (LEFamb) against ambient PAR (PARamb). (b) Rates of LEF (LEFhigh) following 10 sec illumination at high light, compared to ambient PAR. (c) The difference between rates of LEF under ambient and high light, compared to ambient PAR. (d) Comparison of phiPSII measured under ambient and high light (yellow symbols), versus ambient PAR (gray symbols). Figure S4. Comparison of NPQt values for the SoyNAM founders on July 23, 2024. (a) Boxplot comparing NPQt values recorded for SoyNAM founders under high light. Values represent the mean of three technical (individual plant) replicates per plot (n = 5). (b) Comparison of NPQt measured under ambient light (PARamb) and NPQt (NPQtamb), individual technical replicates are shown. (c) Comparison of NPQt measured under high light (NPQthigh) and ambient PAR. Individual technical replicates are shown. Figure S5. Comparison of NPQt values for the SoyNAM founders on July 30, 2024. (a) Boxplot comparing NPQt values recorded for SoyNAM founder [file TPJ-121-0-s012.zip › Figure_S12_PCA_day72622.tiff]

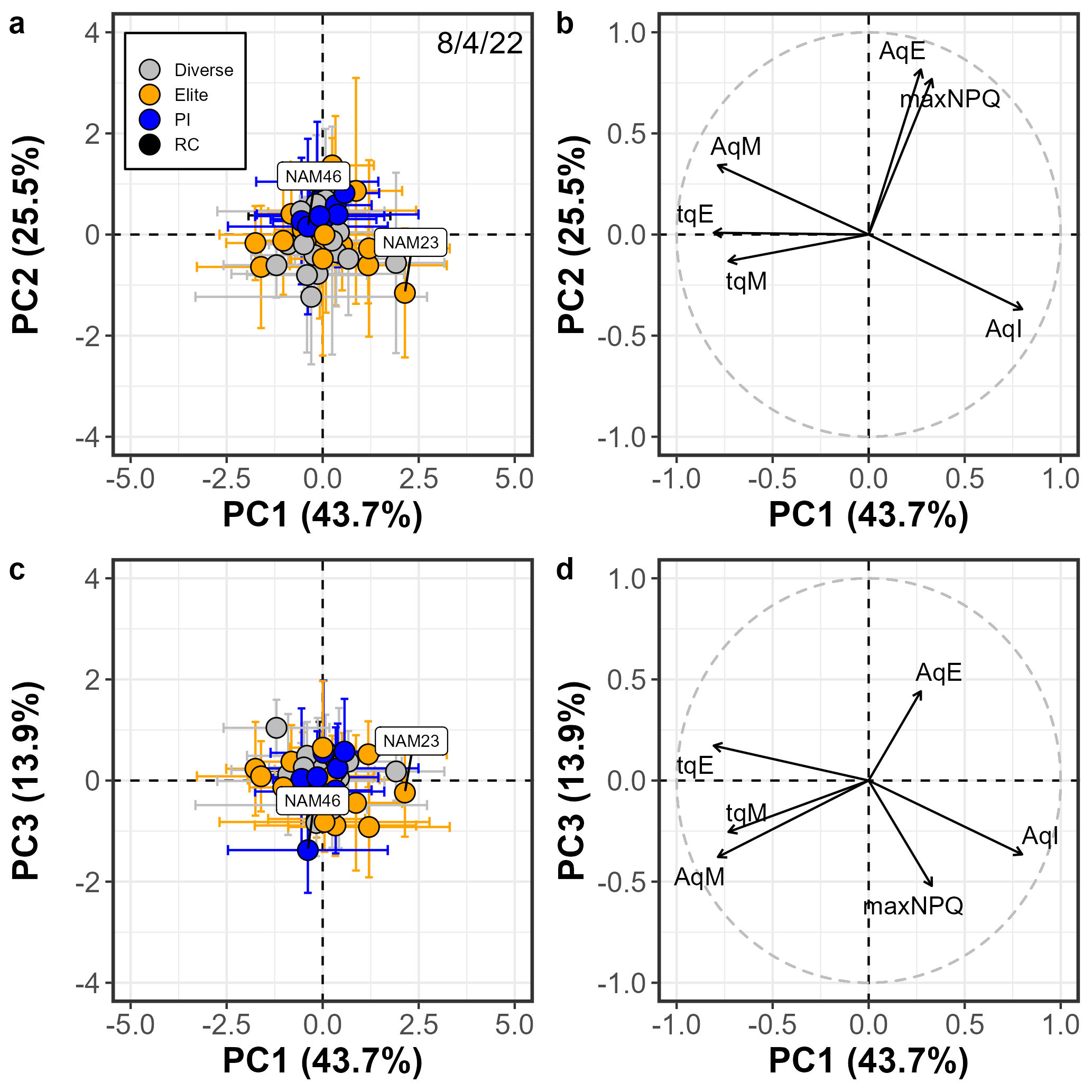

Supplement: Supplementary file 3 — Figure S1. Comparison of genotypic means for NPQ relaxation parameters measured in 2021 and 2022 using the SoyNAM founders. Scatterplots comparing values of (a) maximum inducible NPQ, (b) A qE, (c) A qM, (d) A qI, (e) τ qE, and (f) τ qM. Values represent the mean of seasonal measurements. Pearson correlation coefficient (R) and P‐value are reported for each parameter. Figure S2. Direct measurement of NPQ in NAM population founders grown in the field (July 23, 2024). (a) Comparison of rates of linear electron flow (LEFamb) against ambient PAR (PARamb). (b) Rates of LEF (LEFhigh) following 10 sec illumination at high light, compared to ambient PAR. (c) The difference between rates of LEF under ambient and high light, compared to ambient PAR. (d) Comparison of phiPSII measured under ambient and high light (yellow symbols), versus ambient PAR (gray symbols). Figure S3. Direct measurement of NPQ in NAM population founders grown in the field (July 30, 2024). (a) Comparison of rates of linear electron flow (LEFamb) against ambient PAR (PARamb). (b) Rates of LEF (LEFhigh) following 10 sec illumination at high light, compared to ambient PAR. (c) The difference between rates of LEF under ambient and high light, compared to ambient PAR. (d) Comparison of phiPSII measured under ambient and high light (yellow symbols), versus ambient PAR (gray symbols). Figure S4. Comparison of NPQt values for the SoyNAM founders on July 23, 2024. (a) Boxplot comparing NPQt values recorded for SoyNAM founders under high light. Values represent the mean of three technical (individual plant) replicates per plot (n = 5). (b) Comparison of NPQt measured under ambient light (PARamb) and NPQt (NPQtamb), individual technical replicates are shown. (c) Comparison of NPQt measured under high light (NPQthigh) and ambient PAR. Individual technical replicates are shown. Figure S5. Comparison of NPQt values for the SoyNAM founders on July 30, 2024. (a) Boxplot comparing NPQt values recorded for SoyNAM founder [file TPJ-121-0-s012.zip › Figure_S13_PCA_day8422.tiff]

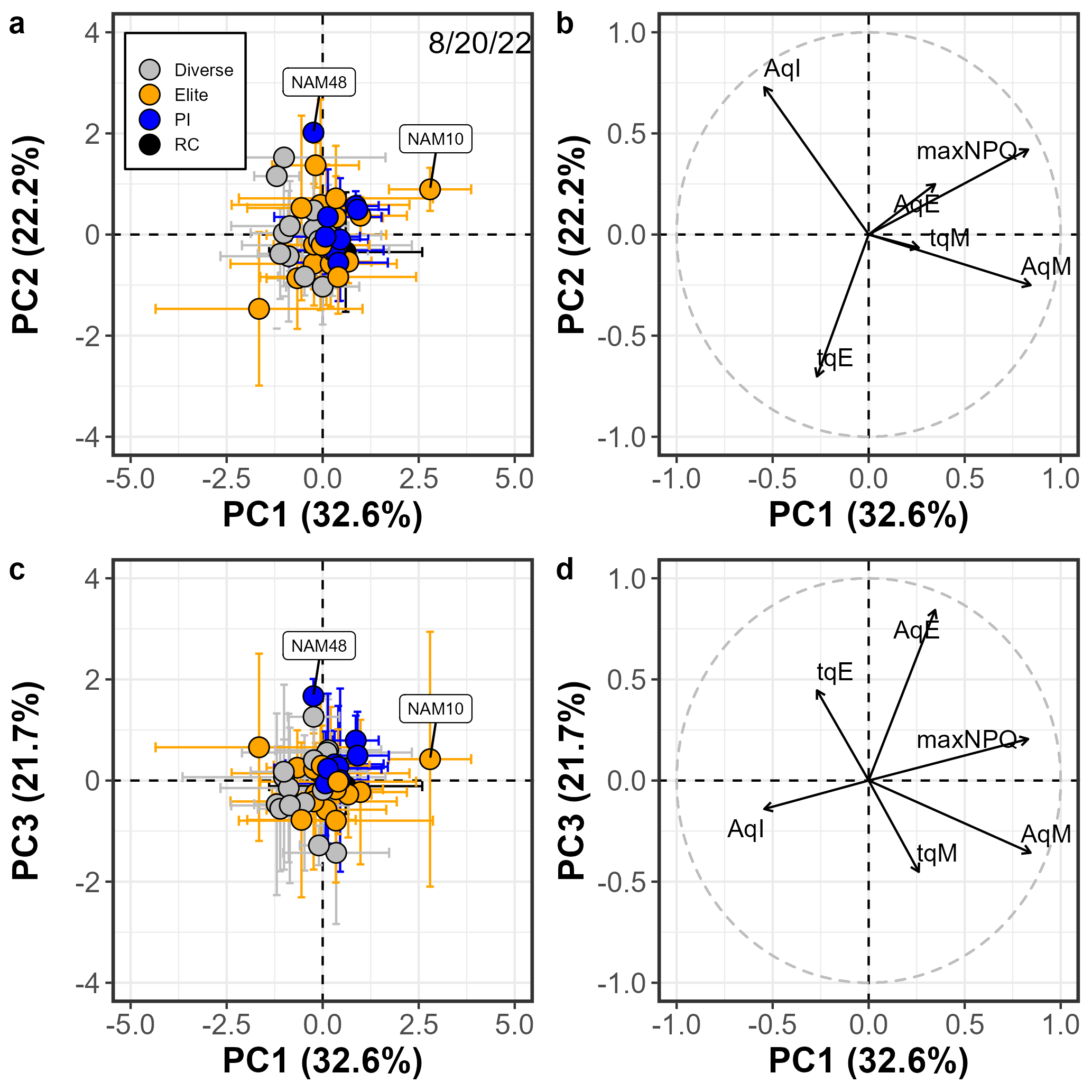

Supplement: Supplementary file 3 — Figure S1. Comparison of genotypic means for NPQ relaxation parameters measured in 2021 and 2022 using the SoyNAM founders. Scatterplots comparing values of (a) maximum inducible NPQ, (b) A qE, (c) A qM, (d) A qI, (e) τ qE, and (f) τ qM. Values represent the mean of seasonal measurements. Pearson correlation coefficient (R) and P‐value are reported for each parameter. Figure S2. Direct measurement of NPQ in NAM population founders grown in the field (July 23, 2024). (a) Comparison of rates of linear electron flow (LEFamb) against ambient PAR (PARamb). (b) Rates of LEF (LEFhigh) following 10 sec illumination at high light, compared to ambient PAR. (c) The difference between rates of LEF under ambient and high light, compared to ambient PAR. (d) Comparison of phiPSII measured under ambient and high light (yellow symbols), versus ambient PAR (gray symbols). Figure S3. Direct measurement of NPQ in NAM population founders grown in the field (July 30, 2024). (a) Comparison of rates of linear electron flow (LEFamb) against ambient PAR (PARamb). (b) Rates of LEF (LEFhigh) following 10 sec illumination at high light, compared to ambient PAR. (c) The difference between rates of LEF under ambient and high light, compared to ambient PAR. (d) Comparison of phiPSII measured under ambient and high light (yellow symbols), versus ambient PAR (gray symbols). Figure S4. Comparison of NPQt values for the SoyNAM founders on July 23, 2024. (a) Boxplot comparing NPQt values recorded for SoyNAM founders under high light. Values represent the mean of three technical (individual plant) replicates per plot (n = 5). (b) Comparison of NPQt measured under ambient light (PARamb) and NPQt (NPQtamb), individual technical replicates are shown. (c) Comparison of NPQt measured under high light (NPQthigh) and ambient PAR. Individual technical replicates are shown. Figure S5. Comparison of NPQt values for the SoyNAM founders on July 30, 2024. (a) Boxplot comparing NPQt values recorded for SoyNAM founder [file TPJ-121-0-s012.zip › Figure_S14_PCA_day82022.tiff]

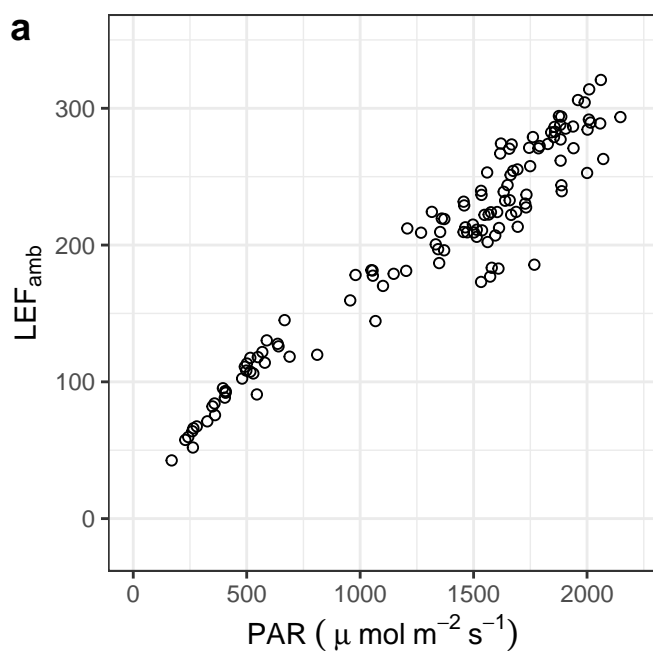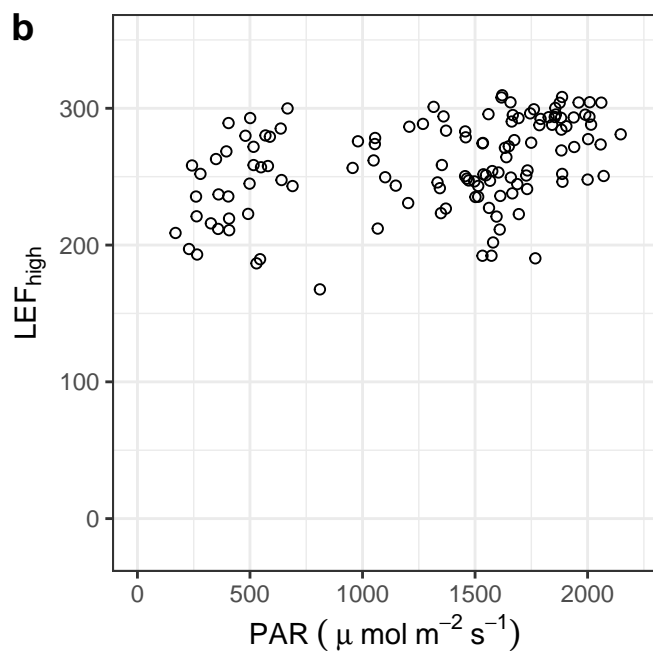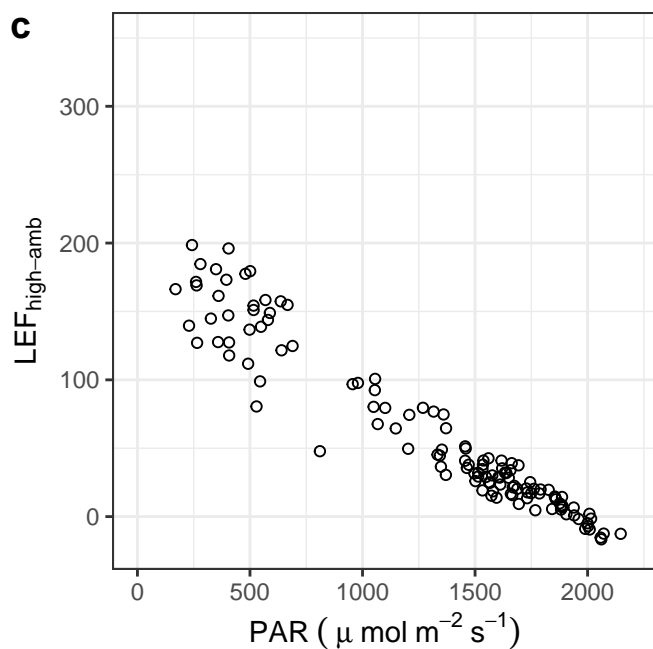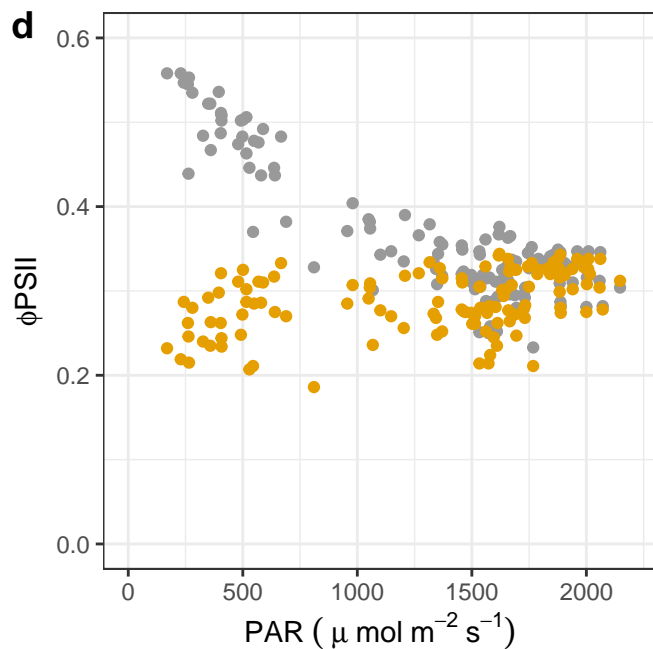

Supplement: Supplementary file 3 — Figure S1. Comparison of genotypic means for NPQ relaxation parameters measured in 2021 and 2022 using the SoyNAM founders. Scatterplots comparing values of (a) maximum inducible NPQ, (b) A qE, (c) A qM, (d) A qI, (e) τ qE, and (f) τ qM. Values represent the mean of seasonal measurements. Pearson correlation coefficient (R) and P‐value are reported for each parameter. Figure S2. Direct measurement of NPQ in NAM population founders grown in the field (July 23, 2024). (a) Comparison of rates of linear electron flow (LEFamb) against ambient PAR (PARamb). (b) Rates of LEF (LEFhigh) following 10 sec illumination at high light, compared to ambient PAR. (c) The difference between rates of LEF under ambient and high light, compared to ambient PAR. (d) Comparison of phiPSII measured under ambient and high light (yellow symbols), versus ambient PAR (gray symbols). Figure S3. Direct measurement of NPQ in NAM population founders grown in the field (July 30, 2024). (a) Comparison of rates of linear electron flow (LEFamb) against ambient PAR (PARamb). (b) Rates of LEF (LEFhigh) following 10 sec illumination at high light, compared to ambient PAR. (c) The difference between rates of LEF under ambient and high light, compared to ambient PAR. (d) Comparison of phiPSII measured under ambient and high light (yellow symbols), versus ambient PAR (gray symbols). Figure S4. Comparison of NPQt values for the SoyNAM founders on July 23, 2024. (a) Boxplot comparing NPQt values recorded for SoyNAM founders under high light. Values represent the mean of three technical (individual plant) replicates per plot (n = 5). (b) Comparison of NPQt measured under ambient light (PARamb) and NPQt (NPQtamb), individual technical replicates are shown. (c) Comparison of NPQt measured under high light (NPQthigh) and ambient PAR. Individual technical replicates are shown. Figure S5. Comparison of NPQt values for the SoyNAM founders on July 30, 2024. (a) Boxplot comparing NPQt values recorded for SoyNAM founder [file TPJ-121-0-s012.zip › Figure_S2_2024-07-23-NAM_LEF.pdf]

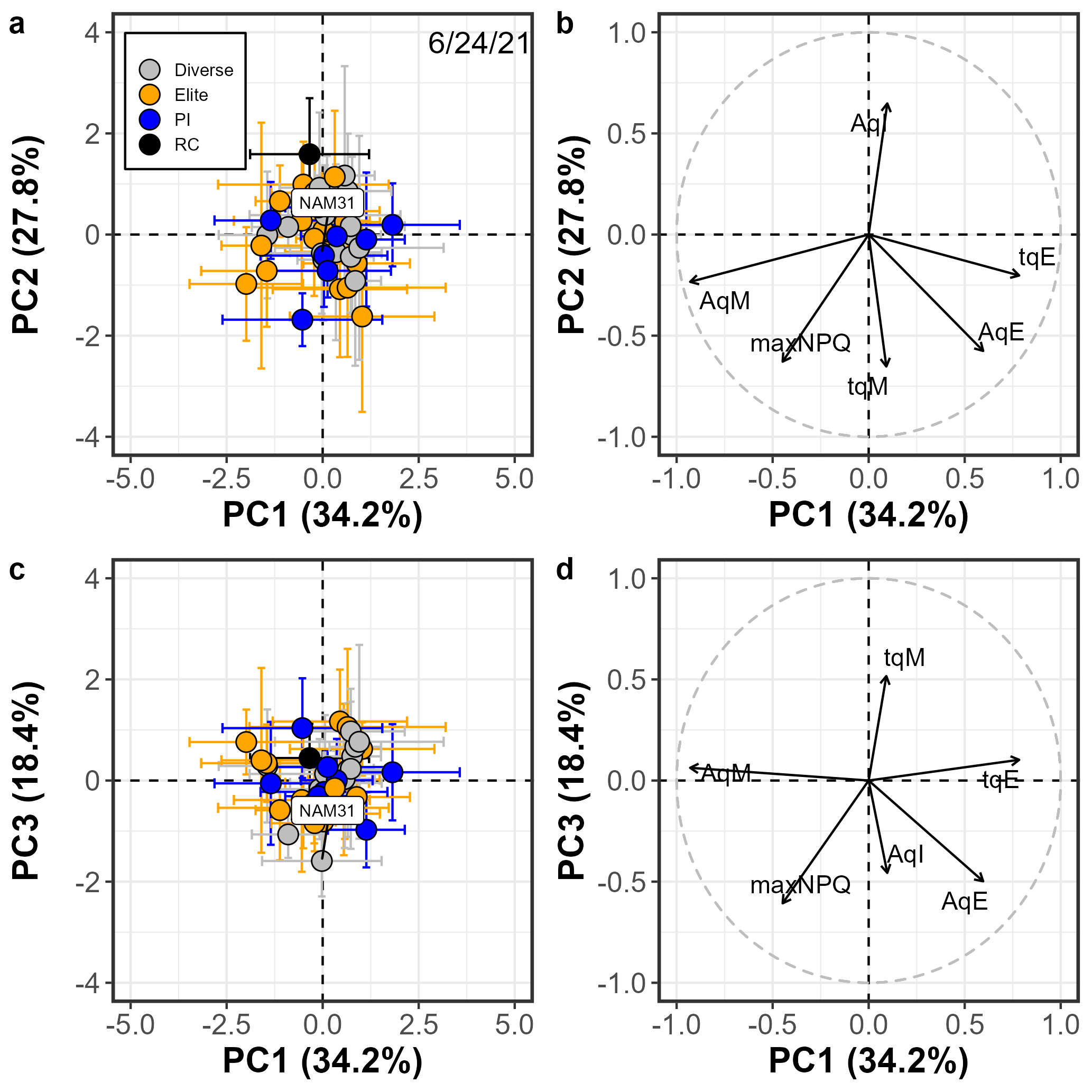

Supplement: Supplementary file 3 — Figure S1. Comparison of genotypic means for NPQ relaxation parameters measured in 2021 and 2022 using the SoyNAM founders. Scatterplots comparing values of (a) maximum inducible NPQ, (b) A qE, (c) A qM, (d) A qI, (e) τ qE, and (f) τ qM. Values represent the mean of seasonal measurements. Pearson correlation coefficient (R) and P‐value are reported for each parameter. Figure S2. Direct measurement of NPQ in NAM population founders grown in the field (July 23, 2024). (a) Comparison of rates of linear electron flow (LEFamb) against ambient PAR (PARamb). (b) Rates of LEF (LEFhigh) following 10 sec illumination at high light, compared to ambient PAR. (c) The difference between rates of LEF under ambient and high light, compared to ambient PAR. (d) Comparison of phiPSII measured under ambient and high light (yellow symbols), versus ambient PAR (gray symbols). Figure S3. Direct measurement of NPQ in NAM population founders grown in the field (July 30, 2024). (a) Comparison of rates of linear electron flow (LEFamb) against ambient PAR (PARamb). (b) Rates of LEF (LEFhigh) following 10 sec illumination at high light, compared to ambient PAR. (c) The difference between rates of LEF under ambient and high light, compared to ambient PAR. (d) Comparison of phiPSII measured under ambient and high light (yellow symbols), versus ambient PAR (gray symbols). Figure S4. Comparison of NPQt values for the SoyNAM founders on July 23, 2024. (a) Boxplot comparing NPQt values recorded for SoyNAM founders under high light. Values represent the mean of three technical (individual plant) replicates per plot (n = 5). (b) Comparison of NPQt measured under ambient light (PARamb) and NPQt (NPQtamb), individual technical replicates are shown. (c) Comparison of NPQt measured under high light (NPQthigh) and ambient PAR. Individual technical replicates are shown. Figure S5. Comparison of NPQt values for the SoyNAM founders on July 30, 2024. (a) Boxplot comparing NPQt values recorded for SoyNAM founder [file TPJ-121-0-s012.zip › Figure_S2_PCA_day62421.tiff]

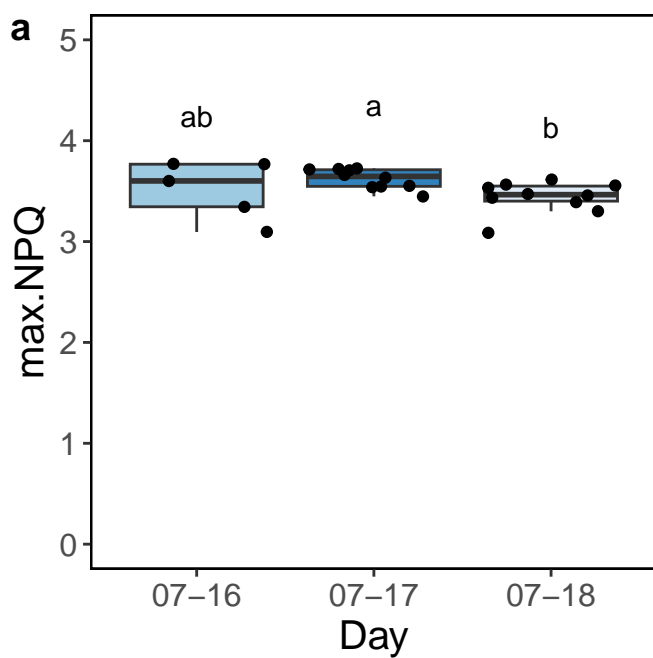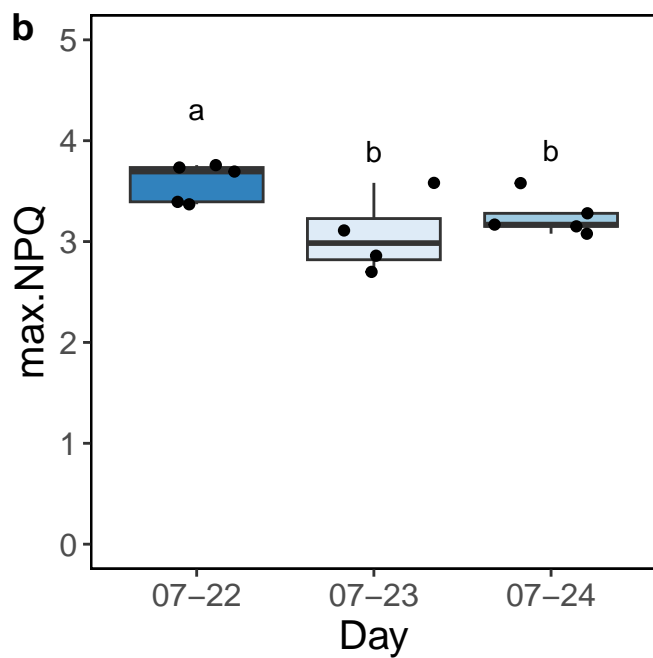

Supplement: Supplementary file 3 — Figure S1. Comparison of genotypic means for NPQ relaxation parameters measured in 2021 and 2022 using the SoyNAM founders. Scatterplots comparing values of (a) maximum inducible NPQ, (b) A qE, (c) A qM, (d) A qI, (e) τ qE, and (f) τ qM. Values represent the mean of seasonal measurements. Pearson correlation coefficient (R) and P‐value are reported for each parameter. Figure S2. Direct measurement of NPQ in NAM population founders grown in the field (July 23, 2024). (a) Comparison of rates of linear electron flow (LEFamb) against ambient PAR (PARamb). (b) Rates of LEF (LEFhigh) following 10 sec illumination at high light, compared to ambient PAR. (c) The difference between rates of LEF under ambient and high light, compared to ambient PAR. (d) Comparison of phiPSII measured under ambient and high light (yellow symbols), versus ambient PAR (gray symbols). Figure S3. Direct measurement of NPQ in NAM population founders grown in the field (July 30, 2024). (a) Comparison of rates of linear electron flow (LEFamb) against ambient PAR (PARamb). (b) Rates of LEF (LEFhigh) following 10 sec illumination at high light, compared to ambient PAR. (c) The difference between rates of LEF under ambient and high light, compared to ambient PAR. (d) Comparison of phiPSII measured under ambient and high light (yellow symbols), versus ambient PAR (gray symbols). Figure S4. Comparison of NPQt values for the SoyNAM founders on July 23, 2024. (a) Boxplot comparing NPQt values recorded for SoyNAM founders under high light. Values represent the mean of three technical (individual plant) replicates per plot (n = 5). (b) Comparison of NPQt measured under ambient light (PARamb) and NPQt (NPQtamb), individual technical replicates are shown. (c) Comparison of NPQt measured under high light (NPQthigh) and ambient PAR. Individual technical replicates are shown. Figure S5. Comparison of NPQt values for the SoyNAM founders on July 30, 2024. (a) Boxplot comparing NPQt values recorded for SoyNAM founder [file TPJ-121-0-s012.zip › Figure_S23_days.pdf]

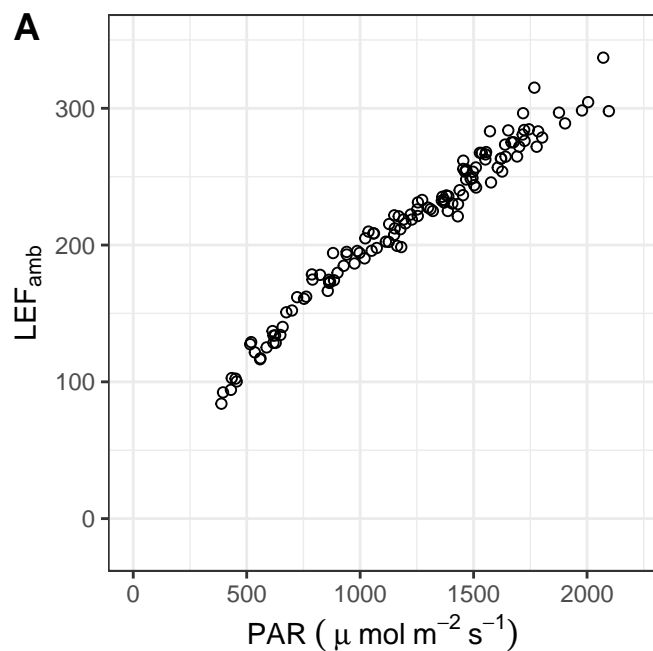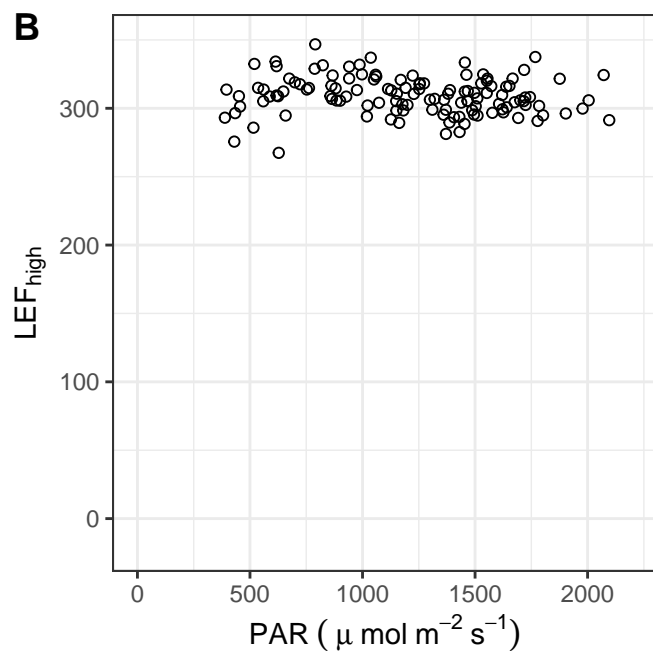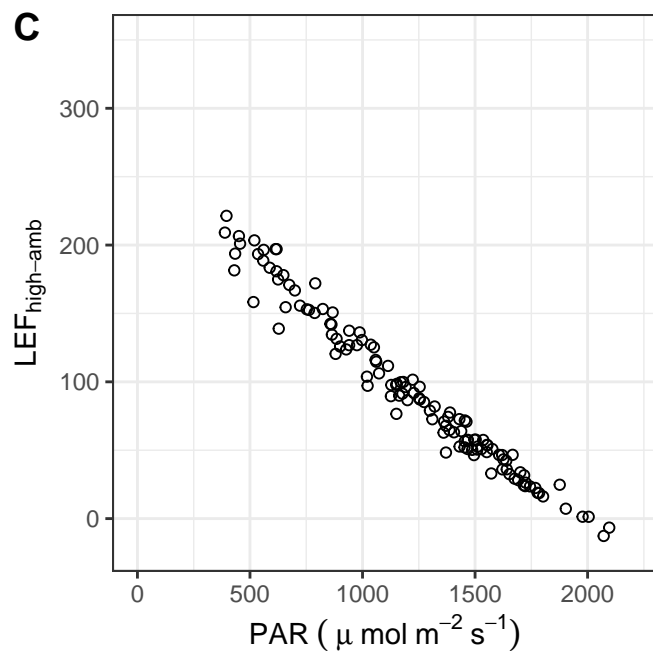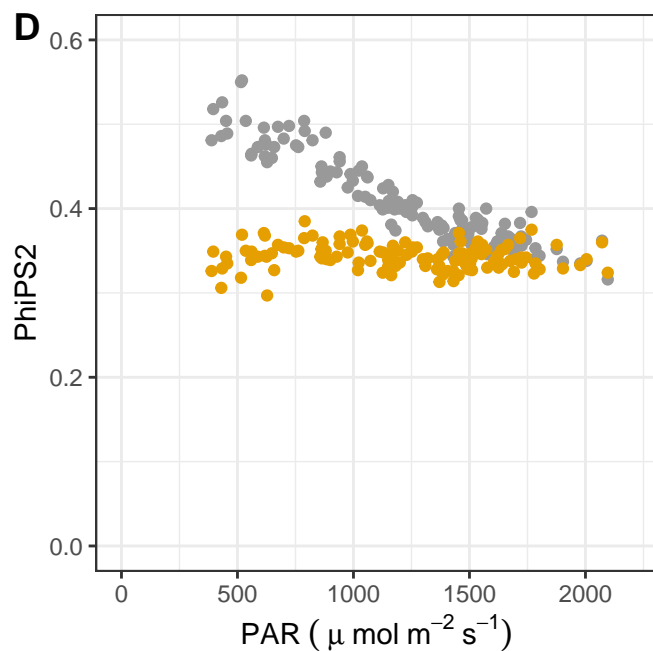

Supplement: Supplementary file 3 — Figure S1. Comparison of genotypic means for NPQ relaxation parameters measured in 2021 and 2022 using the SoyNAM founders. Scatterplots comparing values of (a) maximum inducible NPQ, (b) A qE, (c) A qM, (d) A qI, (e) τ qE, and (f) τ qM. Values represent the mean of seasonal measurements. Pearson correlation coefficient (R) and P‐value are reported for each parameter. Figure S2. Direct measurement of NPQ in NAM population founders grown in the field (July 23, 2024). (a) Comparison of rates of linear electron flow (LEFamb) against ambient PAR (PARamb). (b) Rates of LEF (LEFhigh) following 10 sec illumination at high light, compared to ambient PAR. (c) The difference between rates of LEF under ambient and high light, compared to ambient PAR. (d) Comparison of phiPSII measured under ambient and high light (yellow symbols), versus ambient PAR (gray symbols). Figure S3. Direct measurement of NPQ in NAM population founders grown in the field (July 30, 2024). (a) Comparison of rates of linear electron flow (LEFamb) against ambient PAR (PARamb). (b) Rates of LEF (LEFhigh) following 10 sec illumination at high light, compared to ambient PAR. (c) The difference between rates of LEF under ambient and high light, compared to ambient PAR. (d) Comparison of phiPSII measured under ambient and high light (yellow symbols), versus ambient PAR (gray symbols). Figure S4. Comparison of NPQt values for the SoyNAM founders on July 23, 2024. (a) Boxplot comparing NPQt values recorded for SoyNAM founders under high light. Values represent the mean of three technical (individual plant) replicates per plot (n = 5). (b) Comparison of NPQt measured under ambient light (PARamb) and NPQt (NPQtamb), individual technical replicates are shown. (c) Comparison of NPQt measured under high light (NPQthigh) and ambient PAR. Individual technical replicates are shown. Figure S5. Comparison of NPQt values for the SoyNAM founders on July 30, 2024. (a) Boxplot comparing NPQt values recorded for SoyNAM founder [file TPJ-121-0-s012.zip › Figure_S3_NAM_LEF_24_07_30.pdf]

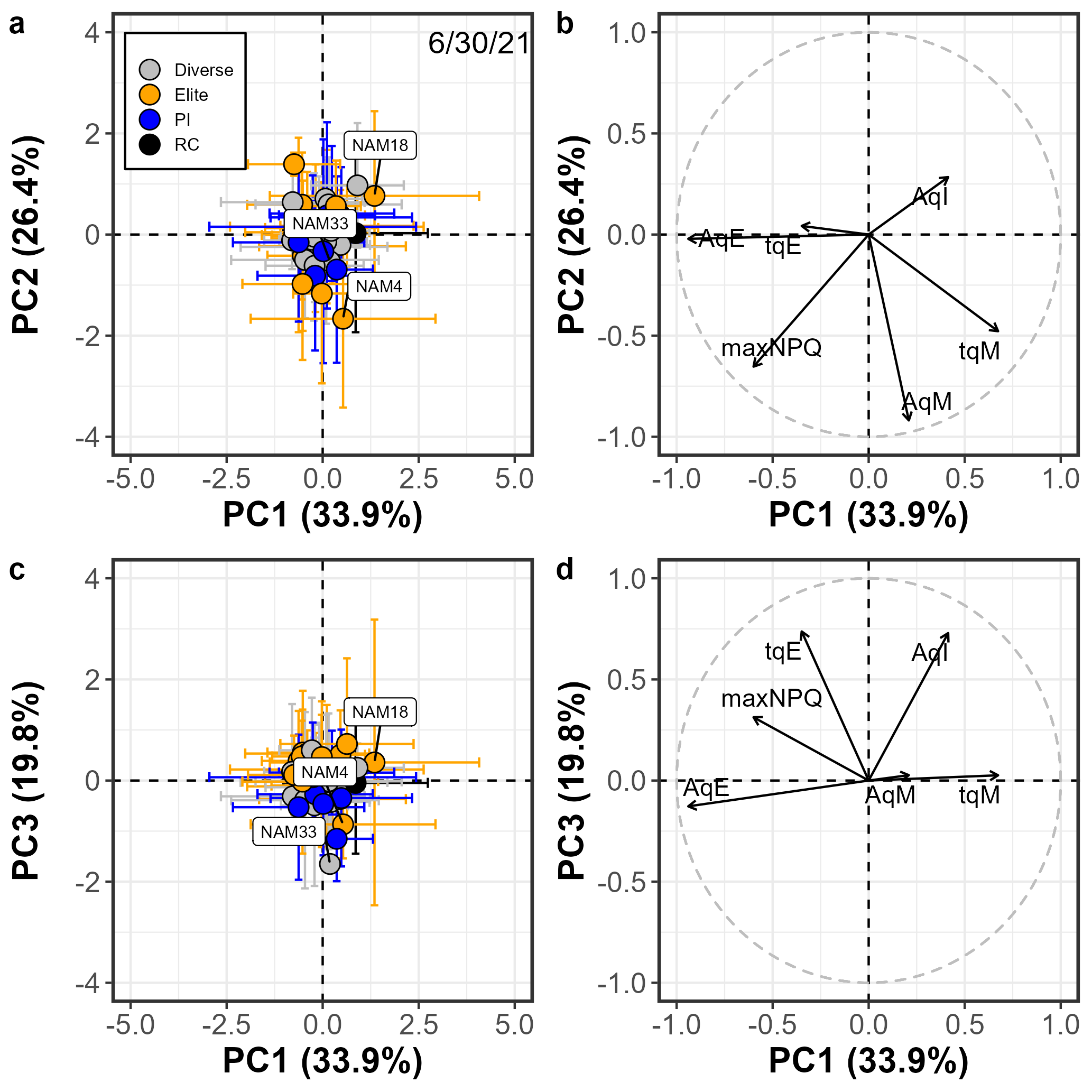

Supplement: Supplementary file 3 — Figure S1. Comparison of genotypic means for NPQ relaxation parameters measured in 2021 and 2022 using the SoyNAM founders. Scatterplots comparing values of (a) maximum inducible NPQ, (b) A qE, (c) A qM, (d) A qI, (e) τ qE, and (f) τ qM. Values represent the mean of seasonal measurements. Pearson correlation coefficient (R) and P‐value are reported for each parameter. Figure S2. Direct measurement of NPQ in NAM population founders grown in the field (July 23, 2024). (a) Comparison of rates of linear electron flow (LEFamb) against ambient PAR (PARamb). (b) Rates of LEF (LEFhigh) following 10 sec illumination at high light, compared to ambient PAR. (c) The difference between rates of LEF under ambient and high light, compared to ambient PAR. (d) Comparison of phiPSII measured under ambient and high light (yellow symbols), versus ambient PAR (gray symbols). Figure S3. Direct measurement of NPQ in NAM population founders grown in the field (July 30, 2024). (a) Comparison of rates of linear electron flow (LEFamb) against ambient PAR (PARamb). (b) Rates of LEF (LEFhigh) following 10 sec illumination at high light, compared to ambient PAR. (c) The difference between rates of LEF under ambient and high light, compared to ambient PAR. (d) Comparison of phiPSII measured under ambient and high light (yellow symbols), versus ambient PAR (gray symbols). Figure S4. Comparison of NPQt values for the SoyNAM founders on July 23, 2024. (a) Boxplot comparing NPQt values recorded for SoyNAM founders under high light. Values represent the mean of three technical (individual plant) replicates per plot (n = 5). (b) Comparison of NPQt measured under ambient light (PARamb) and NPQt (NPQtamb), individual technical replicates are shown. (c) Comparison of NPQt measured under high light (NPQthigh) and ambient PAR. Individual technical replicates are shown. Figure S5. Comparison of NPQt values for the SoyNAM founders on July 30, 2024. (a) Boxplot comparing NPQt values recorded for SoyNAM founder [file TPJ-121-0-s012.zip › Figure_S3_PCA_day63021.tiff]

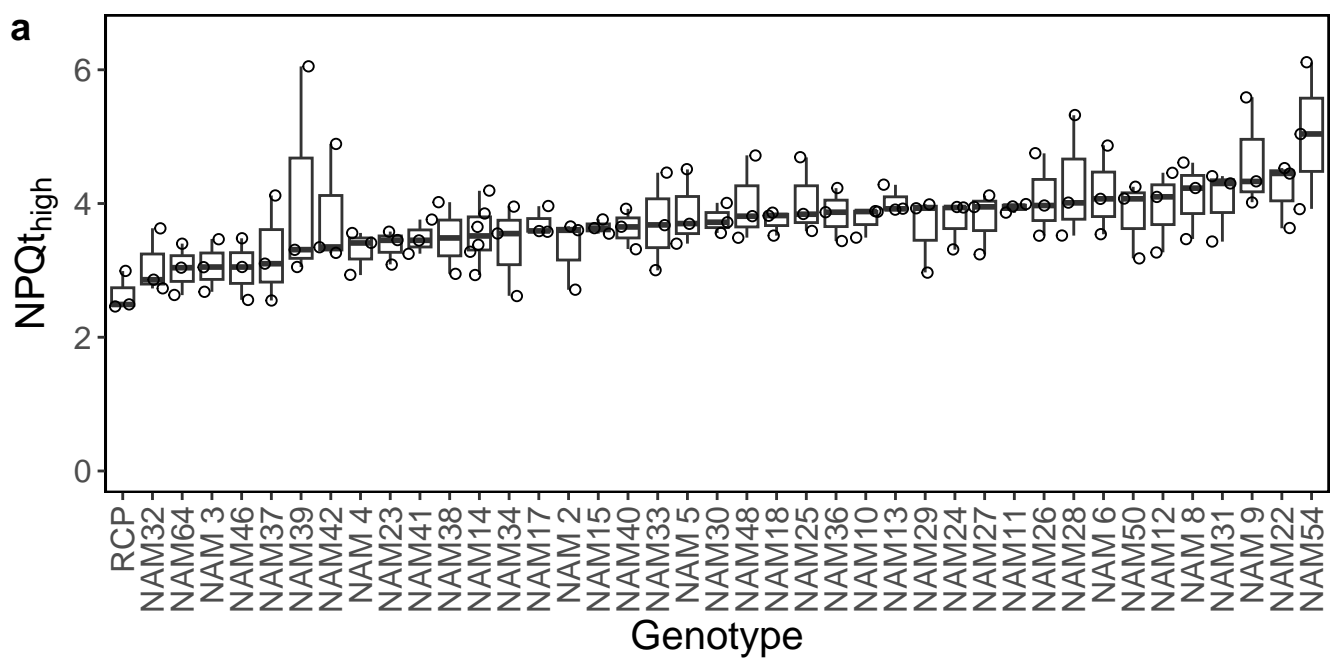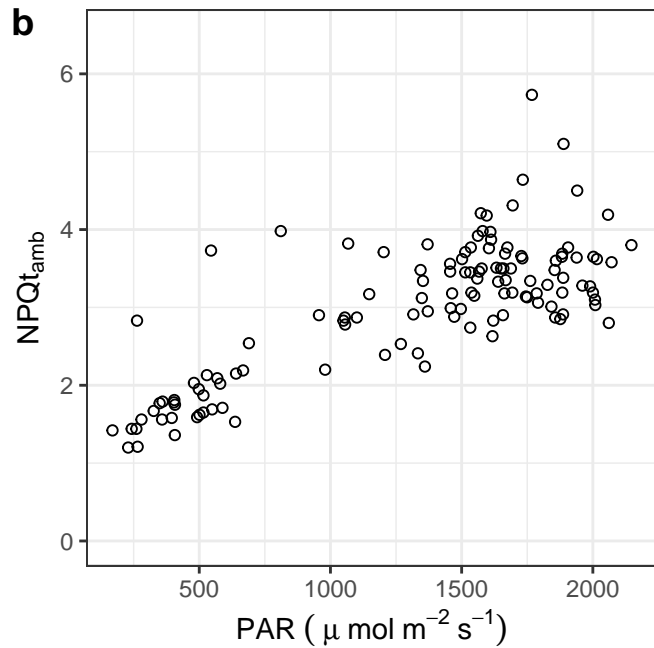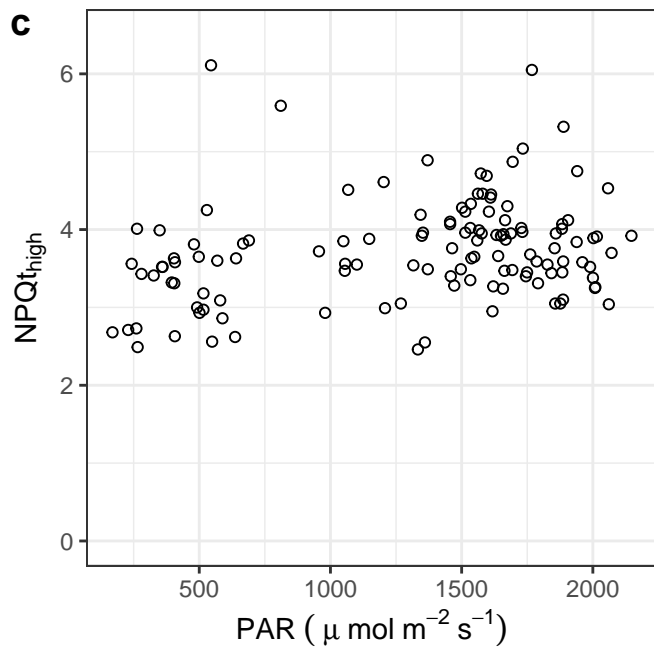

Supplement: Supplementary file 3 — Figure S1. Comparison of genotypic means for NPQ relaxation parameters measured in 2021 and 2022 using the SoyNAM founders. Scatterplots comparing values of (a) maximum inducible NPQ, (b) A qE, (c) A qM, (d) A qI, (e) τ qE, and (f) τ qM. Values represent the mean of seasonal measurements. Pearson correlation coefficient (R) and P‐value are reported for each parameter. Figure S2. Direct measurement of NPQ in NAM population founders grown in the field (July 23, 2024). (a) Comparison of rates of linear electron flow (LEFamb) against ambient PAR (PARamb). (b) Rates of LEF (LEFhigh) following 10 sec illumination at high light, compared to ambient PAR. (c) The difference between rates of LEF under ambient and high light, compared to ambient PAR. (d) Comparison of phiPSII measured under ambient and high light (yellow symbols), versus ambient PAR (gray symbols). Figure S3. Direct measurement of NPQ in NAM population founders grown in the field (July 30, 2024). (a) Comparison of rates of linear electron flow (LEFamb) against ambient PAR (PARamb). (b) Rates of LEF (LEFhigh) following 10 sec illumination at high light, compared to ambient PAR. (c) The difference between rates of LEF under ambient and high light, compared to ambient PAR. (d) Comparison of phiPSII measured under ambient and high light (yellow symbols), versus ambient PAR (gray symbols). Figure S4. Comparison of NPQt values for the SoyNAM founders on July 23, 2024. (a) Boxplot comparing NPQt values recorded for SoyNAM founders under high light. Values represent the mean of three technical (individual plant) replicates per plot (n = 5). (b) Comparison of NPQt measured under ambient light (PARamb) and NPQt (NPQtamb), individual technical replicates are shown. (c) Comparison of NPQt measured under high light (NPQthigh) and ambient PAR. Individual technical replicates are shown. Figure S5. Comparison of NPQt values for the SoyNAM founders on July 30, 2024. (a) Boxplot comparing NPQt values recorded for SoyNAM founder [file TPJ-121-0-s012.zip › Figure_S4_2024-07-23-NAM_NPQt.pdf]

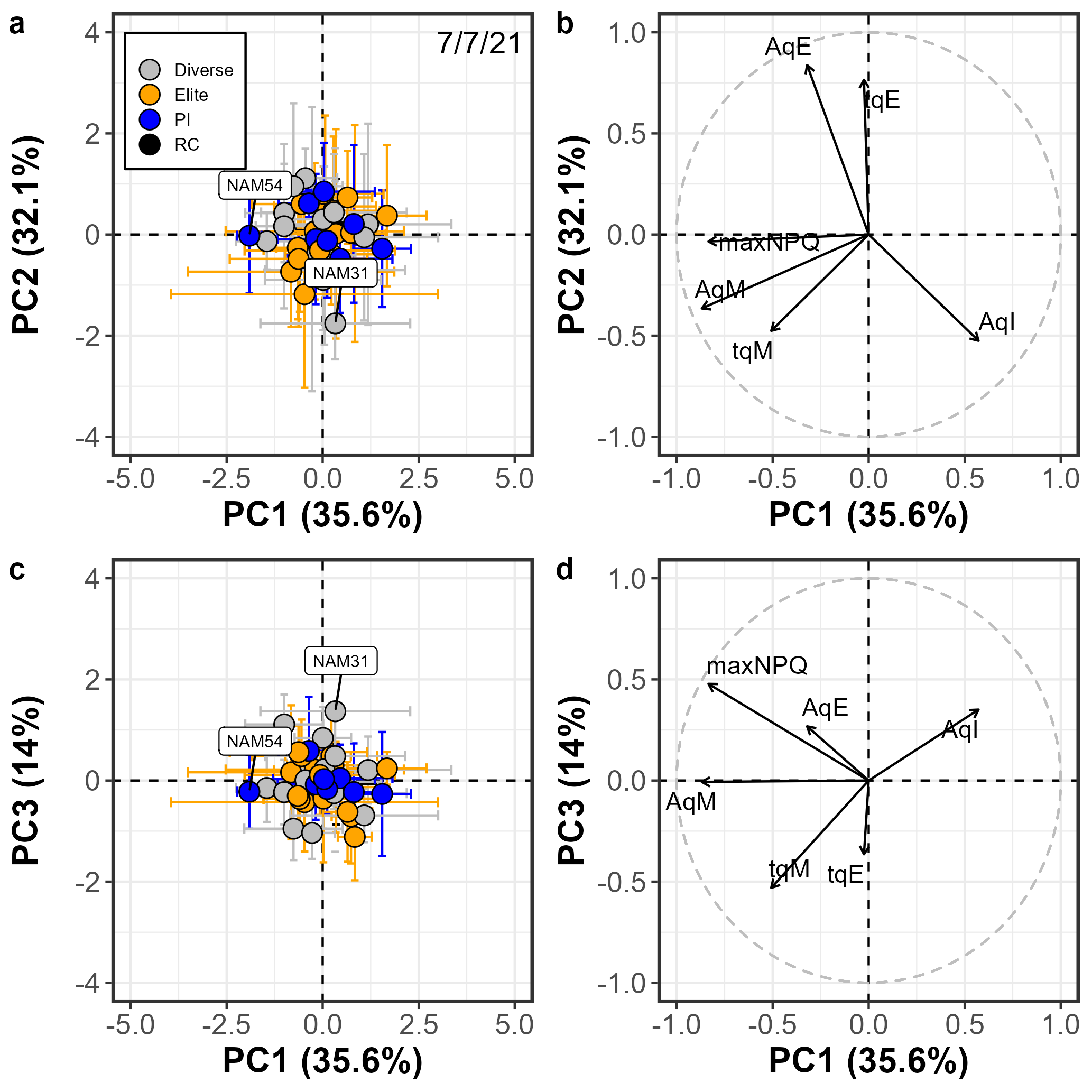

Supplement: Supplementary file 3 — Figure S1. Comparison of genotypic means for NPQ relaxation parameters measured in 2021 and 2022 using the SoyNAM founders. Scatterplots comparing values of (a) maximum inducible NPQ, (b) A qE, (c) A qM, (d) A qI, (e) τ qE, and (f) τ qM. Values represent the mean of seasonal measurements. Pearson correlation coefficient (R) and P‐value are reported for each parameter. Figure S2. Direct measurement of NPQ in NAM population founders grown in the field (July 23, 2024). (a) Comparison of rates of linear electron flow (LEFamb) against ambient PAR (PARamb). (b) Rates of LEF (LEFhigh) following 10 sec illumination at high light, compared to ambient PAR. (c) The difference between rates of LEF under ambient and high light, compared to ambient PAR. (d) Comparison of phiPSII measured under ambient and high light (yellow symbols), versus ambient PAR (gray symbols). Figure S3. Direct measurement of NPQ in NAM population founders grown in the field (July 30, 2024). (a) Comparison of rates of linear electron flow (LEFamb) against ambient PAR (PARamb). (b) Rates of LEF (LEFhigh) following 10 sec illumination at high light, compared to ambient PAR. (c) The difference between rates of LEF under ambient and high light, compared to ambient PAR. (d) Comparison of phiPSII measured under ambient and high light (yellow symbols), versus ambient PAR (gray symbols). Figure S4. Comparison of NPQt values for the SoyNAM founders on July 23, 2024. (a) Boxplot comparing NPQt values recorded for SoyNAM founders under high light. Values represent the mean of three technical (individual plant) replicates per plot (n = 5). (b) Comparison of NPQt measured under ambient light (PARamb) and NPQt (NPQtamb), individual technical replicates are shown. (c) Comparison of NPQt measured under high light (NPQthigh) and ambient PAR. Individual technical replicates are shown. Figure S5. Comparison of NPQt values for the SoyNAM founders on July 30, 2024. (a) Boxplot comparing NPQt values recorded for SoyNAM founder [file TPJ-121-0-s012.zip › Figure_S4_PCA_day7721.tiff]

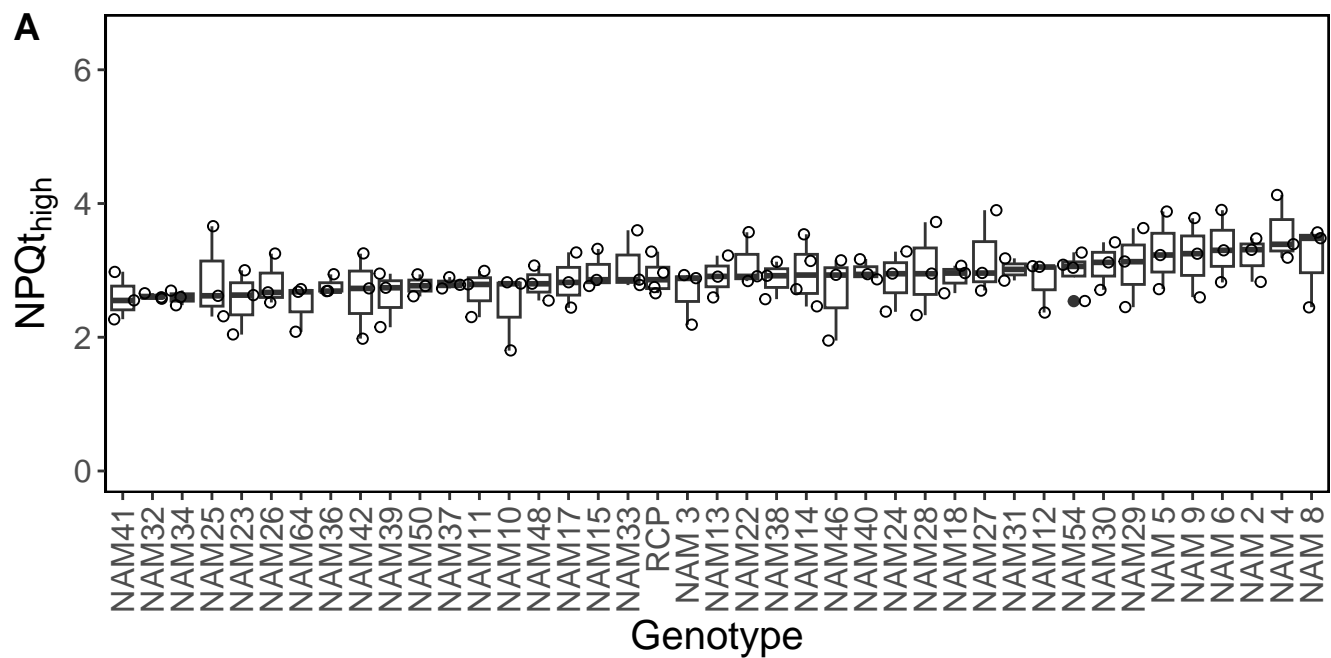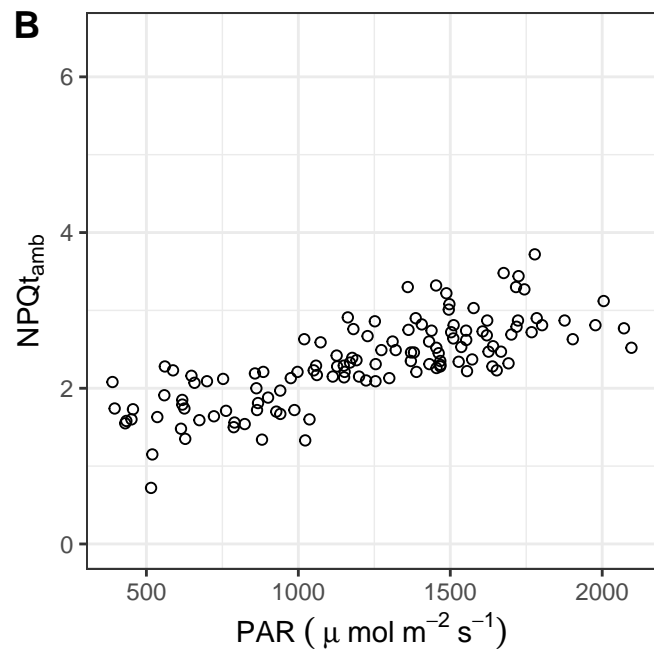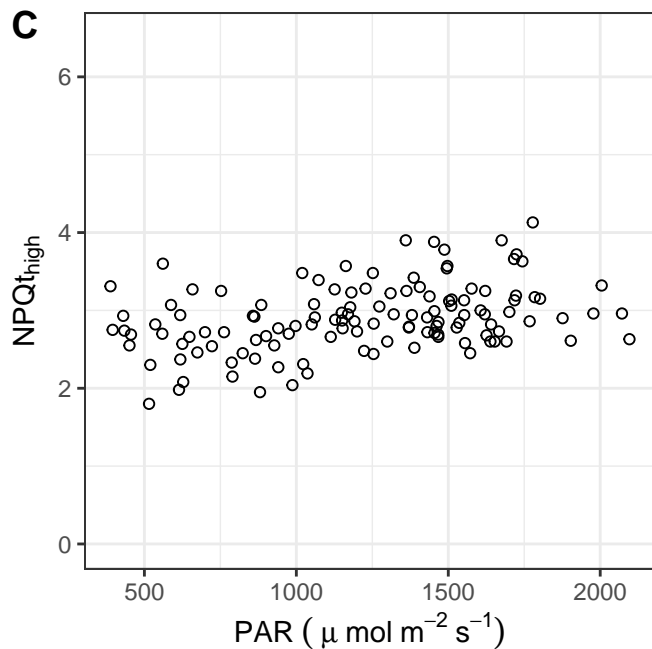

Supplement: Supplementary file 3 — Figure S1. Comparison of genotypic means for NPQ relaxation parameters measured in 2021 and 2022 using the SoyNAM founders. Scatterplots comparing values of (a) maximum inducible NPQ, (b) A qE, (c) A qM, (d) A qI, (e) τ qE, and (f) τ qM. Values represent the mean of seasonal measurements. Pearson correlation coefficient (R) and P‐value are reported for each parameter. Figure S2. Direct measurement of NPQ in NAM population founders grown in the field (July 23, 2024). (a) Comparison of rates of linear electron flow (LEFamb) against ambient PAR (PARamb). (b) Rates of LEF (LEFhigh) following 10 sec illumination at high light, compared to ambient PAR. (c) The difference between rates of LEF under ambient and high light, compared to ambient PAR. (d) Comparison of phiPSII measured under ambient and high light (yellow symbols), versus ambient PAR (gray symbols). Figure S3. Direct measurement of NPQ in NAM population founders grown in the field (July 30, 2024). (a) Comparison of rates of linear electron flow (LEFamb) against ambient PAR (PARamb). (b) Rates of LEF (LEFhigh) following 10 sec illumination at high light, compared to ambient PAR. (c) The difference between rates of LEF under ambient and high light, compared to ambient PAR. (d) Comparison of phiPSII measured under ambient and high light (yellow symbols), versus ambient PAR (gray symbols). Figure S4. Comparison of NPQt values for the SoyNAM founders on July 23, 2024. (a) Boxplot comparing NPQt values recorded for SoyNAM founders under high light. Values represent the mean of three technical (individual plant) replicates per plot (n = 5). (b) Comparison of NPQt measured under ambient light (PARamb) and NPQt (NPQtamb), individual technical replicates are shown. (c) Comparison of NPQt measured under high light (NPQthigh) and ambient PAR. Individual technical replicates are shown. Figure S5. Comparison of NPQt values for the SoyNAM founders on July 30, 2024. (a) Boxplot comparing NPQt values recorded for SoyNAM founder [file TPJ-121-0-s012.zip › Figure_S5_NAM_NPQt_24_07_30.pdf]

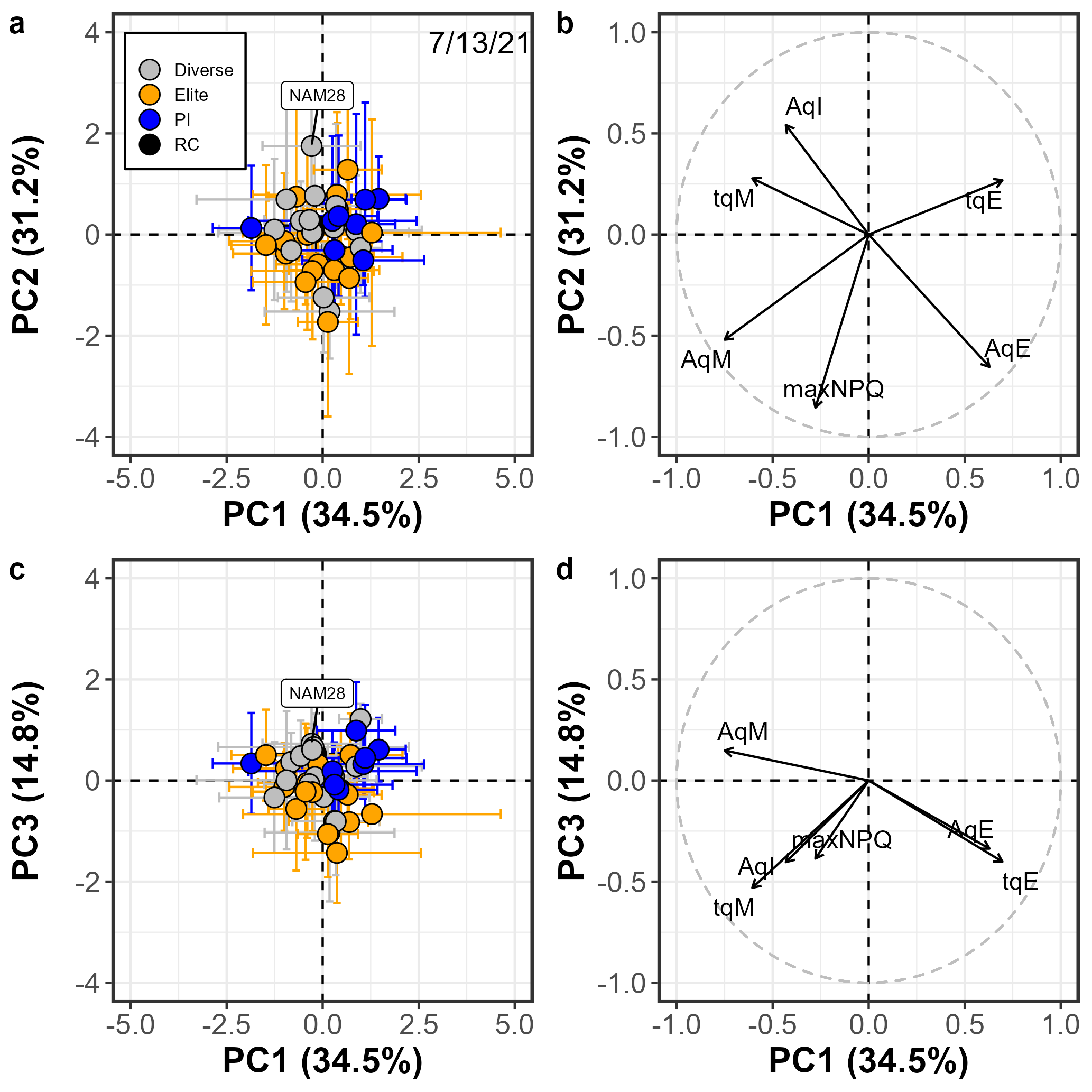

Supplement: Supplementary file 3 — Figure S1. Comparison of genotypic means for NPQ relaxation parameters measured in 2021 and 2022 using the SoyNAM founders. Scatterplots comparing values of (a) maximum inducible NPQ, (b) A qE, (c) A qM, (d) A qI, (e) τ qE, and (f) τ qM. Values represent the mean of seasonal measurements. Pearson correlation coefficient (R) and P‐value are reported for each parameter. Figure S2. Direct measurement of NPQ in NAM population founders grown in the field (July 23, 2024). (a) Comparison of rates of linear electron flow (LEFamb) against ambient PAR (PARamb). (b) Rates of LEF (LEFhigh) following 10 sec illumination at high light, compared to ambient PAR. (c) The difference between rates of LEF under ambient and high light, compared to ambient PAR. (d) Comparison of phiPSII measured under ambient and high light (yellow symbols), versus ambient PAR (gray symbols). Figure S3. Direct measurement of NPQ in NAM population founders grown in the field (July 30, 2024). (a) Comparison of rates of linear electron flow (LEFamb) against ambient PAR (PARamb). (b) Rates of LEF (LEFhigh) following 10 sec illumination at high light, compared to ambient PAR. (c) The difference between rates of LEF under ambient and high light, compared to ambient PAR. (d) Comparison of phiPSII measured under ambient and high light (yellow symbols), versus ambient PAR (gray symbols). Figure S4. Comparison of NPQt values for the SoyNAM founders on July 23, 2024. (a) Boxplot comparing NPQt values recorded for SoyNAM founders under high light. Values represent the mean of three technical (individual plant) replicates per plot (n = 5). (b) Comparison of NPQt measured under ambient light (PARamb) and NPQt (NPQtamb), individual technical replicates are shown. (c) Comparison of NPQt measured under high light (NPQthigh) and ambient PAR. Individual technical replicates are shown. Figure S5. Comparison of NPQt values for the SoyNAM founders on July 30, 2024. (a) Boxplot comparing NPQt values recorded for SoyNAM founder [file TPJ-121-0-s012.zip › Figure_S5_PCA_day71321.tiff]

**a**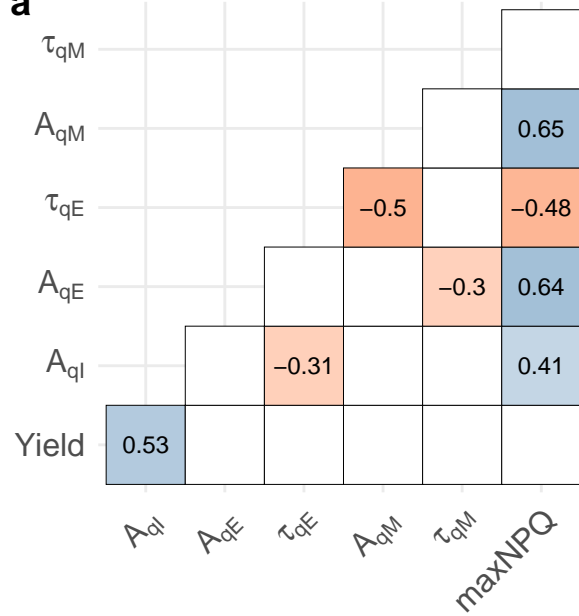**b**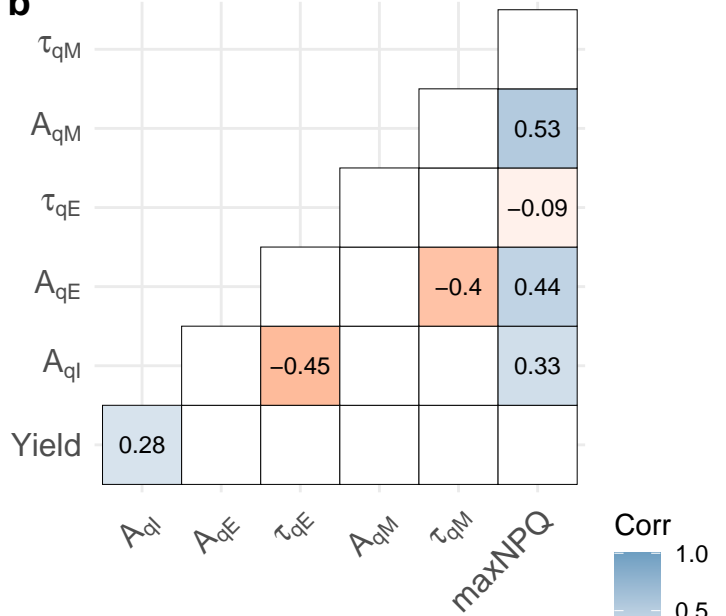**c**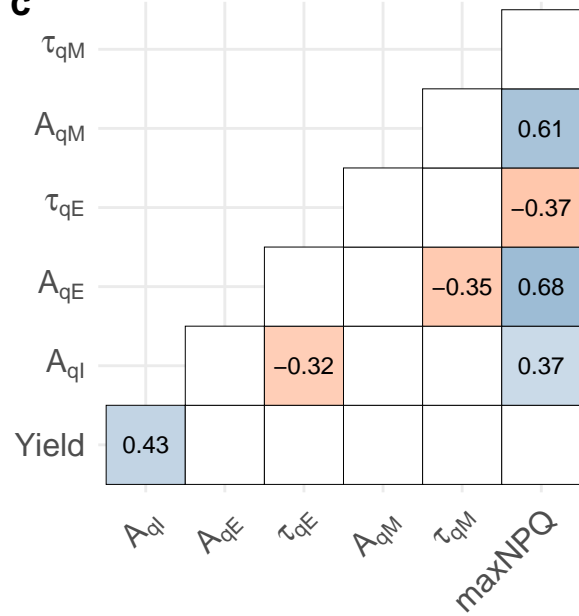

Supplement: Supplementary file 3 — Figure S1. Comparison of genotypic means for NPQ relaxation parameters measured in 2021 and 2022 using the SoyNAM founders. Scatterplots comparing values of (a) maximum inducible NPQ, (b) A qE, (c) A qM, (d) A qI, (e) τ qE, and (f) τ qM. Values represent the mean of seasonal measurements. Pearson correlation coefficient (R) and P‐value are reported for each parameter. Figure S2. Direct measurement of NPQ in NAM population founders grown in the field (July 23, 2024). (a) Comparison of rates of linear electron flow (LEFamb) against ambient PAR (PARamb). (b) Rates of LEF (LEFhigh) following 10 sec illumination at high light, compared to ambient PAR. (c) The difference between rates of LEF under ambient and high light, compared to ambient PAR. (d) Comparison of phiPSII measured under ambient and high light (yellow symbols), versus ambient PAR (gray symbols). Figure S3. Direct measurement of NPQ in NAM population founders grown in the field (July 30, 2024). (a) Comparison of rates of linear electron flow (LEFamb) against ambient PAR (PARamb). (b) Rates of LEF (LEFhigh) following 10 sec illumination at high light, compared to ambient PAR. (c) The difference between rates of LEF under ambient and high light, compared to ambient PAR. (d) Comparison of phiPSII measured under ambient and high light (yellow symbols), versus ambient PAR (gray symbols). Figure S4. Comparison of NPQt values for the SoyNAM founders on July 23, 2024. (a) Boxplot comparing NPQt values recorded for SoyNAM founders under high light. Values represent the mean of three technical (individual plant) replicates per plot (n = 5). (b) Comparison of NPQt measured under ambient light (PARamb) and NPQt (NPQtamb), individual technical replicates are shown. (c) Comparison of NPQt measured under high light (NPQthigh) and ambient PAR. Individual technical replicates are shown. Figure S5. Comparison of NPQt values for the SoyNAM founders on July 30, 2024. (a) Boxplot comparing NPQt values recorded for SoyNAM founder [file TPJ-121-0-s012.zip › Figure_S6_NAM_param_coeff.pdf]

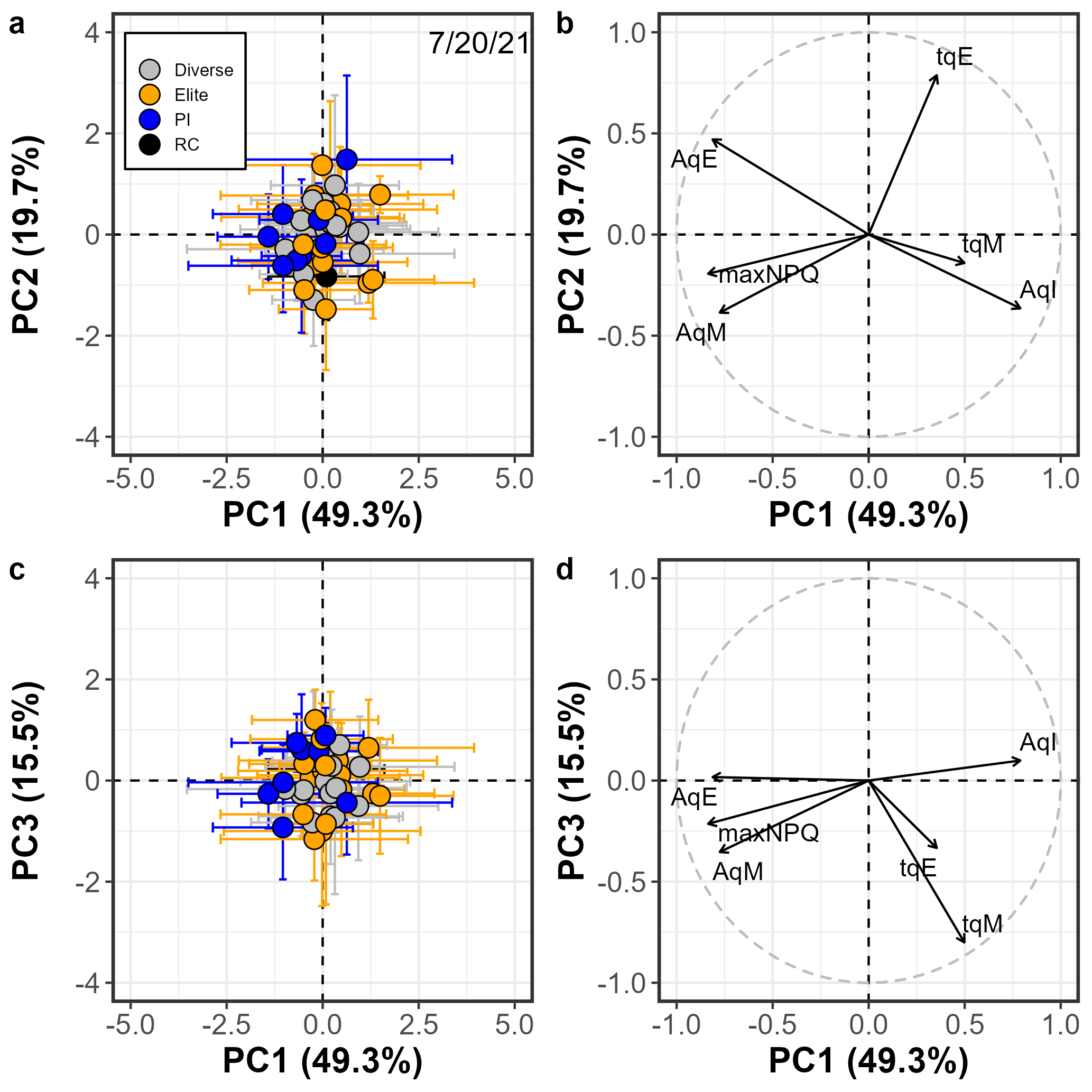

Supplement: Supplementary file 3 — Figure S1. Comparison of genotypic means for NPQ relaxation parameters measured in 2021 and 2022 using the SoyNAM founders. Scatterplots comparing values of (a) maximum inducible NPQ, (b) A qE, (c) A qM, (d) A qI, (e) τ qE, and (f) τ qM. Values represent the mean of seasonal measurements. Pearson correlation coefficient (R) and P‐value are reported for each parameter. Figure S2. Direct measurement of NPQ in NAM population founders grown in the field (July 23, 2024). (a) Comparison of rates of linear electron flow (LEFamb) against ambient PAR (PARamb). (b) Rates of LEF (LEFhigh) following 10 sec illumination at high light, compared to ambient PAR. (c) The difference between rates of LEF under ambient and high light, compared to ambient PAR. (d) Comparison of phiPSII measured under ambient and high light (yellow symbols), versus ambient PAR (gray symbols). Figure S3. Direct measurement of NPQ in NAM population founders grown in the field (July 30, 2024). (a) Comparison of rates of linear electron flow (LEFamb) against ambient PAR (PARamb). (b) Rates of LEF (LEFhigh) following 10 sec illumination at high light, compared to ambient PAR. (c) The difference between rates of LEF under ambient and high light, compared to ambient PAR. (d) Comparison of phiPSII measured under ambient and high light (yellow symbols), versus ambient PAR (gray symbols). Figure S4. Comparison of NPQt values for the SoyNAM founders on July 23, 2024. (a) Boxplot comparing NPQt values recorded for SoyNAM founders under high light. Values represent the mean of three technical (individual plant) replicates per plot (n = 5). (b) Comparison of NPQt measured under ambient light (PARamb) and NPQt (NPQtamb), individual technical replicates are shown. (c) Comparison of NPQt measured under high light (NPQthigh) and ambient PAR. Individual technical replicates are shown. Figure S5. Comparison of NPQt values for the SoyNAM founders on July 30, 2024. (a) Boxplot comparing NPQt values recorded for SoyNAM founder [file TPJ-121-0-s012.zip › Figure_S6_PCA_day72021.tiff]

**a**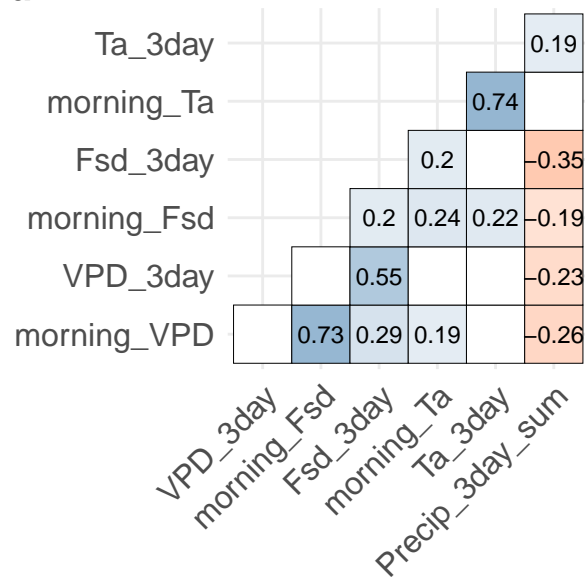**b**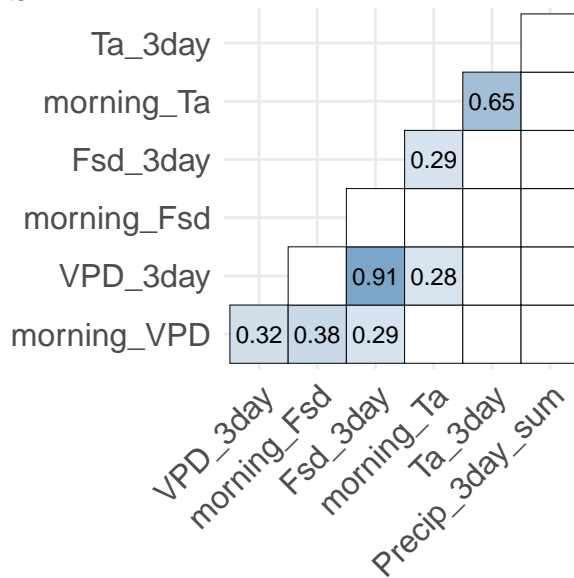**c**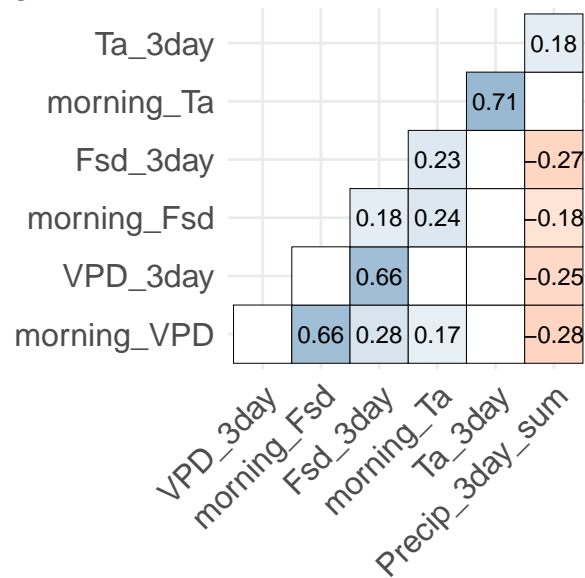

Corr

1.0

0.5

0.0

-0.5

-1.0

Supplement: Supplementary file 3 — Figure S1. Comparison of genotypic means for NPQ relaxation parameters measured in 2021 and 2022 using the SoyNAM founders. Scatterplots comparing values of (a) maximum inducible NPQ, (b) A qE, (c) A qM, (d) A qI, (e) τ qE, and (f) τ qM. Values represent the mean of seasonal measurements. Pearson correlation coefficient (R) and P‐value are reported for each parameter. Figure S2. Direct measurement of NPQ in NAM population founders grown in the field (July 23, 2024). (a) Comparison of rates of linear electron flow (LEFamb) against ambient PAR (PARamb). (b) Rates of LEF (LEFhigh) following 10 sec illumination at high light, compared to ambient PAR. (c) The difference between rates of LEF under ambient and high light, compared to ambient PAR. (d) Comparison of phiPSII measured under ambient and high light (yellow symbols), versus ambient PAR (gray symbols). Figure S3. Direct measurement of NPQ in NAM population founders grown in the field (July 30, 2024). (a) Comparison of rates of linear electron flow (LEFamb) against ambient PAR (PARamb). (b) Rates of LEF (LEFhigh) following 10 sec illumination at high light, compared to ambient PAR. (c) The difference between rates of LEF under ambient and high light, compared to ambient PAR. (d) Comparison of phiPSII measured under ambient and high light (yellow symbols), versus ambient PAR (gray symbols). Figure S4. Comparison of NPQt values for the SoyNAM founders on July 23, 2024. (a) Boxplot comparing NPQt values recorded for SoyNAM founders under high light. Values represent the mean of three technical (individual plant) replicates per plot (n = 5). (b) Comparison of NPQt measured under ambient light (PARamb) and NPQt (NPQtamb), individual technical replicates are shown. (c) Comparison of NPQt measured under high light (NPQthigh) and ambient PAR. Individual technical replicates are shown. Figure S5. Comparison of NPQt values for the SoyNAM founders on July 30, 2024. (a) Boxplot comparing NPQt values recorded for SoyNAM founder [file TPJ-121-0-s012.zip › Figure_S7_NAM_weather_coeff.pdf]

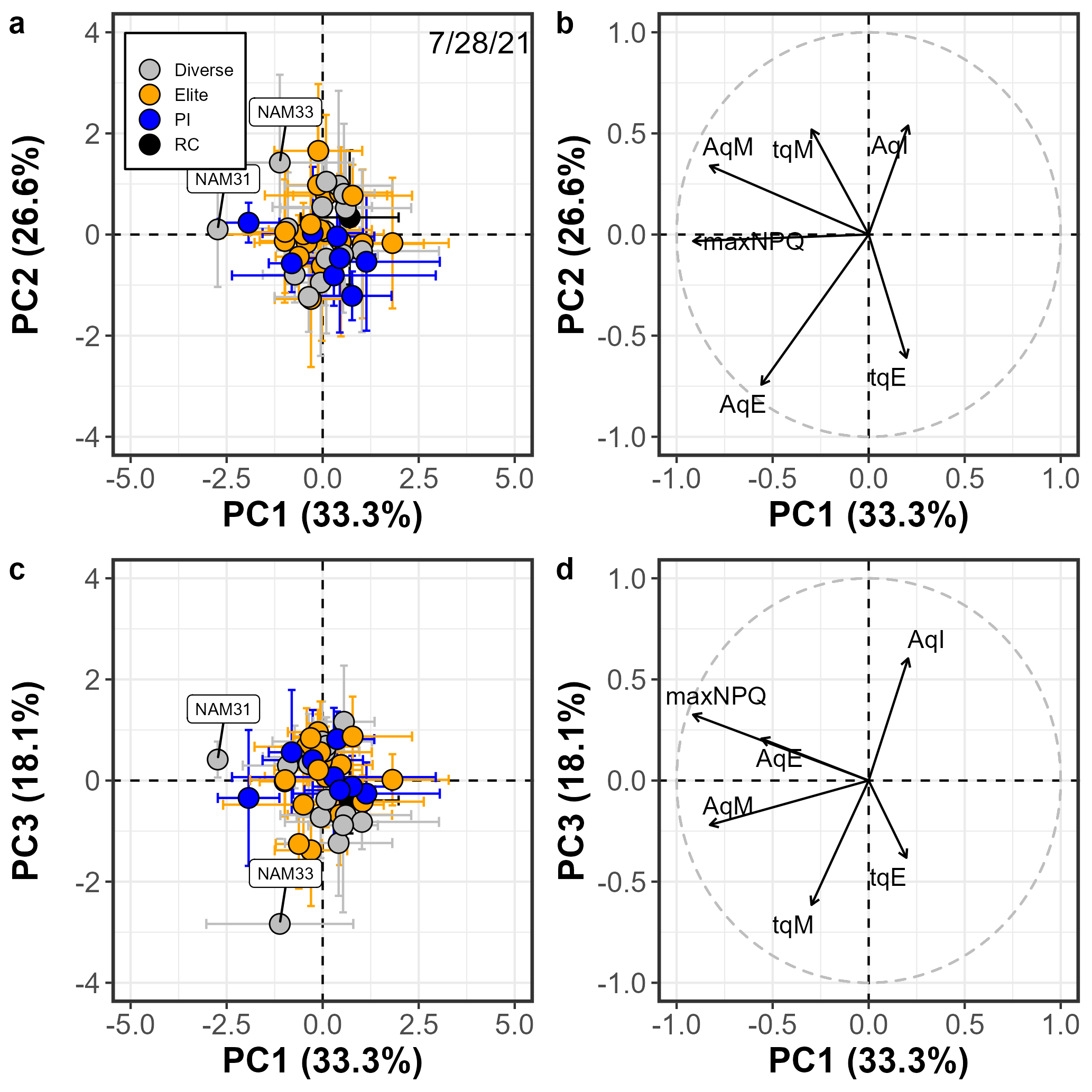

Supplement: Supplementary file 3 — Figure S1. Comparison of genotypic means for NPQ relaxation parameters measured in 2021 and 2022 using the SoyNAM founders. Scatterplots comparing values of (a) maximum inducible NPQ, (b) A qE, (c) A qM, (d) A qI, (e) τ qE, and (f) τ qM. Values represent the mean of seasonal measurements. Pearson correlation coefficient (R) and P‐value are reported for each parameter. Figure S2. Direct measurement of NPQ in NAM population founders grown in the field (July 23, 2024). (a) Comparison of rates of linear electron flow (LEFamb) against ambient PAR (PARamb). (b) Rates of LEF (LEFhigh) following 10 sec illumination at high light, compared to ambient PAR. (c) The difference between rates of LEF under ambient and high light, compared to ambient PAR. (d) Comparison of phiPSII measured under ambient and high light (yellow symbols), versus ambient PAR (gray symbols). Figure S3. Direct measurement of NPQ in NAM population founders grown in the field (July 30, 2024). (a) Comparison of rates of linear electron flow (LEFamb) against ambient PAR (PARamb). (b) Rates of LEF (LEFhigh) following 10 sec illumination at high light, compared to ambient PAR. (c) The difference between rates of LEF under ambient and high light, compared to ambient PAR. (d) Comparison of phiPSII measured under ambient and high light (yellow symbols), versus ambient PAR (gray symbols). Figure S4. Comparison of NPQt values for the SoyNAM founders on July 23, 2024. (a) Boxplot comparing NPQt values recorded for SoyNAM founders under high light. Values represent the mean of three technical (individual plant) replicates per plot (n = 5). (b) Comparison of NPQt measured under ambient light (PARamb) and NPQt (NPQtamb), individual technical replicates are shown. (c) Comparison of NPQt measured under high light (NPQthigh) and ambient PAR. Individual technical replicates are shown. Figure S5. Comparison of NPQt values for the SoyNAM founders on July 30, 2024. (a) Boxplot comparing NPQt values recorded for SoyNAM founder [file TPJ-121-0-s012.zip › Figure_S7_PCA_day72821.tiff]

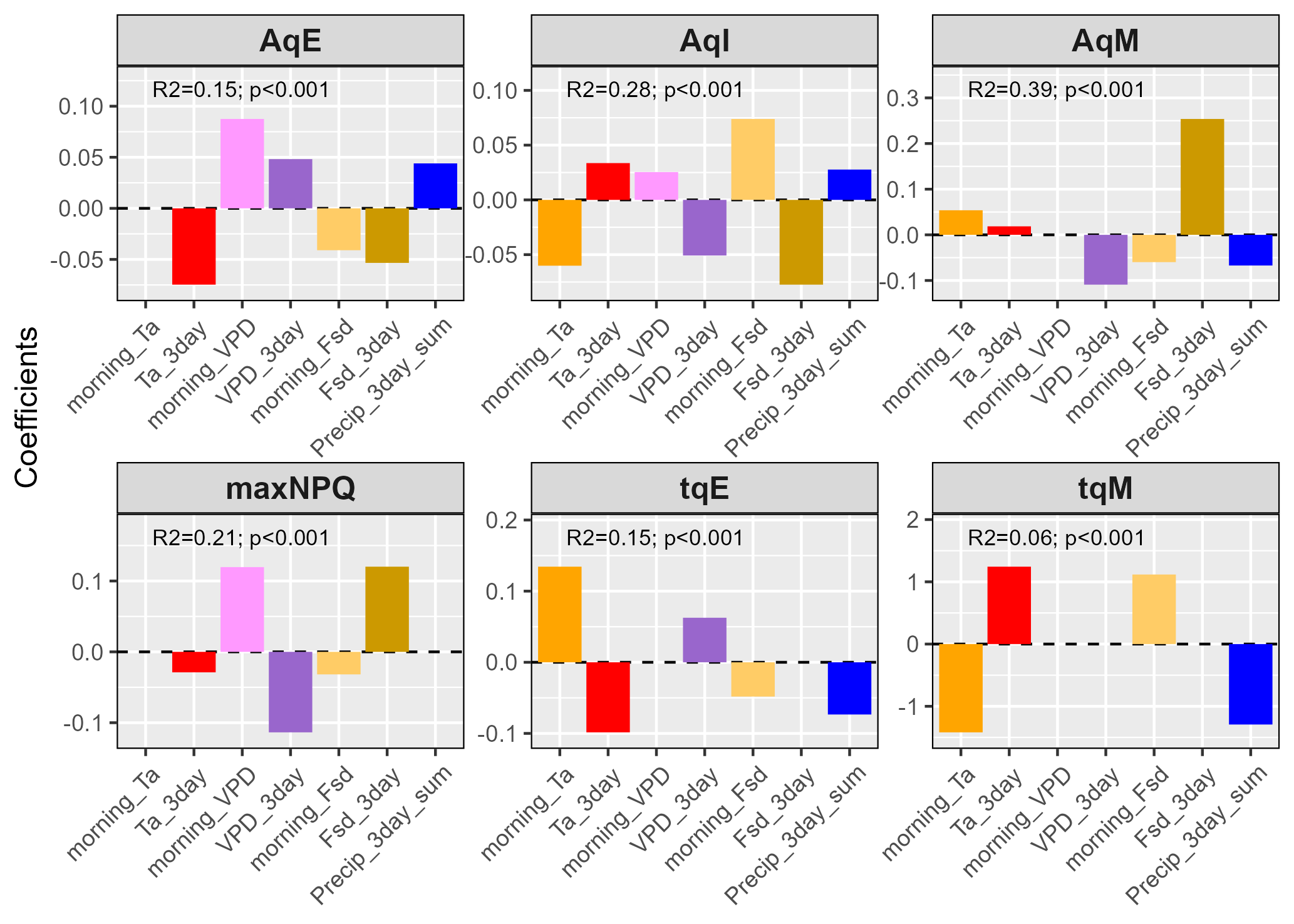

Supplement: Supplementary file 3 — Figure S1. Comparison of genotypic means for NPQ relaxation parameters measured in 2021 and 2022 using the SoyNAM founders. Scatterplots comparing values of (a) maximum inducible NPQ, (b) A qE, (c) A qM, (d) A qI, (e) τ qE, and (f) τ qM. Values represent the mean of seasonal measurements. Pearson correlation coefficient (R) and P‐value are reported for each parameter. Figure S2. Direct measurement of NPQ in NAM population founders grown in the field (July 23, 2024). (a) Comparison of rates of linear electron flow (LEFamb) against ambient PAR (PARamb). (b) Rates of LEF (LEFhigh) following 10 sec illumination at high light, compared to ambient PAR. (c) The difference between rates of LEF under ambient and high light, compared to ambient PAR. (d) Comparison of phiPSII measured under ambient and high light (yellow symbols), versus ambient PAR (gray symbols). Figure S3. Direct measurement of NPQ in NAM population founders grown in the field (July 30, 2024). (a) Comparison of rates of linear electron flow (LEFamb) against ambient PAR (PARamb). (b) Rates of LEF (LEFhigh) following 10 sec illumination at high light, compared to ambient PAR. (c) The difference between rates of LEF under ambient and high light, compared to ambient PAR. (d) Comparison of phiPSII measured under ambient and high light (yellow symbols), versus ambient PAR (gray symbols). Figure S4. Comparison of NPQt values for the SoyNAM founders on July 23, 2024. (a) Boxplot comparing NPQt values recorded for SoyNAM founders under high light. Values represent the mean of three technical (individual plant) replicates per plot (n = 5). (b) Comparison of NPQt measured under ambient light (PARamb) and NPQt (NPQtamb), individual technical replicates are shown. (c) Comparison of NPQt measured under high light (NPQthigh) and ambient PAR. Individual technical replicates are shown. Figure S5. Comparison of NPQt values for the SoyNAM founders on July 30, 2024. (a) Boxplot comparing NPQt values recorded for SoyNAM founder [file TPJ-121-0-s012.zip › Figure_S8_AIC_coef_2years.tiff]

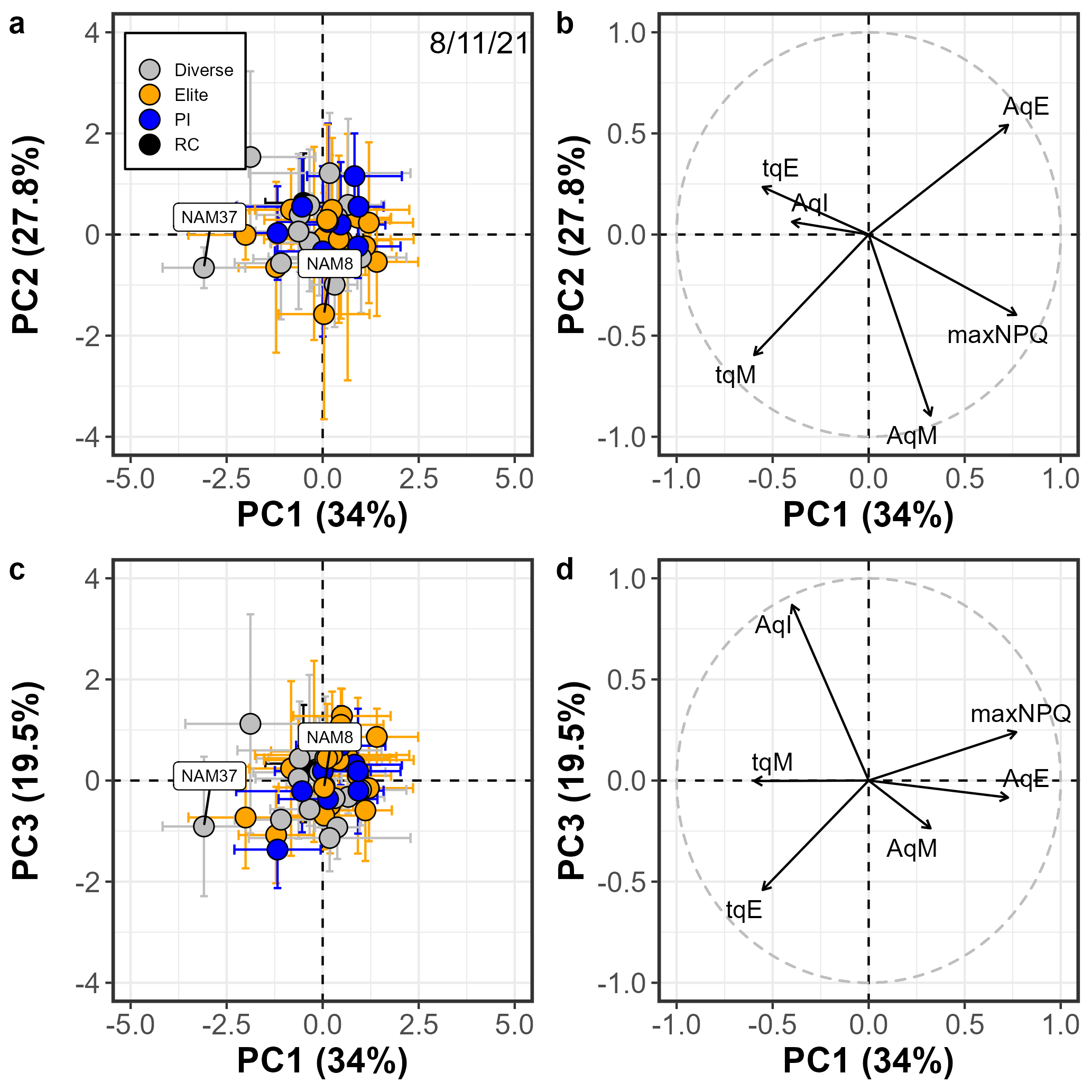

Supplement: Supplementary file 3 — Figure S1. Comparison of genotypic means for NPQ relaxation parameters measured in 2021 and 2022 using the SoyNAM founders. Scatterplots comparing values of (a) maximum inducible NPQ, (b) A qE, (c) A qM, (d) A qI, (e) τ qE, and (f) τ qM. Values represent the mean of seasonal measurements. Pearson correlation coefficient (R) and P‐value are reported for each parameter. Figure S2. Direct measurement of NPQ in NAM population founders grown in the field (July 23, 2024). (a) Comparison of rates of linear electron flow (LEFamb) against ambient PAR (PARamb). (b) Rates of LEF (LEFhigh) following 10 sec illumination at high light, compared to ambient PAR. (c) The difference between rates of LEF under ambient and high light, compared to ambient PAR. (d) Comparison of phiPSII measured under ambient and high light (yellow symbols), versus ambient PAR (gray symbols). Figure S3. Direct measurement of NPQ in NAM population founders grown in the field (July 30, 2024). (a) Comparison of rates of linear electron flow (LEFamb) against ambient PAR (PARamb). (b) Rates of LEF (LEFhigh) following 10 sec illumination at high light, compared to ambient PAR. (c) The difference between rates of LEF under ambient and high light, compared to ambient PAR. (d) Comparison of phiPSII measured under ambient and high light (yellow symbols), versus ambient PAR (gray symbols). Figure S4. Comparison of NPQt values for the SoyNAM founders on July 23, 2024. (a) Boxplot comparing NPQt values recorded for SoyNAM founders under high light. Values represent the mean of three technical (individual plant) replicates per plot (n = 5). (b) Comparison of NPQt measured under ambient light (PARamb) and NPQt (NPQtamb), individual technical replicates are shown. (c) Comparison of NPQt measured under high light (NPQthigh) and ambient PAR. Individual technical replicates are shown. Figure S5. Comparison of NPQt values for the SoyNAM founders on July 30, 2024. (a) Boxplot comparing NPQt values recorded for SoyNAM founder [file TPJ-121-0-s012.zip › Figure_S8_PCA_day81121.tiff]

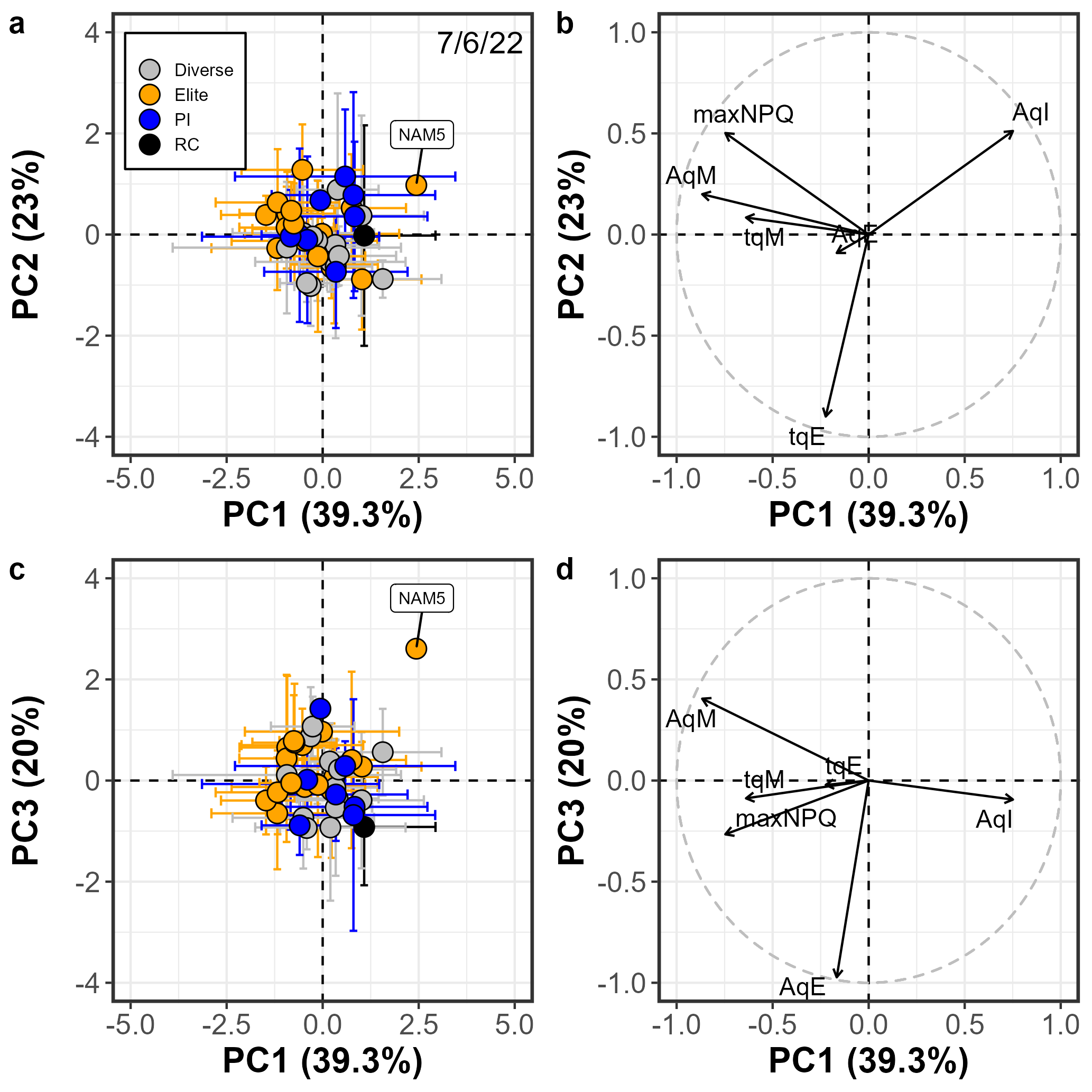

Supplement: Supplementary file 3 — Figure S1. Comparison of genotypic means for NPQ relaxation parameters measured in 2021 and 2022 using the SoyNAM founders. Scatterplots comparing values of (a) maximum inducible NPQ, (b) A qE, (c) A qM, (d) A qI, (e) τ qE, and (f) τ qM. Values represent the mean of seasonal measurements. Pearson correlation coefficient (R) and P‐value are reported for each parameter. Figure S2. Direct measurement of NPQ in NAM population founders grown in the field (July 23, 2024). (a) Comparison of rates of linear electron flow (LEFamb) against ambient PAR (PARamb). (b) Rates of LEF (LEFhigh) following 10 sec illumination at high light, compared to ambient PAR. (c) The difference between rates of LEF under ambient and high light, compared to ambient PAR. (d) Comparison of phiPSII measured under ambient and high light (yellow symbols), versus ambient PAR (gray symbols). Figure S3. Direct measurement of NPQ in NAM population founders grown in the field (July 30, 2024). (a) Comparison of rates of linear electron flow (LEFamb) against ambient PAR (PARamb). (b) Rates of LEF (LEFhigh) following 10 sec illumination at high light, compared to ambient PAR. (c) The difference between rates of LEF under ambient and high light, compared to ambient PAR. (d) Comparison of phiPSII measured under ambient and high light (yellow symbols), versus ambient PAR (gray symbols). Figure S4. Comparison of NPQt values for the SoyNAM founders on July 23, 2024. (a) Boxplot comparing NPQt values recorded for SoyNAM founders under high light. Values represent the mean of three technical (individual plant) replicates per plot (n = 5). (b) Comparison of NPQt measured under ambient light (PARamb) and NPQt (NPQtamb), individual technical replicates are shown. (c) Comparison of NPQt measured under high light (NPQthigh) and ambient PAR. Individual technical replicates are shown. Figure S5. Comparison of NPQt values for the SoyNAM founders on July 30, 2024. (a) Boxplot comparing NPQt values recorded for SoyNAM founder [file TPJ-121-0-s012.zip › Figure_S9_PCA_day7622.tiff]

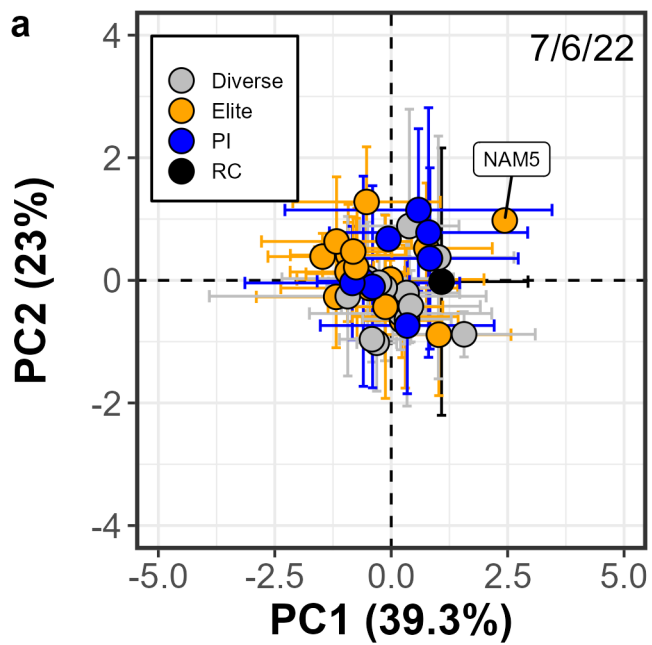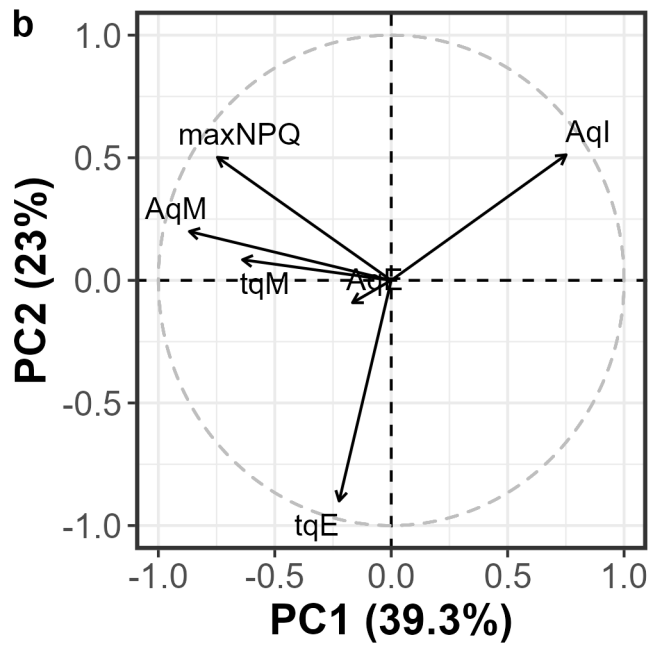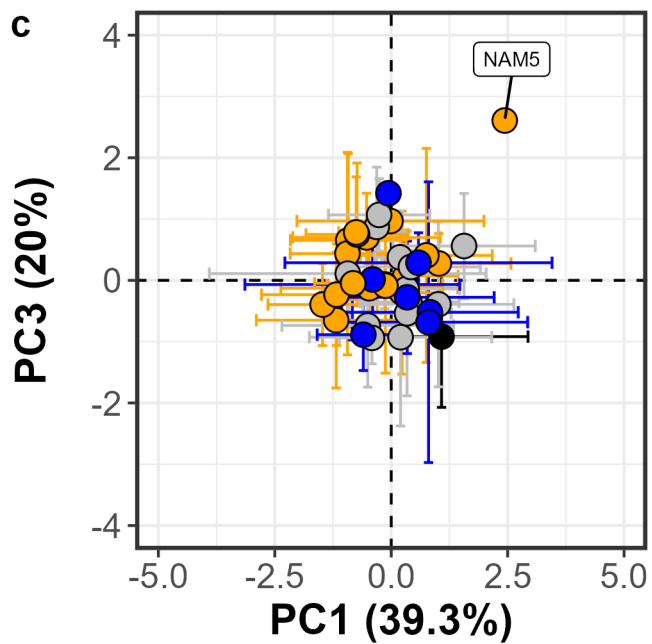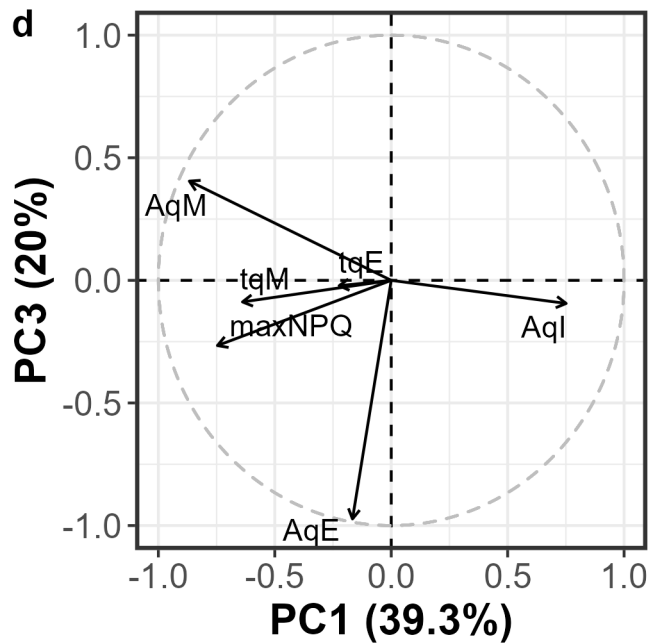

Supplement: Supplementary file 3 — Figure S1. Comparison of genotypic means for NPQ relaxation parameters measured in 2021 and 2022 using the SoyNAM founders. Scatterplots comparing values of (a) maximum inducible NPQ, (b) A qE, (c) A qM, (d) A qI, (e) τ qE, and (f) τ qM. Values represent the mean of seasonal measurements. Pearson correlation coefficient (R) and P‐value are reported for each parameter. Figure S2. Direct measurement of NPQ in NAM population founders grown in the field (July 23, 2024). (a) Comparison of rates of linear electron flow (LEFamb) against ambient PAR (PARamb). (b) Rates of LEF (LEFhigh) following 10 sec illumination at high light, compared to ambient PAR. (c) The difference between rates of LEF under ambient and high light, compared to ambient PAR. (d) Comparison of phiPSII measured under ambient and high light (yellow symbols), versus ambient PAR (gray symbols). Figure S3. Direct measurement of NPQ in NAM population founders grown in the field (July 30, 2024). (a) Comparison of rates of linear electron flow (LEFamb) against ambient PAR (PARamb). (b) Rates of LEF (LEFhigh) following 10 sec illumination at high light, compared to ambient PAR. (c) The difference between rates of LEF under ambient and high light, compared to ambient PAR. (d) Comparison of phiPSII measured under ambient and high light (yellow symbols), versus ambient PAR (gray symbols). Figure S4. Comparison of NPQt values for the SoyNAM founders on July 23, 2024. (a) Boxplot comparing NPQt values recorded for SoyNAM founders under high light. Values represent the mean of three technical (individual plant) replicates per plot (n = 5). (b) Comparison of NPQt measured under ambient light (PARamb) and NPQt (NPQtamb), individual technical replicates are shown. (c) Comparison of NPQt measured under high light (NPQthigh) and ambient PAR. Individual technical replicates are shown. Figure S5. Comparison of NPQt values for the SoyNAM founders on July 30, 2024. (a) Boxplot comparing NPQt values recorded for SoyNAM founder [file TPJ-121-0-s012.zip › 3_Figure_S16_PCA_day7622.pdf]

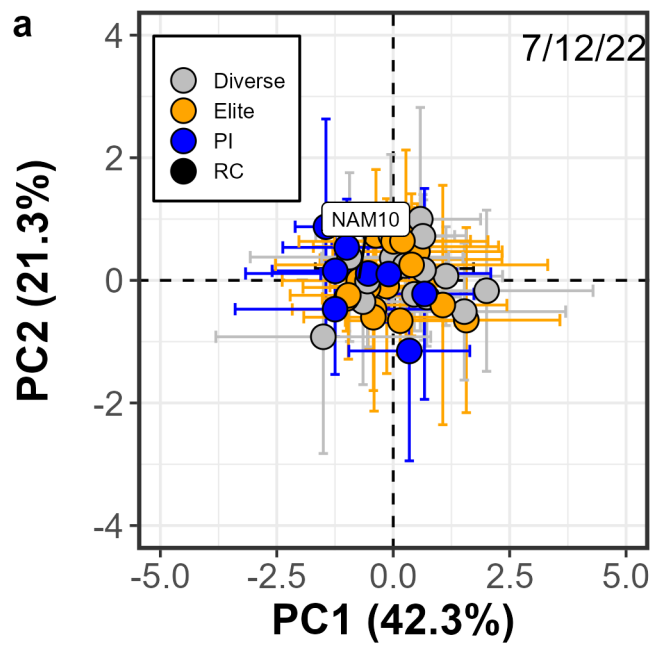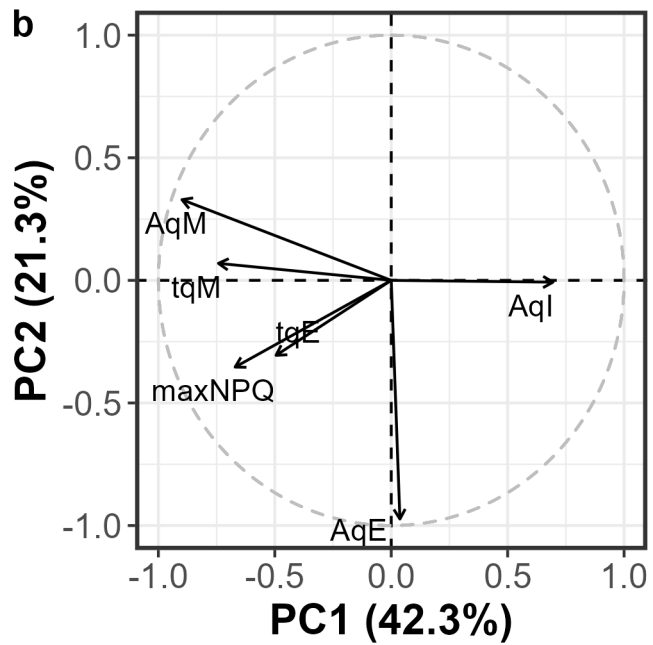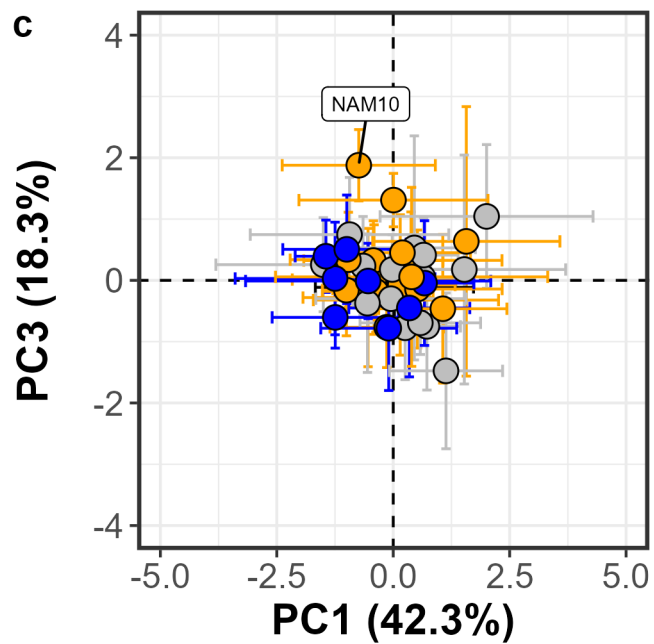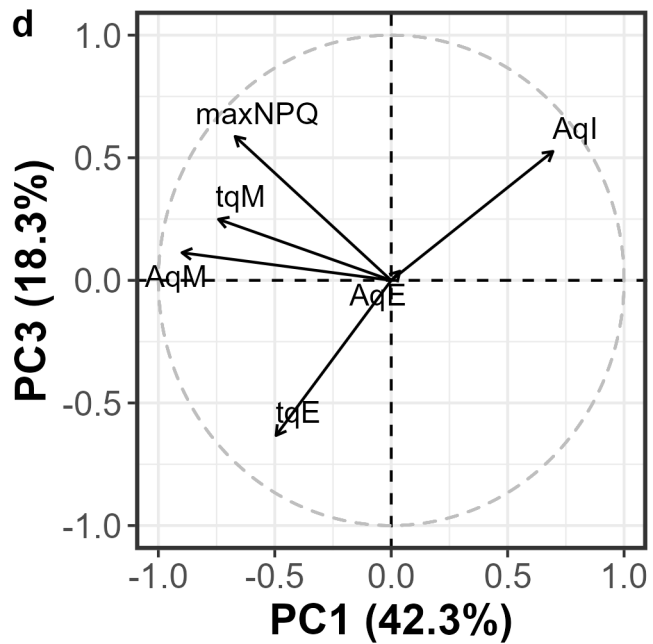

Supplement: Supplementary file 3 — Figure S1. Comparison of genotypic means for NPQ relaxation parameters measured in 2021 and 2022 using the SoyNAM founders. Scatterplots comparing values of (a) maximum inducible NPQ, (b) A qE, (c) A qM, (d) A qI, (e) τ qE, and (f) τ qM. Values represent the mean of seasonal measurements. Pearson correlation coefficient (R) and P‐value are reported for each parameter. Figure S2. Direct measurement of NPQ in NAM population founders grown in the field (July 23, 2024). (a) Comparison of rates of linear electron flow (LEFamb) against ambient PAR (PARamb). (b) Rates of LEF (LEFhigh) following 10 sec illumination at high light, compared to ambient PAR. (c) The difference between rates of LEF under ambient and high light, compared to ambient PAR. (d) Comparison of phiPSII measured under ambient and high light (yellow symbols), versus ambient PAR (gray symbols). Figure S3. Direct measurement of NPQ in NAM population founders grown in the field (July 30, 2024). (a) Comparison of rates of linear electron flow (LEFamb) against ambient PAR (PARamb). (b) Rates of LEF (LEFhigh) following 10 sec illumination at high light, compared to ambient PAR. (c) The difference between rates of LEF under ambient and high light, compared to ambient PAR. (d) Comparison of phiPSII measured under ambient and high light (yellow symbols), versus ambient PAR (gray symbols). Figure S4. Comparison of NPQt values for the SoyNAM founders on July 23, 2024. (a) Boxplot comparing NPQt values recorded for SoyNAM founders under high light. Values represent the mean of three technical (individual plant) replicates per plot (n = 5). (b) Comparison of NPQt measured under ambient light (PARamb) and NPQt (NPQtamb), individual technical replicates are shown. (c) Comparison of NPQt measured under high light (NPQthigh) and ambient PAR. Individual technical replicates are shown. Figure S5. Comparison of NPQt values for the SoyNAM founders on July 30, 2024. (a) Boxplot comparing NPQt values recorded for SoyNAM founder [file TPJ-121-0-s012.zip › 3_Figure_S17_PCA_day71222.pdf]

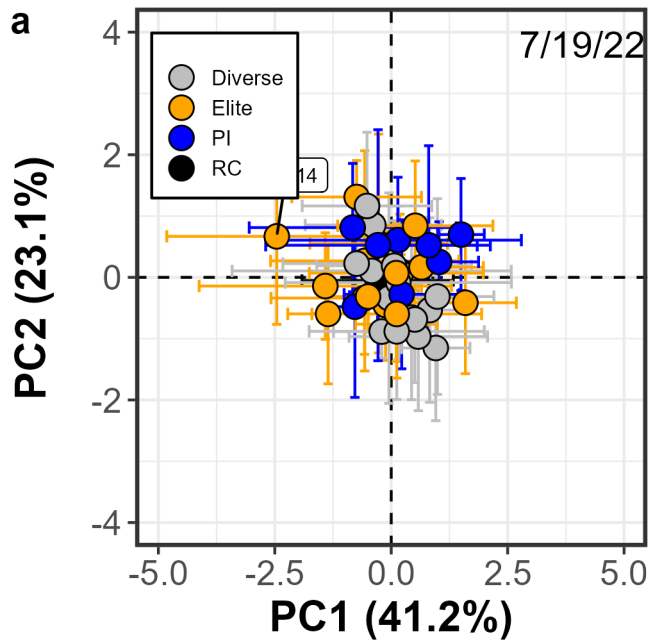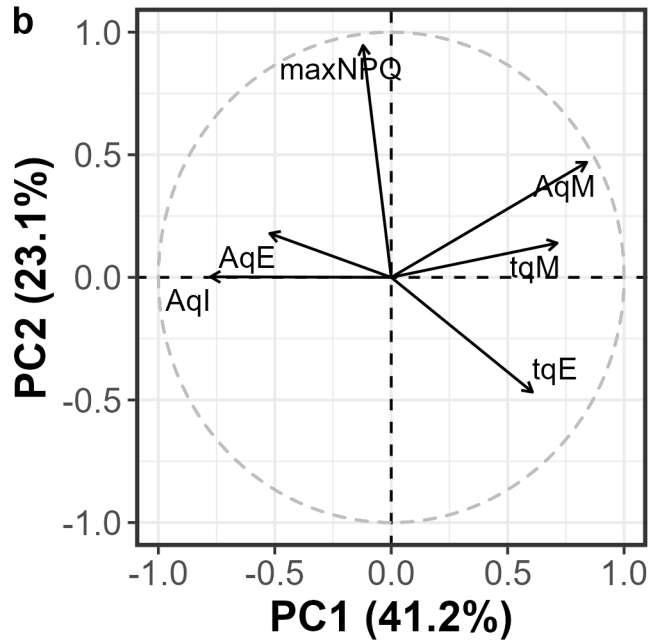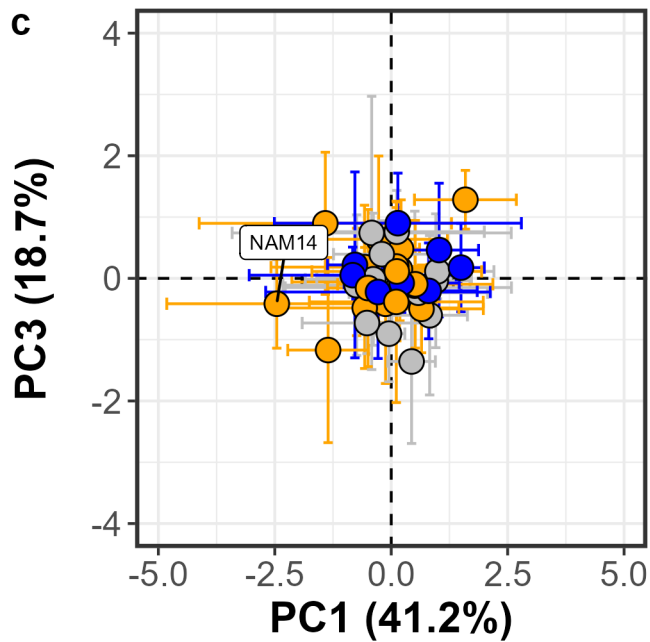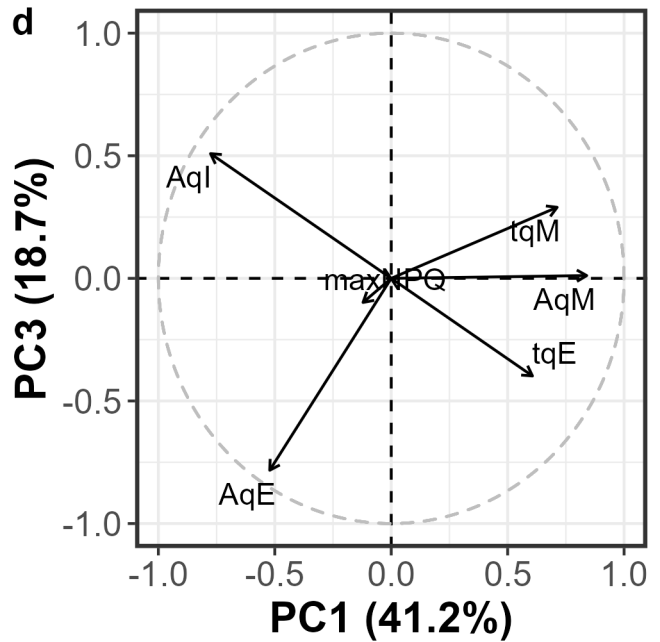

Supplement: Supplementary file 3 — Figure S1. Comparison of genotypic means for NPQ relaxation parameters measured in 2021 and 2022 using the SoyNAM founders. Scatterplots comparing values of (a) maximum inducible NPQ, (b) A qE, (c) A qM, (d) A qI, (e) τ qE, and (f) τ qM. Values represent the mean of seasonal measurements. Pearson correlation coefficient (R) and P‐value are reported for each parameter. Figure S2. Direct measurement of NPQ in NAM population founders grown in the field (July 23, 2024). (a) Comparison of rates of linear electron flow (LEFamb) against ambient PAR (PARamb). (b) Rates of LEF (LEFhigh) following 10 sec illumination at high light, compared to ambient PAR. (c) The difference between rates of LEF under ambient and high light, compared to ambient PAR. (d) Comparison of phiPSII measured under ambient and high light (yellow symbols), versus ambient PAR (gray symbols). Figure S3. Direct measurement of NPQ in NAM population founders grown in the field (July 30, 2024). (a) Comparison of rates of linear electron flow (LEFamb) against ambient PAR (PARamb). (b) Rates of LEF (LEFhigh) following 10 sec illumination at high light, compared to ambient PAR. (c) The difference between rates of LEF under ambient and high light, compared to ambient PAR. (d) Comparison of phiPSII measured under ambient and high light (yellow symbols), versus ambient PAR (gray symbols). Figure S4. Comparison of NPQt values for the SoyNAM founders on July 23, 2024. (a) Boxplot comparing NPQt values recorded for SoyNAM founders under high light. Values represent the mean of three technical (individual plant) replicates per plot (n = 5). (b) Comparison of NPQt measured under ambient light (PARamb) and NPQt (NPQtamb), individual technical replicates are shown. (c) Comparison of NPQt measured under high light (NPQthigh) and ambient PAR. Individual technical replicates are shown. Figure S5. Comparison of NPQt values for the SoyNAM founders on July 30, 2024. (a) Boxplot comparing NPQt values recorded for SoyNAM founder [file TPJ-121-0-s012.zip › 3_Figure_S18_PCA_day71922.pdf]

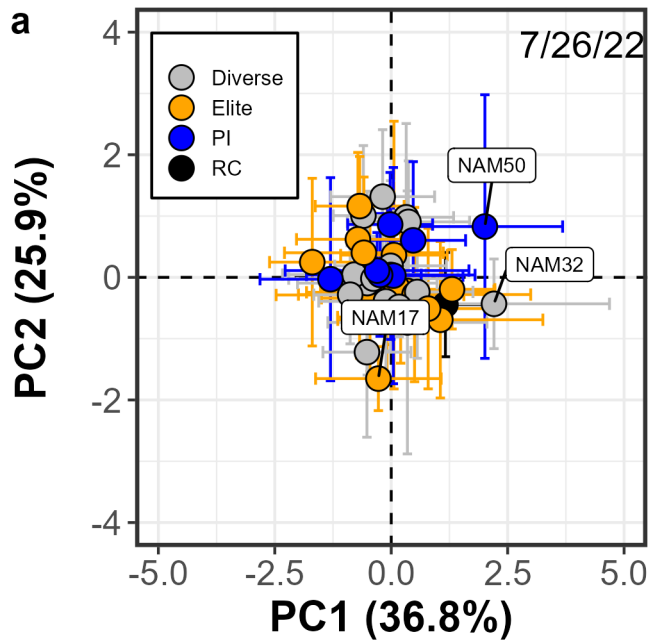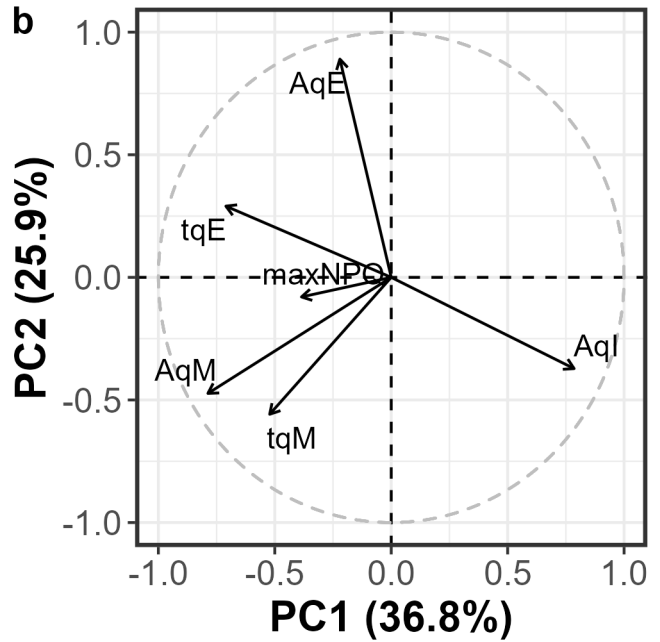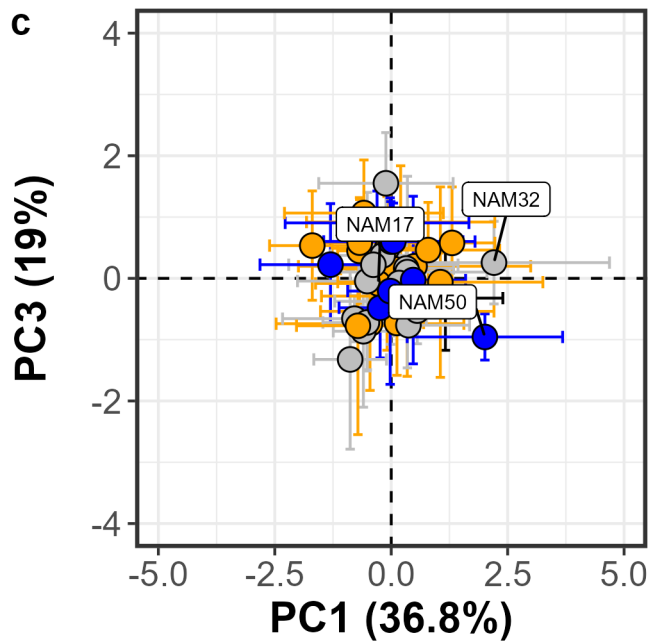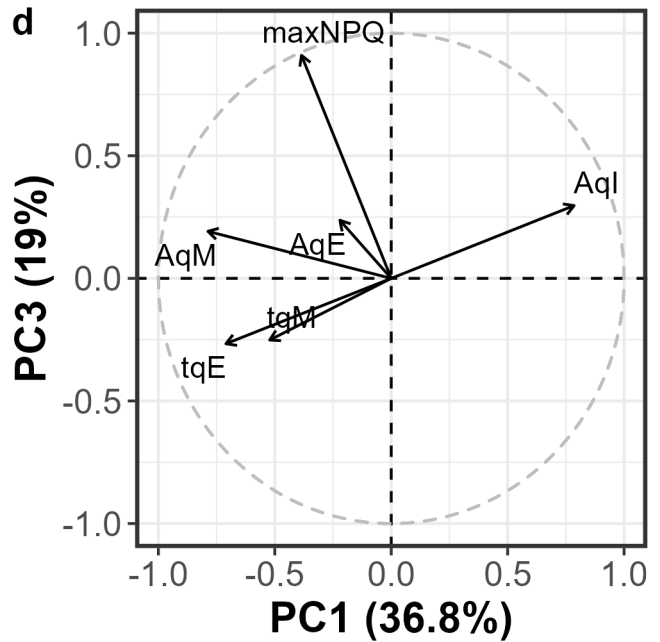

Supplement: Supplementary file 3 — Figure S1. Comparison of genotypic means for NPQ relaxation parameters measured in 2021 and 2022 using the SoyNAM founders. Scatterplots comparing values of (a) maximum inducible NPQ, (b) A qE, (c) A qM, (d) A qI, (e) τ qE, and (f) τ qM. Values represent the mean of seasonal measurements. Pearson correlation coefficient (R) and P‐value are reported for each parameter. Figure S2. Direct measurement of NPQ in NAM population founders grown in the field (July 23, 2024). (a) Comparison of rates of linear electron flow (LEFamb) against ambient PAR (PARamb). (b) Rates of LEF (LEFhigh) following 10 sec illumination at high light, compared to ambient PAR. (c) The difference between rates of LEF under ambient and high light, compared to ambient PAR. (d) Comparison of phiPSII measured under ambient and high light (yellow symbols), versus ambient PAR (gray symbols). Figure S3. Direct measurement of NPQ in NAM population founders grown in the field (July 30, 2024). (a) Comparison of rates of linear electron flow (LEFamb) against ambient PAR (PARamb). (b) Rates of LEF (LEFhigh) following 10 sec illumination at high light, compared to ambient PAR. (c) The difference between rates of LEF under ambient and high light, compared to ambient PAR. (d) Comparison of phiPSII measured under ambient and high light (yellow symbols), versus ambient PAR (gray symbols). Figure S4. Comparison of NPQt values for the SoyNAM founders on July 23, 2024. (a) Boxplot comparing NPQt values recorded for SoyNAM founders under high light. Values represent the mean of three technical (individual plant) replicates per plot (n = 5). (b) Comparison of NPQt measured under ambient light (PARamb) and NPQt (NPQtamb), individual technical replicates are shown. (c) Comparison of NPQt measured under high light (NPQthigh) and ambient PAR. Individual technical replicates are shown. Figure S5. Comparison of NPQt values for the SoyNAM founders on July 30, 2024. (a) Boxplot comparing NPQt values recorded for SoyNAM founder [file TPJ-121-0-s012.zip › 3_Figure_S19_PCA_day72622.pdf]

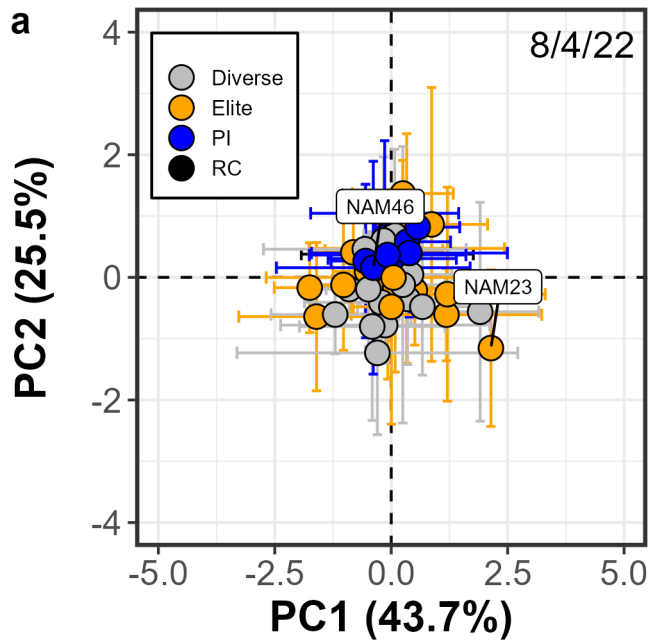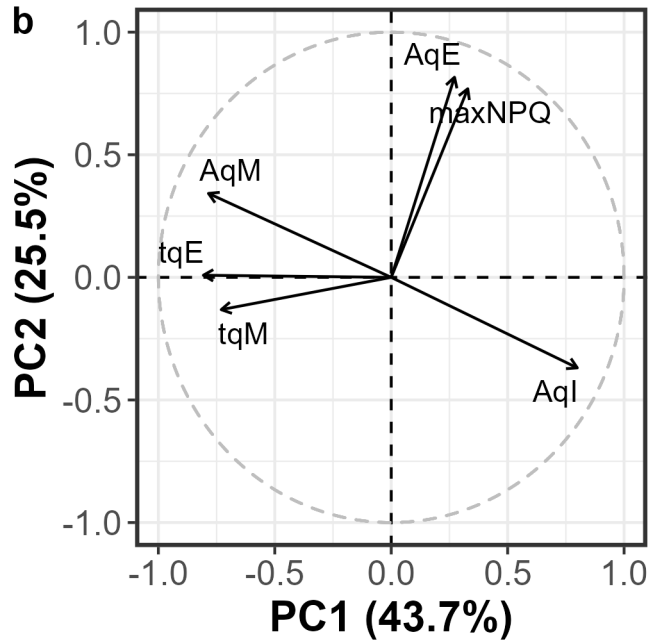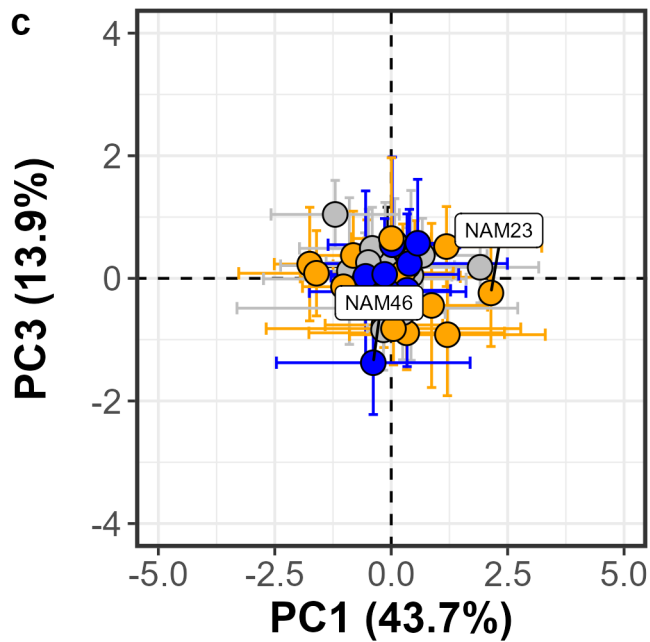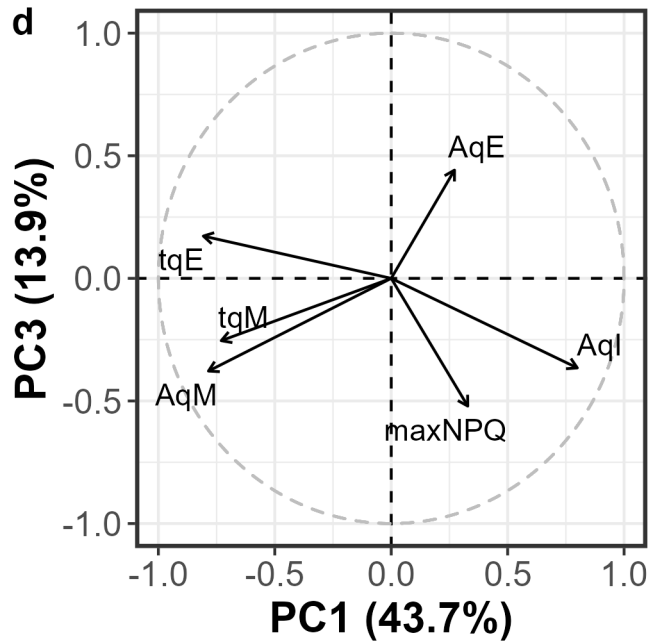

Supplement: Supplementary file 3 — Figure S1. Comparison of genotypic means for NPQ relaxation parameters measured in 2021 and 2022 using the SoyNAM founders. Scatterplots comparing values of (a) maximum inducible NPQ, (b) A qE, (c) A qM, (d) A qI, (e) τ qE, and (f) τ qM. Values represent the mean of seasonal measurements. Pearson correlation coefficient (R) and P‐value are reported for each parameter. Figure S2. Direct measurement of NPQ in NAM population founders grown in the field (July 23, 2024). (a) Comparison of rates of linear electron flow (LEFamb) against ambient PAR (PARamb). (b) Rates of LEF (LEFhigh) following 10 sec illumination at high light, compared to ambient PAR. (c) The difference between rates of LEF under ambient and high light, compared to ambient PAR. (d) Comparison of phiPSII measured under ambient and high light (yellow symbols), versus ambient PAR (gray symbols). Figure S3. Direct measurement of NPQ in NAM population founders grown in the field (July 30, 2024). (a) Comparison of rates of linear electron flow (LEFamb) against ambient PAR (PARamb). (b) Rates of LEF (LEFhigh) following 10 sec illumination at high light, compared to ambient PAR. (c) The difference between rates of LEF under ambient and high light, compared to ambient PAR. (d) Comparison of phiPSII measured under ambient and high light (yellow symbols), versus ambient PAR (gray symbols). Figure S4. Comparison of NPQt values for the SoyNAM founders on July 23, 2024. (a) Boxplot comparing NPQt values recorded for SoyNAM founders under high light. Values represent the mean of three technical (individual plant) replicates per plot (n = 5). (b) Comparison of NPQt measured under ambient light (PARamb) and NPQt (NPQtamb), individual technical replicates are shown. (c) Comparison of NPQt measured under high light (NPQthigh) and ambient PAR. Individual technical replicates are shown. Figure S5. Comparison of NPQt values for the SoyNAM founders on July 30, 2024. (a) Boxplot comparing NPQt values recorded for SoyNAM founder [file TPJ-121-0-s012.zip › 3_Figure_S20_PCA_day8422.pdf]

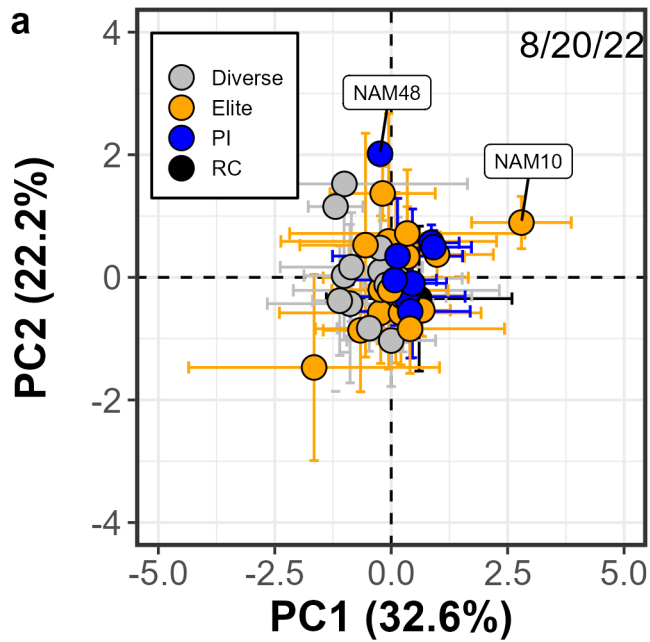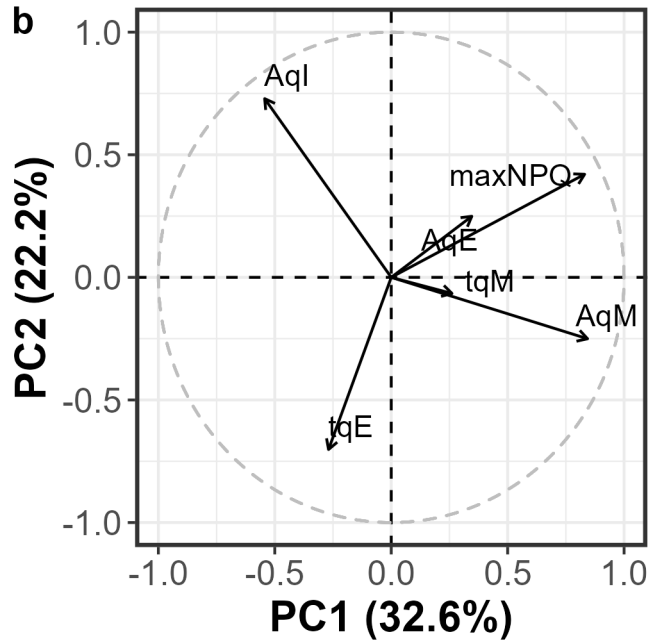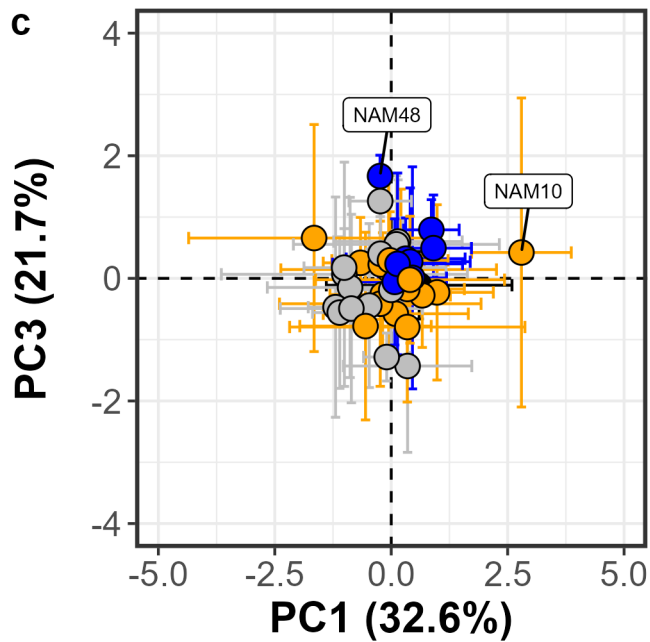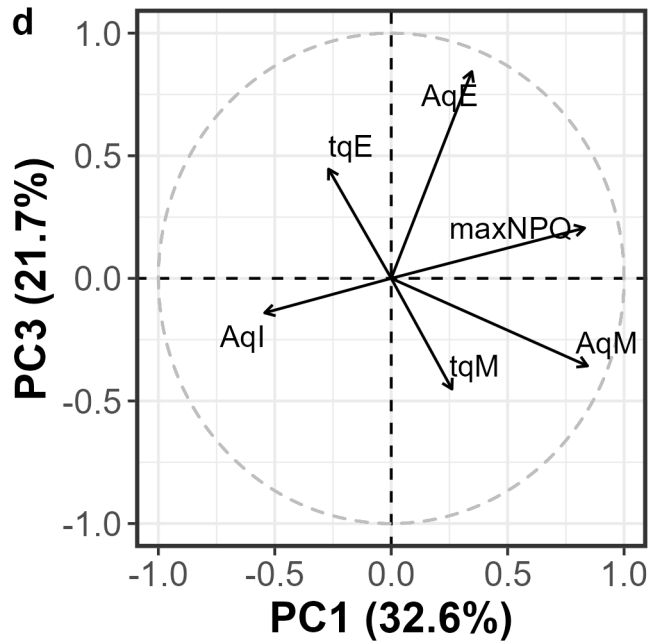

Supplement: Supplementary file 3 — Figure S1. Comparison of genotypic means for NPQ relaxation parameters measured in 2021 and 2022 using the SoyNAM founders. Scatterplots comparing values of (a) maximum inducible NPQ, (b) A qE, (c) A qM, (d) A qI, (e) τ qE, and (f) τ qM. Values represent the mean of seasonal measurements. Pearson correlation coefficient (R) and P‐value are reported for each parameter. Figure S2. Direct measurement of NPQ in NAM population founders grown in the field (July 23, 2024). (a) Comparison of rates of linear electron flow (LEFamb) against ambient PAR (PARamb). (b) Rates of LEF (LEFhigh) following 10 sec illumination at high light, compared to ambient PAR. (c) The difference between rates of LEF under ambient and high light, compared to ambient PAR. (d) Comparison of phiPSII measured under ambient and high light (yellow symbols), versus ambient PAR (gray symbols). Figure S3. Direct measurement of NPQ in NAM population founders grown in the field (July 30, 2024). (a) Comparison of rates of linear electron flow (LEFamb) against ambient PAR (PARamb). (b) Rates of LEF (LEFhigh) following 10 sec illumination at high light, compared to ambient PAR. (c) The difference between rates of LEF under ambient and high light, compared to ambient PAR. (d) Comparison of phiPSII measured under ambient and high light (yellow symbols), versus ambient PAR (gray symbols). Figure S4. Comparison of NPQt values for the SoyNAM founders on July 23, 2024. (a) Boxplot comparing NPQt values recorded for SoyNAM founders under high light. Values represent the mean of three technical (individual plant) replicates per plot (n = 5). (b) Comparison of NPQt measured under ambient light (PARamb) and NPQt (NPQtamb), individual technical replicates are shown. (c) Comparison of NPQt measured under high light (NPQthigh) and ambient PAR. Individual technical replicates are shown. Figure S5. Comparison of NPQt values for the SoyNAM founders on July 30, 2024. (a) Boxplot comparing NPQt values recorded for SoyNAM founder [file TPJ-121-0-s012.zip › 3_Figure_S21_PCA_day82022.pdf]

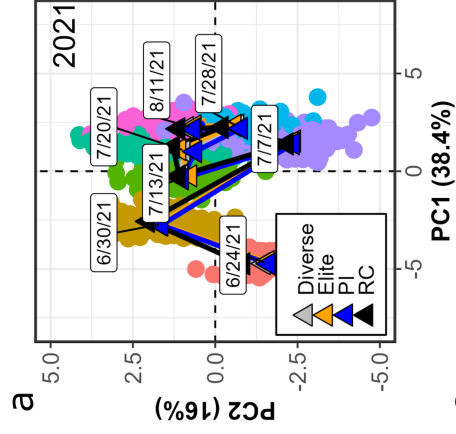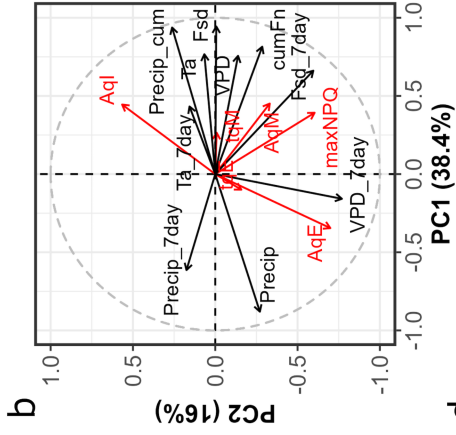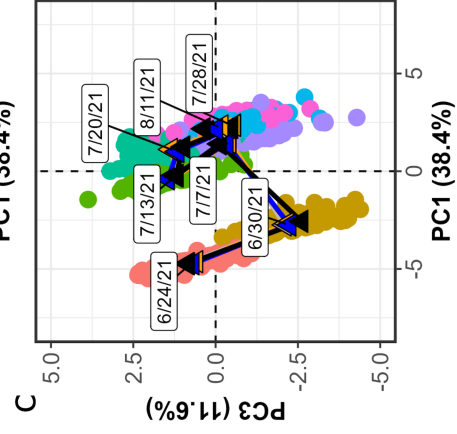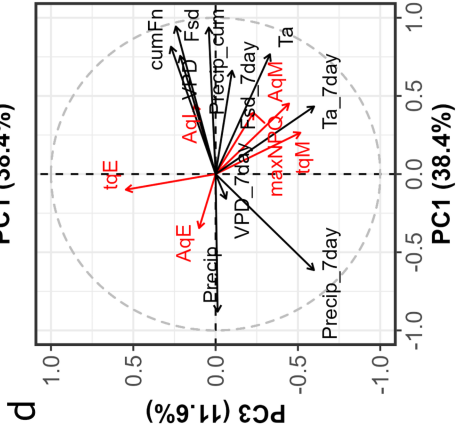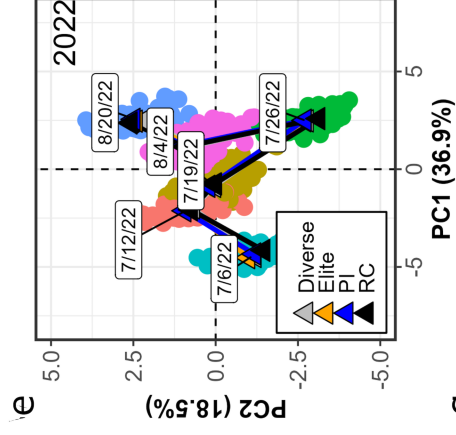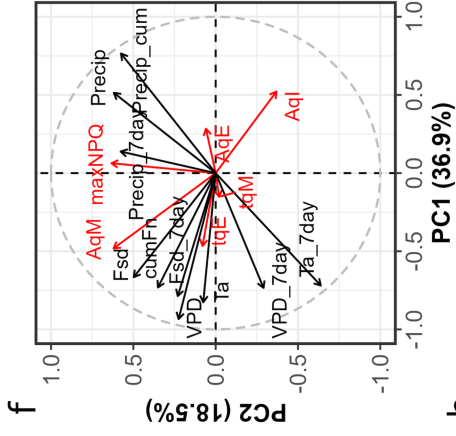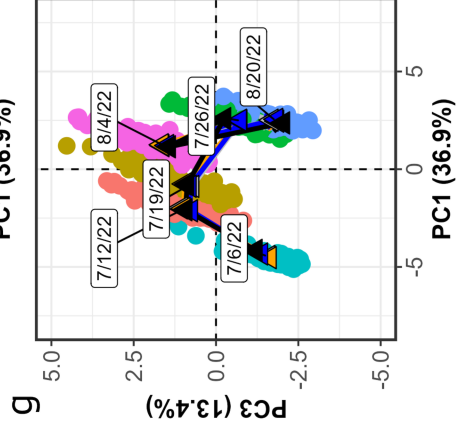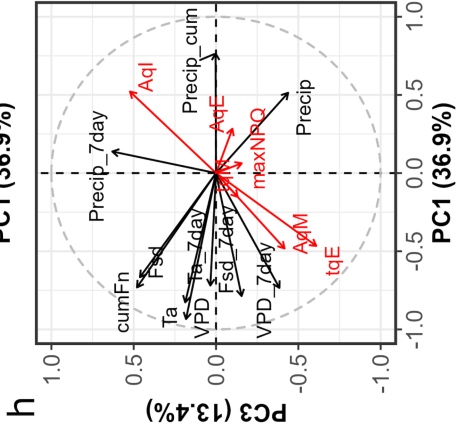

Supplement: Supplementary file 3 — Figure S1. Comparison of genotypic means for NPQ relaxation parameters measured in 2021 and 2022 using the SoyNAM founders. Scatterplots comparing values of (a) maximum inducible NPQ, (b) A qE, (c) A qM, (d) A qI, (e) τ qE, and (f) τ qM. Values represent the mean of seasonal measurements. Pearson correlation coefficient (R) and P‐value are reported for each parameter. Figure S2. Direct measurement of NPQ in NAM population founders grown in the field (July 23, 2024). (a) Comparison of rates of linear electron flow (LEFamb) against ambient PAR (PARamb). (b) Rates of LEF (LEFhigh) following 10 sec illumination at high light, compared to ambient PAR. (c) The difference between rates of LEF under ambient and high light, compared to ambient PAR. (d) Comparison of phiPSII measured under ambient and high light (yellow symbols), versus ambient PAR (gray symbols). Figure S3. Direct measurement of NPQ in NAM population founders grown in the field (July 30, 2024). (a) Comparison of rates of linear electron flow (LEFamb) against ambient PAR (PARamb). (b) Rates of LEF (LEFhigh) following 10 sec illumination at high light, compared to ambient PAR. (c) The difference between rates of LEF under ambient and high light, compared to ambient PAR. (d) Comparison of phiPSII measured under ambient and high light (yellow symbols), versus ambient PAR (gray symbols). Figure S4. Comparison of NPQt values for the SoyNAM founders on July 23, 2024. (a) Boxplot comparing NPQt values recorded for SoyNAM founders under high light. Values represent the mean of three technical (individual plant) replicates per plot (n = 5). (b) Comparison of NPQt measured under ambient light (PARamb) and NPQt (NPQtamb), individual technical replicates are shown. (c) Comparison of NPQt measured under high light (NPQthigh) and ambient PAR. Individual technical replicates are shown. Figure S5. Comparison of NPQt values for the SoyNAM founders on July 30, 2024. (a) Boxplot comparing NPQt values recorded for SoyNAM founder [file TPJ-121-0-s012.zip › 3_Figure_S22_PCA_years.pdf]
